# Deep learning for MRI-based acute and subacute ischaemic stroke lesion segmentation—a systematic review, meta-analysis, and pilot evaluation of key results

**DOI:** 10.3389/fmedt.2025.1491197

**Published:** 2025-06-10

**Authors:** Makram Baaklini, Maria del C. Valdés Hernández

**Affiliations:** ^1^ Edinburgh Imaging Academy, College of Medicine and Veterinary Medicine, University of Edinburgh, Edinburgh, United Kingdom; ^2^ Department of Neuroimaging Sciences, Centre for Clinical Brain Sciences, University of Edinburgh, Edinburgh, United Kingdom

**Keywords:** acute ischaemic stroke, deep learning, MRI, attention mechanisms, lesion segmentation

## Abstract

**Background:**

Segmentation of ischaemic stroke lesions from magnetic resonance images (MRI) remains a challenging task mainly due to the confounding appearance of these lesions with other pathologies, and variations in their presentation depending on the lesion stage (i.e., hyper-acute, acute, subacute and chronic). Works on the theme have been reviewed, but none of the reviews have addressed the seminal question on what would be the optimal architecture to address this challenge. We systematically reviewed the literature (2015–2023) for deep learning algorithms that segment acute and/or subacute stroke lesions on brain MRI seeking to address this question, meta-analysed the data extracted, and evaluated the results.

**Methods and materials:**

Our review, registered in PROSPERO (ID: CRD42023481551), involved a systematic search from January 2015 to December 2023 in the following databases: IEE Explore, MEDLINE, ScienceDirect, Web of Science, PubMed, Springer, and OpenReview.net. We extracted sample characteristics, stroke stage, imaging protocols, and algorithms, and meta-analysed the data extracted. We assessed the risk of bias using NIH's study quality assessment tool, and finally, evaluated our results using data from the ISLES-2015-SISS dataset.

**Results:**

From 1485 papers, 41 were ultimately retained. 13/41 studies incorporated attention mechanisms in their architecture, and 39/41 studies used the Dice Similarity Coefficient to assess algorithm performance. The generalisability of the algorithms reviewed was generally below par. In our pilot analysis, the UResNet50 configuration, which was developed based on the most comprehensive architectural components identified from the reviewed studies, demonstrated a better segmentation performance than the attention-based AG-UResNet50.

**Conclusion:**

We found no evidence that favours using attention mechanisms in deep learning architectures for acute stroke lesion segmentation on MRI data, and the use of a U-Net configuration with residual connections seems to be the most appropriate configuration for this task.

**Systematic Review Registration:**

https://www.crd.york.ac.uk/PROSPERO/view/CRD42023481551, PROSPERO CRD42023481551.

## 1 Introduction

Stroke remains a leading cause of mortality and long-term disability worldwide (1), placing a substantial burden on healthcare systems and societies (2). The majority of strokes are ischaemic (3). They can occur in different locations and are largely heterogeneous in appearance (3). After stroke onset, the progression of ischaemic injury continues for minutes-to-days, depending on brain region vulnerability, cellular constituents, and residual perfusion levels (4). There are three main stages used to describe the manifestations of stroke in radiological images: acute (less than 24 h), subacute (24 h to 5 days) and chronic (afterwards). Surrounding the ischaemic core, or irreversibly damaged tissue, appears a region that is functionally impaired, but potentially salvageable, known as ischaemic penumbra (5). Accurate diagnosis during acute-to-subacute stages allows for interventions (e.g., thrombolytic drugs or surgery) that may potentially salvage the penumbral area.

Magnetic resonance imaging (MRI) technology has not only enabled the non-invasive investigation of human brain features, but also of ischaemic injuries, thanks to the high dimensionality and particularly low signal-to-noise ratio found in MR images. Stroke lesions in the acute phase appear subtle in structural sequences but display very high intensities in diffusion weighted images (DWI) in most cases. Subacute strokes show greater mass effect, stronger and well-defined signal in structural sequences with well-defined margins, as well as in DWI in general. Segmentation of the infarcted regions in these images, as well as the normal tissues, has been important to advance stroke research and, ultimately, patient outcome. Since manual segmentation methods are time-consuming and subject to inter-rater variability, there has been a growing interest, since 2015 (6), in applying deep learning (DL) techniques to automate stroke lesion segmentation tasks and enhance their accuracy. DL methods can automatically extract intricate spatial and textural features within MR images, while requiring low-to-moderate subject matter expertise. DL also addresses long-dated machine learning-related challenges, such as discerning patterns in high-dimensional data, such as imaging data. To this end, various ischaemic lesion segmentation (ISLES) challenges have been taken place within the context of one of the major international medical image processing conferences: the Medical Image Computing and Computer Assisted Intervention (MICCAI), in years 2015, 2016, 2017, 2022, and 2024.

**Figure 1:**
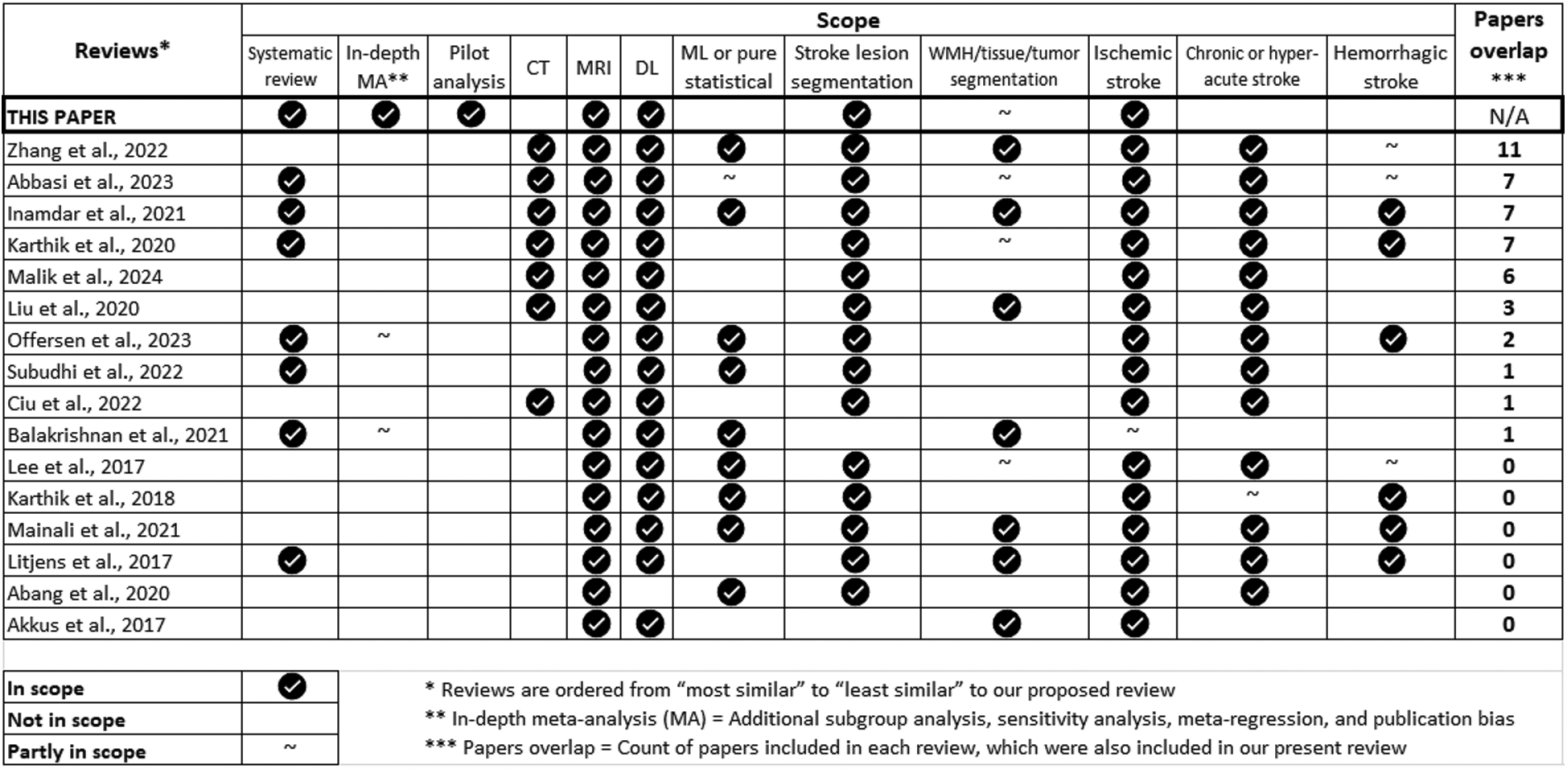
Summary of the scope of the review articles published from 2017 until 2023 that cover similar topics as the present review, and have contributing sources that partially overlap with the ones analysed here.

Not surprisingly, several methods have been proposed to automatically assess ischaemic lesions from MRI using DL. These have been analysed previously (Figure 1), but the data that pertains to segmentation of ischaemic stroke lesions have not been meta-analysed, nor their outcomes have been independently evaluated. We systematically review the literature from 2015 to 2023 to investigate the accuracy and generalisability of the proposed DL methods in acute-to-subacute stroke lesion segmentation on MRI, focusing on details of DL architectures and attention mechanisms, seeking to answer the following question: What would be the optimal DL model architecture for acute and subacute ischaemic stroke lesion segmentation on brain MRI? After meta-analysing the relevant data extracted from the sources reviewed, we conducted a pilot analysis to evaluate as many of the elements identified in the review as possible.

## 2 Background

### 2.1 Deep learning (DL) architectures

Convolutional neural networks (CNNs) are useful architectures for processing data with grid-like topology (e.g., 2D/3D grid of pixels/voxels) (7). They employ convolution blocks to produce “feature maps” through the use of sparse inter-layer interactions, with kernels smaller in size than the input (8). A standard convolutional block in a CNN (Supplementary Data Sheet 2 S1a) consists of a linear convolution operation on a kernel, which produces a feature map that is passed through an activation function to introduce non-linearity and enable the network to learn more complex relationships in the data (9), before it gets down-sampled by a pooling operation.

CNNs are widely used in medical image segmentation (10), with an architecture that typically ends with fully-connected layer(s) responsible for doing the predictions (e.g., pixel/tissue classification). Predictions are connected to a cost or loss function which measures their discrepancy with ground-truth data. Network parameters are then optimized through backpropagation, by minimizing the loss function until convergence, often aided by regularisation methods (9). However, (i) they produce feature maps with lower spatial dimensions than the input image, and (ii) they classify individual pixels using patches extracted around each pixel, and those often overlap significantly, which in turn creates redundancy in convolution operations. Fully Convolutional Networks (FCNs) address both drawbacks (i) by replacing CNN's fully-connected layer(s) with “up-sampling convolutions” that output images of the same size as the input, and (ii) by generating likelihood maps instead of pixel-by-pixel predictions. However, the FCN's output maps are of particularly low resolution (6).

U-Net architecture was first used for image segmentation in 2015 (11), and it has since achieved overwhelming success. It uses a symmetric encoder-decoder structure based on convolutional blocks, where down-sampling (encoder) operations compress images and up-sampling (decoder) operations restore them, until they reach the input image's original size (12), as opposed to FCNs. U-Nets also introduce skip connections that connect encoder-decoder layers of equal depth, hence allowing them to train with limited data while avoiding the vanishing gradient problem (13).

The ResNet architecture was published shortly after U-Net (14), to further tackle the vanishing gradient problem, also using skip connections. A standard ResNet block (Supplementary Data Sheet 2 S1b) consists of an “identity path” (green arrow in the figure) that can bypass the “residual path”, thus giving the network the option to simply copy activations to the next layer and preserve information when learned features do not require more depth. Skip connections also tackle the degradation issue, where adding layers leads to higher training error since accuracy gets “saturated” as the network keeps learning the data (15). ResNets can improve model convergence speed (16), but since most residual blocks only slightly change the input signal, they produce a large amount of redundant features (17). This is where DenseNets help.

The first DenseNet architecture was published shortly after ResNet (18). It employs dense connections interconnecting all layers in order to maximize information and gradient propagation (13). A standard Dense block is represented in Supplementary Data Sheet 2 S1c. Original inputs and activations from previous layers are both kept at each block, hence preserving the global state, while encouraging feature reuse with less network parameters (12). Reusing features across layers also allows DenseNets to tackle the vanishing gradient problem (19).

To solve the difficulties in optimizing network parameters, and given the impact of U-Net configurations, an out-of-the-box model that combines two basic types of networks: 2D U-Net and 3D U-Net in three different configurations to perform semantic segmentation of 3D images has gained popularity since its publication in 2021 due to its high level of performance in multiple biomedical applications. It is referred as nn-UNet (20) and owes its high performance to its architectural design that allows its self-configuration in any new given medical image segmentation task.

### 2.2 Attention mechanisms

When our eyes focus on a certain object, groups of filters within our visual perception system are used to create a blurring effect so that the object of interest is in focus, and the rest is blurred (21). Attention mechanisms attempt to achieve the same “blurring effect” but for machine-based image processing. Attention can capture the large receptive field and retrieve underlying contextual details by modelling the relationships between local and global features (22). The impact of incorporating attention mechanisms into a DL architecture has long been debated, yielding contradictory results (23–26). Also, it is not clear which way of incorporating attention will be more beneficial for a specific task. Therefore, to shed light on this issue for our particular purpose—ischaemic acute and subacute stroke lesion segmentation—we specifically extract and analyse the type and presence of attention mechanisms in the sources reviewed. In this work, we categorize attention mechanisms as “spatial”, “channel”, or “hybrid”.

“Spatial attention” (Supplementary Data Sheet 2 S2a) is responsible for generating masks that enhance the features that define a specified object (e.g., lesion) on a given feature map, therefore enhancing the input to subsequent layers of a network (21). Examples of spatial attention methods include attention gates, i.e., computational blocks to implement “attention” as described above; self-attention, which operates solely on input sequences, thus enabling a model to further exploit spatial relationships within input scans (27); and cross-attention [e.g., Gomez et al. (28)], which enables the network to simultaneously process encoder and decoder features, in order to pass the most aligned encoder features with respect to decoder features of same depth, and therefore decrease noisy signals in skip connections (27).

“Channel attention” (Supplementary Data Sheet 2 S2c) refers to the process of assigning a weight to each feature map or channel, emphasizing those that contribute most significantly to the learning (21). Conversely, spatial attention assigns weights to pixels. Each map specializes in detecting specific features (e.g., horizontal edges, brain anatomy). Examples of channel attention methods include squeeze-and-excitation blocks (29), which were used by Woo et al. (30) and Lee et al. (31). In summary, channel attention focuses on the importance of different feature maps, while spatial attention focuses on the importance of specific regions within a feature map.

“Hybrid attention” combines spatial and channel attention. Examples include dual attention gates, which combine spatial and channel attention gates (sAG + cAG) (32); and multi-head attention, which uses parallel processing by applying attention across multiple “heads” simultaneously, where each head may be configured to implement any channel or spatial attention operation (27).

## 3 Materials & methods

### 3.1 Protocol registration

We registered this systematic review protocol with the International Prospective Register of Systematic Reviews (PROSPERO), registration number: CRD42023481551 (November 2023). We conducted our review following the PRISMA guidelines (33, 34).

### 3.2 Search strategy

We conducted a literature search (January 2015–December 2023) for papers published in IEEE Explore, MEDLINE, ScienceDirect, Web of Science, PubMed, Springer, and OpenReview.net. We identified keywords by expanding five subject components: accuracy, acute ischaemic stroke, deep learning, lesion segmentation, and MRI.

We also did citation tracking of reviewed articles, and hand-searching of the two journals “Stroke” and “NeuroImage: Clinical” (Recall: 100%). Two reviewers (M.B. and M.C.V.H.) conducted the main search, paper selection, and data extraction, and discrepancies were resolved by discussion. The full search strategy is provided in Supplementary Data Sheet 1 A.

### 3.3 Eligibility criteria

Table 1 summarizes the selection criteria, justifying the basis for inclusion and exclusion of the different articles found during the search. Briefly, studies were included if presented (a) DL algorithm(s)/architecture(s) for segmenting ischaemic stroke lesions in acute and subacute phases in humans, from MRI, and were peer-reviewed and indexed in any of the databases searched. Studies were excluded otherwise.

**Table 1:** Study selection criteria.

	Inclusion criteria	Exclusion criteria	Rationale for inclusion/exclusion
Stroke types	Ischaemic	Haemorrhagic	Differences in clinical presentations, lesion appearances, & aetiologies
Stroke stages	• Acute • Subacute	• Hyperacute (unless in minor proportion in the dataset) • Chronic	Prioritize stages where MRI plays a more prominent role in diagnosis and treatment planning
Imaging	• All MRI modalities • All scanner types	• All CT modalities • Any other non-MRI modality	MRI allows *in vivo* assessment offering better soft tissue contrast & resolution than CT and PET
Algorithms	• All DL approaches (e.g., supervised, unsupervised) • Algorithms segmenting both: ischaemic core and penumbra	• Non-DL algorithms • Algorithms segmenting only WMH or brain tissue/tumours • Algorithms performing semi-automated segmentation (with human interaction) • Algorithms running on simulated/synthetic lesions	DL is the current state-of-the-art computational approach, much better than others at learning complex hierarchical features
Population	Humans (all ages/sexes)	• Non-human studies (e.g., animal-based) • Human studies using synthetic data	Human-based studies are more clinically relevant. Synthetic data may not fully capture variations and complexities of real clinical stroke lesions
Publishing	• Peer-reviewed studies • Proceedings of MICCAI, MIDL, and IEEE-led conferences • Publications in English • Publications between 2015 and 2023	• Pre-prints • Studies not available in any of the searched databases	To only retain the most reliable sources of information while also aiming for a wide readership
Completeness	Studies with sufficient information to be reproduced	Studies not reporting segmentation performance scores	Reproducibility is key in scientific research

### 3.4 Data extraction

For each paper, we extracted the following information: primary outcomes and measures, image acquisition protocol(s), sample characteristics, ground-truth data, data pre-processing, learning approach, model architecture, model training, model hyper-parameters, model validation, external validation, performance results, and generalisability of the proposed approach as per custom calculation. To cross-check data entry, a reviewer (M.C.V.H.) performed double extraction independently and blind to prior extraction results.

### 3.5 Data analysis

We analysed the extracted results using custom-built scripts in python. We calculated fixed-effects and random-effects as part of a whole group analysis. For these analyses we used the reported dice similarity coefficients (DSC) and their 95% confidence intervals (CI) to estimate the effect size. For the effect estimates we used the weighted average of the reported mean DSC. We further divided the studies in two groups: (i) studies using attention mechanisms, and (ii) studies not using attention mechanisms and repeated the analyses for each group. We also conducted a sensitivity analysis using the precision metric (instead of the DSC) to estimate the effect size. Lastly, we conducted a meta-regression analysis to assess whether there is statistically significant relationship between the presence of attention mechanisms and the likelihood of high mean DSC across studies. We further used the DSC and the standard errors for generating a funnel plot, followed by the Egger's test, to assess possible bias in the meta-analysis.

### 3.6 Publication quality analysis

We assessed the sources selected following the NIH's Study Quality Assessment Tool (https://www.nhlbi.nih.gov/health-topics/study-quality-assessment-tools).

### 3.7 Pilot analysis

We conducted a pilot analysis leveraging the findings from our literature analysis in an independent and publicly available sample. The specific aims of this pilot were two-fold: (1) proposing an architecture that leverages the findings of our systematic review in terms of best development practices: use 2D model with image-wise training, and increase network depth while leveraging the power of skip connections by combining U-Net and ResNet; and (2) to test, in the architectural choice that is most promising, the main points from the analyses (24 experiments conducted in total): with vs. without attention mechanisms, using a compound loss function vs. a region-based loss function, and using input images of a single modality (DWI) vs. input images of multiple modalities, to make informed recommendations for developers.

#### 3.7.1 Dataset

We used the ISLES-2015-SISS dataset, published by the MICCAI 2015 conference (35). It consists of brain MRI from 28 subacute stroke cases to use for model training. For each case, a set of five MRI sequences are provided: T1-weighted (T1-WI), T2-weighted (T2-WI), diffusion-weighted (DWI), and fluid-attenuated inversion recovery (FLAIR), along with the corresponding ground-truth masks. The data were already anonymised by removing patient information from files and facial bone structure from images.

#### 3.7.2 Data pre-processing

The following data pre-processing steps were conducted: intensity-based normalisation using Min-Max scaling, intensity-based skull-stripping using BET2 (performed by challenge organizers), rigid co-registration to the FLAIR sequences (performed by challenge organisers).

#### 3.7.3 Segmentation architecture, model training and evaluation

**Figure 2:**
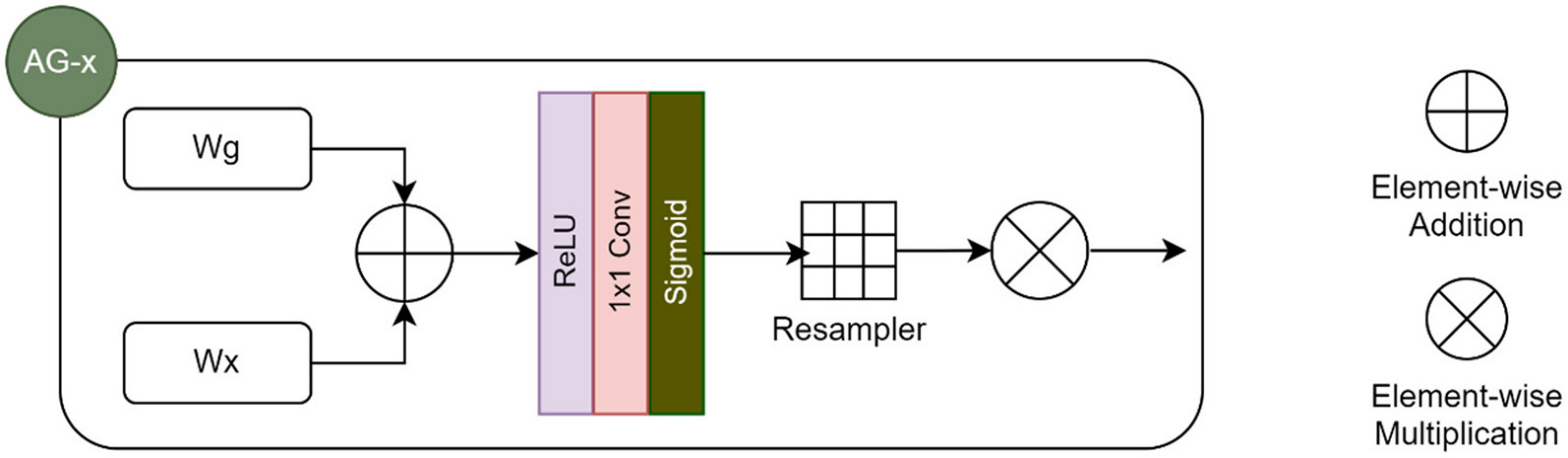
Architecture of an attention gate (AG), as used in our pilot analysis.

We implemented the DL architecture, AG-UResNet50, inspired by multiple papers (36–42), especially Guerrero et al.'s UResNet (39), Jin et al.'s RA-UNet (41), and Gheibi et al.'s CNN-Res (42). AG-UResNet50 is a five-level end-to-end U-Net (Supplementary Data Sheet 1 B1), with a ResNet50 replacing its encoder path (43). Using U-Net in combination with ResNet50 allows us to leverage the power of skip connections further (44), and make the network deeper. This makes it easier for the gradient to flow from output layers back to input during back-propagation, while handling the vanishing gradient problem. Zhang et al. (45) identified ResNet as an architecture that can improve segmentation of small lesions. Max-pooling was used for down-sampling the first set of feature maps produced by the model, since it can extract extreme features (e.g., lesion edges) well. Convolution blocks with stride two were used for remaining down-sampling operations, in order to better retain image details (13). On the decoder side, we simply used the U-Net's deconvolution blocks, but with Leaky ReLU activation instead of ReLU, in view of its better results in medical image analysis (46), as also demonstrated by Karthik et al. (47). We kept the up-sampling interpolation algorithm, which basically inserts new elements between pixels in the image matrix. Feature maps from the encoder are combined with those from the decoder in the same depth using concatenation. “Attention concatenation”, which was used here, works by incorporating attention gates (AGs) in skip connections (22), as seen in Karthik et al. (48), Nazari-Farsani et al. (49), and Yu et al. (50). An AG takes two input vectors that are added element-wise (Figure 2), resulting in aligned weights becoming larger and unaligned weights smaller. The output vector then goes through ReLU activation, 1 × 1 convolution, and sigmoid activation to produce the attention coefficients/weights. Coefficients are then up-sampled to the original dimensions of the input vector using trilinear interpolation, before being multiplied element-wise. The final output is passed along in the skip connection.

During training, we used a compound loss function mixing Binary Cross-Entropy (BCE) and Dice loss. BCE loss computed the gradient based on the difference in probability distribution of each pixel in the predicted vs. real sample (51), while Dice loss directly computed the gradient using the Dice score of predicted vs. real samples (18). From a regularisation standpoint, we used pixel dropout, learning rate adjustment and data augmentation methods, while for optimisation, we used Adam function and batch normalization. From a training infrastructure standpoint, the model was developed, trained and tested on Azure Databricks (python:Torch), using one sizeable driver: CPU:16 cores; OS:Ubuntu; RAM:56GB; Runtime:13.2ML. We evaluated the model performance using DSC, and used five-fold cross-validation. The full code used for this pilot is available from GitHub (https://github.com/Elpazzu/UoE-Pilot-Analysis/)

## 4 Results

### 4.1 Search results

**Figure 3:**
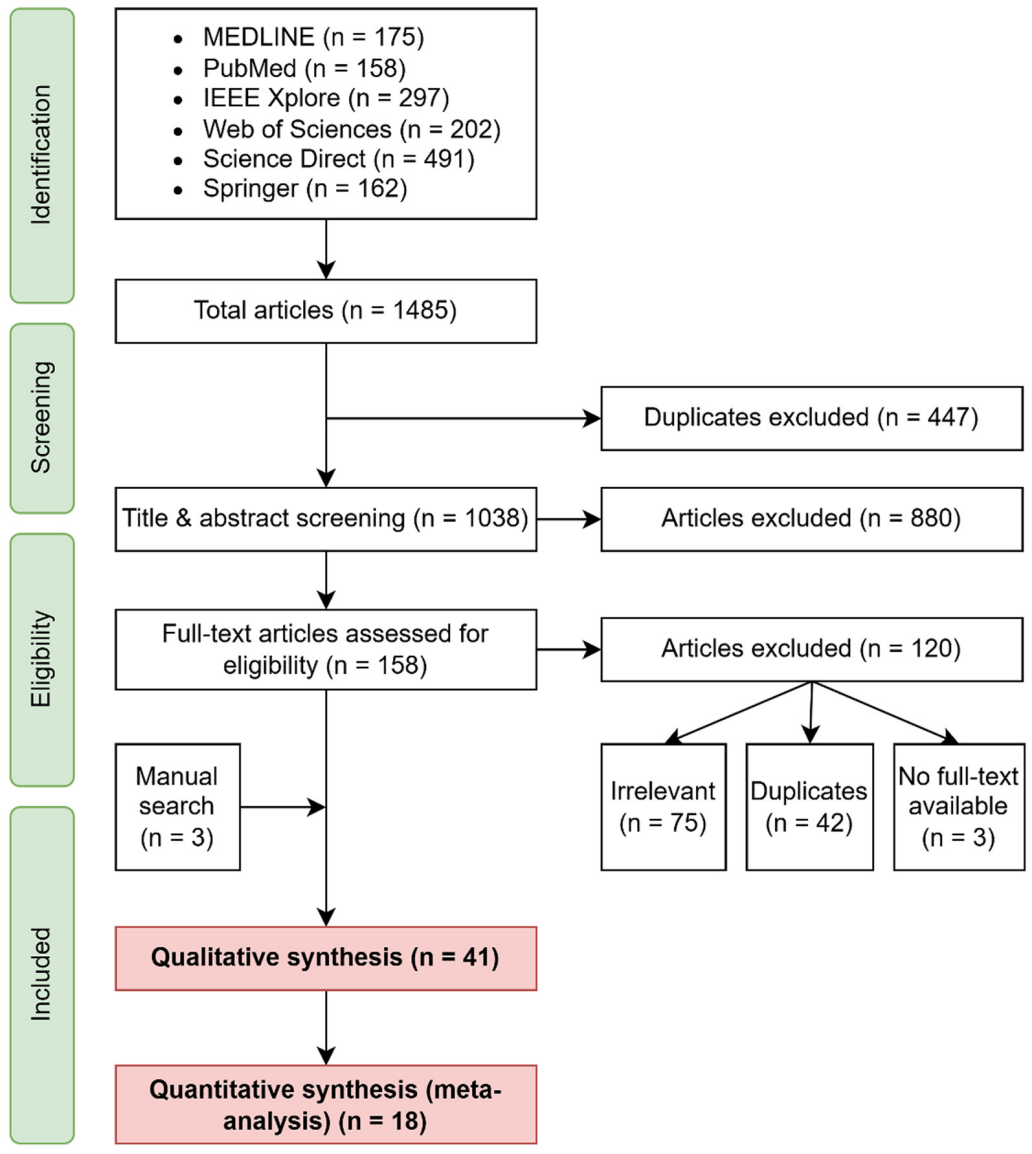
Flow chart of the identification, screening, and paper selection process.

The search yielded 1,485 papers, of which 41 were ultimately retained (Figure 3).

All papers had segmentation as primary outcome. Fewer had prognosis (6 studies) or functional (3 studies) outcomes. Prognosis studies were either trying to predict tissue fate or lesion volume [e.g., Wong et al. (52), Wei et al. (53)]. Functional studies mostly tried to predict the modified ranking scale score (mRS). Only one paper explicitly had diagnosis as primary outcome, but practically, segmentation and diagnosis are tightly linked, since by segmenting lesion pixels, the algorithm is effectively helping physicians with the diagnosis.

### 4.2 Sample characteristics

As Table 2 shows, patients were all adults of 18 years old and above, and males were generally slightly over-represented (58% on average), except in few studies where the opposite was true [e.g., Moon et al. (57)]. From a stroke severity standpoint, reported mean NIHSS (81) were always on the “minor” or “moderate” ranges (8 studies). Although both subacute and acute stroke stages were in scope, most studies (23/41) included exclusively acute ischaemic stroke cases. Reported patient mean “time-since-stroke” (TSS) were also exclusively in the acute interval, with 2 studies actually very close to the hyperacute-acute limit. Only four papers used sample sizes above 500 (Mean 252.2), and samples were most often collected from multiple centres (27 studies vs. 13 leveraging only one centre). Supplementary Data Sheet 2 S3 shows a graphical illustration of the sample characteristics.

**Table 2:** Characteristics of the samples of the studies included in the review. See full data extraction table in Supplementary Data C.

First author	Sample size	Number of medical centers	Stroke stage	Age range	Gender	Mean NIHSS	Mean stroke-to-MRI time	Mean lesion volume	Lesion volume ranges
		SC: single-center; MC: multi-center	Acute; Subacute		M: Male F: Female			(in ml)	(in ml)
Karthik et al. (47)	64	MC: 3	Subacute	[18+]	–	–	–	17.59	[1.0, 346.1]
Gómez et al. (28)	75	MC: 2	Acute	[18+]	–	–	–	37.83	[1.6, 160.4]
Olivier et al. (54)	929	MC: 6	Acute Subacute	[16–94]	M: 63.7% F: 36.3%	7.6	68.8h	21.84	–
Clèrigues et al. (55)	114	MC: 4	Acute Subacute	[18+]	–	–	–	SISS: 17.59 SPES: 133.21	SISS: [1.0, 346.1] SPES: [45.6, 252.2]
Liu et al. (56)	64	MC: 3	Subacute	[18+]	–	–	–	17.59	[1.0, 346.1]
Moon et al. (57)	79	-	Acute	–	M: 44.3% F: 55.7%	9.3	83.8h	–	[0.0, 250]
Zhang et al. (19)	242	SC: 1	Acute	[35–90]	M: 60.3% F: 39.7%	–	–	–	–
Wong et al. (52)	875	SC: 1	Acute	–	M: 48.9% F: 51.1%	6	-	–	–
Khezrpour et al. (58)	64	MC: 3	Subacute	[18+]	–	–	–	17.59	[1.0, 346.1]
Hu et al. (59)	75	MC: 2	Acute	[18+]	–	–	–	37.83	[1.6, 160.4]
Gheibi et al. (42)	44	MC: 2	Acute	–	–	–	–	–	–
Kumar et al. (60)	189	MC: 6	Acute Subacute	[18+]	*–*	*–*	*–*	SISS: 17.59 SPES: 133.21 IS17: 37.83	SISS: [1.0, 346.1] SPES: [45.6, 252.2] IS17: [1.6, 160.4]
Liu et al. (16)	79	MC: 2	Acute	[18+]	*–*	*–*	*–*	SPES: 133.21 LHC: -	SPES: [45.6, 252.2] LHC: -
Zhao et al. (61)	582	SC: 1	Acute	-	*–*	*–*	*–*	*–*	*–*
Liu et al. (32)	1,849	SC: 1	Acute Subacute	[52–73]	M: 52.9% F: 47.1%	3.4	17.7h	3.12	[1.55, 5.33]
Karthik et al. (48)	64	MC: 3	Subacute	[18+]	*–*	*–*	*–*	17.59	[1.0, 346.1]
Liu et al. (62)	114	MC: 4	Acute Subacute	[18+]	*–*	*–*	*–*	SISS: 17.59 SPES: 133.21	SISS: [1.0, 346.1] SPES: [45.6, 252.2]
Aboudi et al. (63)	64	MC: 3	Subacute	[18+]	*–*	*–*	*–*	17.59	[1.0, 346.1]
Pinto et al. (64)	75	MC: 2	Acute	[18+]	*–*	*–*	*–*	37.83	[1.6, 160.4]
Choi et al. (65)	54	MC: 2	Acute	[18+]	*–*	*–*	*–*	37.83	[1.6, 160.4]
Kim et al. (66)	296	SC: 1	Acute	[58–79]	M: 61.3% F: 38.7%	2.3	12.7h	12.19	[0.0, 279.4]
Woo et al. (30)	429	SC: 1	Acute	[24–98]	M: 62.3% F: 37.7%	*–*	21.4h	*–*	*–*
Lee et al. (31)	429	SC: 1	Acute	[24–98]	M: 62.3% F: 37.7%	*–*	21.4h	27.44	[0.3, 227.6]
Lee et al. (67)	472	SC: 1	Acute	[19+]	M: 63.3% F: 36.7%	3	4.9h	3.62	[0.52, 71.8]
Karthik et al. (68)	64	MC: 3	Subacute	[18+]	*–*	*–*	*–*	17.59	[1.0, 346.1]
Zhang et al. (69)	64	MC: 3	Subacute	[18+]	*–*	*–*	*–*	17.59	[1.0, 346.1]
Ou et al. (70)	99	SC: 1	Acute	*–*	*–*	*–*	*–*	*–*	*–*
Vupputuri et al. (71)	189	MC: 6	Acute Subacute	[18+]	*–*	*–*	*–*	SISS: 17.59 SPES: 133.21 IS17: 37.83	SISS: [1.0, 346.1] SPES: [45.6, 252.2] IS17: [1.6, 160.4]
Abdmouleh et al. (72)	64	MC: 3	Subacute	[18+]	*–*	*–*	*–*	17.59	[1.0, 346.1]
Duan et al. (73)	120	SC: 1	Acute	*–*	*–*	*–*	*–*	*–*	*–*
Lucas et al. (74)	75	MC: 2	Acute	[18+]	*–*	*–*	*–*	37.83	[1.6, 160.4]
Nazari-Farsani et al. (49)	445	MC: 6+	Acute	–	M: 50% F: 50%	13	6.2h	50	[15, 123]
Wei et al. (53)	216	SC: 1	Acute	–	M: 69.7% F: 30.3%	–	–	–	–
Li and Ji (75)	60	SC: 1	Acute	[49–88]	-	–	–	–	–
Liu et al. (76)	212	SC: 1	Acute Subacute	–	M: 62% F: 38%	–	–	–	–
Cornelio et al. (77)	75	MC: 2	Acute	[18+]	-	–	–	37.83	[1.6, 160.4]
Yu et al. (50)	182	MC: 6+	Acute	–	M: 46.7% F: 53.3%	15	-	54	[16, 117]
Wu et al. (78)	400	MC: 3	Subacute	[18+]	–	–	–	27.94	[0.0575, 340.28]
Guerrero et al. (39)	250	SC: 1	Acute	–	–	–	–	–	–
Jeong et al. (79)	400	MC: 3	Subacute	[18+]	–	–	–	27.94	[0.0575, 340.28]
Gui et al. (80)	400	MC: 3	Subacute	[18+]	–	–	–	27.94	[0.0575, 340.28]

### 4.3 Imaging acquisition and manipulation

**Figure 4:**
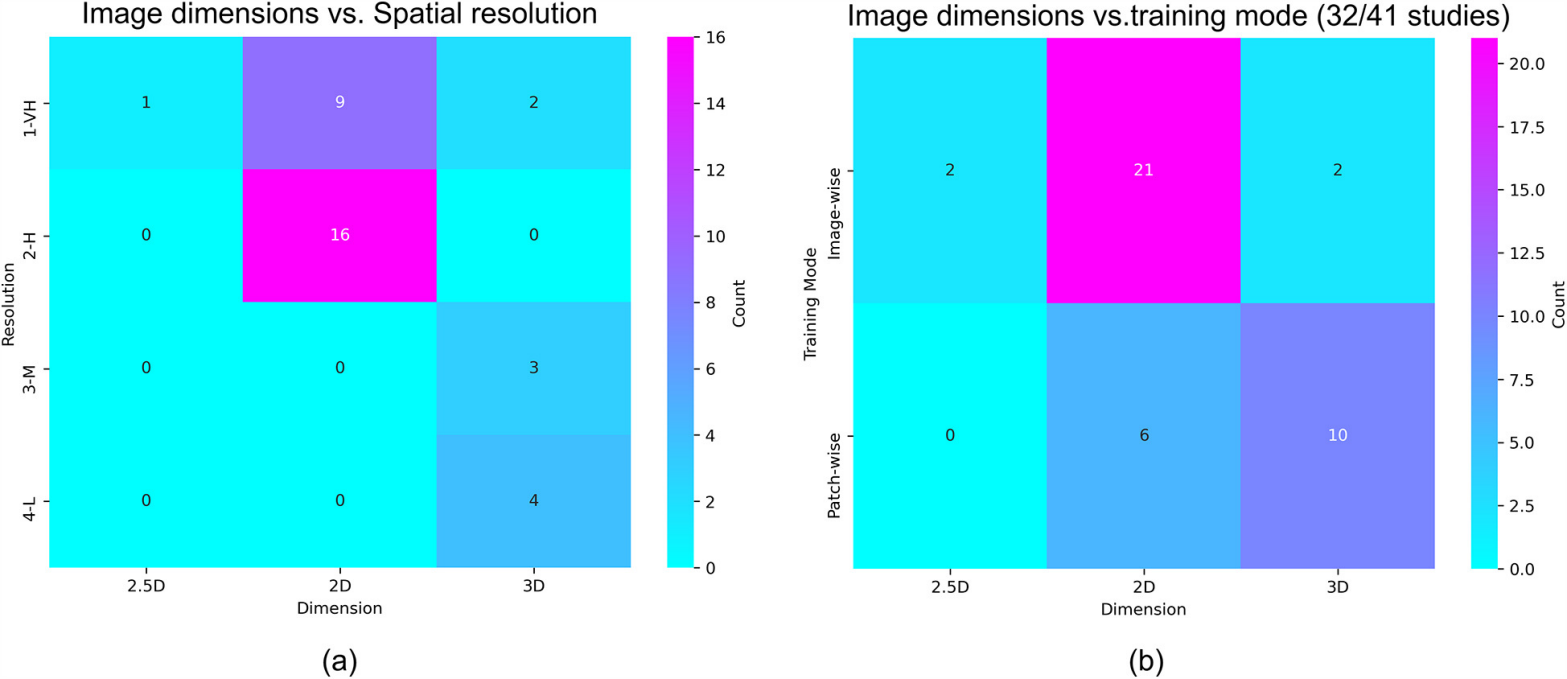
**(a)** Correlation between the dimension and the spatial resolution of input images; **(b)** correlation between the dimension of input images and the adopted model training mode.

Table 3 shows the imaging data extracted from the reviewed sources, and Figure 4 plots the correspondence between the dimensions of the images used as input to the reviewed algorithms (i.e., 2D, 2.5D, or 3D) and the spatial resolution and the manipulation of these images during training (i.e., patch-wise or image-wise). Most studies (27/41) used images of high or very high spatial resolution. DWI modality was by far the most used modality (39 studies), followed by FLAIR (19 studies). Also, most studies (28/41) adopted a multimodal approach, applying image fusion early (25 studies), late (2 studies), or in a hybrid manner (1 study). Twenty-seven studies used a 2D-based approach and twelve a 3D-based approach (Table 3). 2D models exclusively used high- or very high-resolution images, whereas 3D models used mostly moderate- or low-resolution images, which seems counter intuitive (Figure 4a). 3D models adopted patch-wise training in 10/12 studies (Figure 4b). Most studies (25/41) reported mismatch between the stroke lesion borders on different image sequences; 15 to DWI-FLAIR mismatch, and 12 studies referred to diffusion-perfusion (DWI-PWI) mismatch. The magnetic field of the scanner(s) was 1.5 T and 3 T in 27 studies, only 3 T in nine studies, and only 1.5 T in three studies. See pie charts in Supplementary Data Sheet 1 B.

**Table 3:** Imaging acquisition and manipulation in the reviewed studies.

First author	Spatial resolution	Image modalities (& Image fusion)	Input dimension	Modality mismatch	Magnetic field
	1-Very High (VH); 2-High (H); 3-Moderate (M); 4-Low (L)	Image modalities: SM: Single-modality; MM: Multi-modality Image fusion: Early; Late; Hybrid Format: Modality: {Parameter} {Fusion time}	2D; 2.5D; 3D	T1-T2; DWI-PWI; DWI-FLAIR; T2-FLAIR; T1-FLAIR	1.5T; 3T
Karthik et al. (47)	2-H	MM: {FLAIR, T2WI, T1WI, DWI-b1000} {Early}	2D	DWI-FLAIR	3T
Gómez et al. (28)	2-H	MM: {DWI-ADC, PWI-rCBF, PWI-rCBV, PWI-MTT, PWI-TTP, PWI-Tmax, Raw 4D PWI} {Early}	2D	DWI-PWI	1.5T 3T
Olivier et al. (54)	Not reported	SM: {DWI-b0, DWI-b1000, DWI-ADC} {N/A}	3D	None reported	1.5T 3T
Clèrigues et al. (55)	4-L	MM: {FLAIR, T1WI, T2WI, DWI-b1000, PWI-CBF, PWI-CBV, PWI-TTP, PWI-Tmax} {Early}	3D	DWI-PWI; DWI-FLAIR	1.5T 3T
Liu et al. (56)	2-H	MM: {FLAIR, DWI-b1000} {Early}	2D	DWI-FLAIR	3T
Moon et al. (57)	1-VH	MM: {FLAIR, DWI-b1000} {Early}	2D	None reported	1.5T
Zhang et al. (19)	3-M	SM: {DWI-b0, DWI-b1000, DWI-ADC} {N/A}	3D	None reported	1.5T 3T
Wong et al. (52)	Not reported	SM: {DWI-b0, DWI-b1000, DWI-eADC} {N/A}	2D	None reported	1.5T 3T
Khezrpour et al. (58)	2-H	SM: {FLAIR} {N/A}	2D	DWI-FLAIR	3T
Hu et al. (59)	4-L	MM: {DWI-ADC, PWI-rCBF, PWI-rCBV, PWI-MTT, PWI-TTP, PWI-Tmax, Raw 4D PWI} {Early}	3D	DWI-PWI	1.5T 3T
Gheibi et al. (42)	Not reported	MM: {FLAIR, DWI} {Early}	2D	None reported	–
Kumar et al. (60)	4-L	MM: {FLAIR, T2WI, T1WI, DWI-b1000, PWI-CBF, PWI-CBV, PWI-TTP, PWI-Tmax, DWI-ADC, PWI-rCBF, PWI-rCBV, PWI-MTT, Raw 4D PWI} {Early}	3D	DWI-PWI; DWI-FLAIR	1.5T 3T
Liu et al. (16)	2-H	MM: {T1WI, T2WI, DWI-b1000, PWI-CBF, PWI-CBV, PWI-TTP, PWI-Tmax} {Early}	2D	DWI-PWI	1.5T 3T
Zhao et al. (61)	2-H	SM: {DWI-ADC, DWI-b0, DWI-b1000} {N/A}	2D	None reported	1.5T 3T
Liu et al. (32)	3-M	SM: {DWI-b0, DWI-ADC, DWI-IS} {N/A}	3D	None reported	1.5T 3T
Karthik et al. (48)	2-H	MM: {FLAIR, T2WI, T1WI, DWI-b1000} {Early}	2D	DWI-FLAIR	3T
Liu et al. (62)	2-H	MM: {FLAIR, DWI-b1000, PWI-CBF, PWI-CBV, PWI-TTP, PWI-Tmax} {Early}	2D	DWI-PWI; DWI-FLAIR	1.5T 3T
Aboudi et al. (63)	2-H	MM: {FLAIR, T2WI, T1WI, DWI-b1000} {Early}	2D	DWI-FLAIR	3T
Pinto et al. (64)	2-H	MM: {DWI-ADC, PWI-rCBF, PWI-rCBV, PWI-MTT, PWI-TTP, PWI-Tmax, Raw 4D PWI} {Late}	2D	DWI-PWI	1.5T 3T
Choi et al. (65)	4-L	MM: {DWI-ADC, PWI-rCBF, PWI-rCBV, PWI-MTT, PWI-TTP, PWI-Tmax, Raw 4D PWI} {Early}	3D	DWI-PWI	1.5T 3T
Kim et al. (66)	1-VH	SM: {DWI-b0, DWI-b1000, DWI-ADC} {N/A}	2D	None reported	1.5T 3T
Woo et al. (30)	1-VH	SM: {DWI-b1000, DWI-b0, DWI-ADC} {N/A}	2D	None reported	1.5T 3T
Lee et al. (31)	1-VH	SM: {DWI-b1000, DWI-b0, DWI-ADC} {N/A}	2D	None reported	1.5T 3T
Lee et al. (67)	3-M	MM: [DWI, DWI-ADC, FLAIR, PWI-Tmax, PWI-TTP, Pred(init)] {Early}	3D	DWI-PWI	1.5T 3T
Karthik et al. (68)	2-H	MM: {FLAIR, T2WI, T1WI, DWI-b1000} {Early}	2D	DWI-FLAIR	3T
Zhang et al. (69)	2-H	SM: {DWI-b1000} {N/A}	2D	DWI-FLAIR	3T
Ou et al. (70)	1-VH	SM: {DWI-b1000, DWI-eADC} {N/A}	2.5D	None reported	1.5T 3T
Vupputuri e tal. (71)	2-H	MM: {FLAIR, PWI-CBF, PWI-CBV, PWI-TTP, PWI-Tmax, DWI-ADC, PWI-rCBF, PWI-rCBV, PWI-MTT, Raw 4D PWI} {Early}	2D	DWI-PWI; DWI-FLAIR	1.5T 3T
Abdmouleh et al. (72)	2-H	MM: {FLAIR, T2WI, T1WI, DWI-b1000} {Early}	2D	DWI-FLAIR	3T
Duan et al. (73)	Not reported	MM: {T2WI, DWI-b1000, DWI-b0} {Late}	3D	None reported	–
Lucas et al. (74)	2-H	MM: {DWI-ADC, PWI-rCBF, PWI-rCBV, PWI-MTT, PWI-TTP, PWI-Tmax, Raw 4D PWI} {Early}	2D	DWI-PWI	1.5T 3T
Nazari-Farsani et al. (49)	Not reported	SM: {DWI-b1000, DWI-ADC} {N/A}	3D	None reported	1.5T 3T
Wei et al. (53)	1-VH	SM: {DWI-b1000} {N/A}	2D	T1-T2; T2-FLAIR; T1-FLAIR	3T
Li and Ji (75)	1-VH	MM: {T1WI, T2WI, T2WI-FLAIR, DWI, DWI-ADC} {Early}	2D	T2-FLAIR; T1-FLAIR	1.5T
Liu et al. (76)	Not reported	MM: {T2WI, DWI, DWI-ADC} {Early}	2D	None reported	1.5T 3T
Cornelio et al. (77)	2-H	MM: {DWI-ADC, PWI-rCBF, PWI-rCBV, PWI-MTT, PWI-TTP, PWI-Tmax, Raw 4D PWI} {Early}	2D	DWI-PWI	1.5T 3T
Yu et al. (50)	Not reported	MM: {DWI-b1000, DWI-ADC, PWI-Tmax, PWI-MTT, PWI-CBF, PWI-CBV} {Early}	2.5D	None reported	1.5T 3T
Wu et al. (78)	1-VH	MM: {DWI-b1000, DWI-ADC, FLAIR} {Early}	2D	DWI-FLAIR	1.5T 3T
Guerrero et al. (39)	1-VH	MM: {FLAIR, T1WI} {Early}	2D	None reported	1.5T
Jeong et al. (79)	1-VH	MM: {DWI-b1000, DWI-ADC, FLAIR} {Hybrid}	3D	DWI-FLAIR	1.5T 3T
Gui et al. (80)	1-VH	MM: {DWI-b1000, DWI-ADC, FLAIR} {Early}	3D	DWI-FLAIR	1.5T 3T

### 4.4 Data pre-processing

Eighteen studies used proprietary datasets (Table 4), 22 used one or a combination of ISLES-2015 (35), ISLES-2017 (82) or ISLES-2022 (4), and two used data related to the DEFUSE or iCAS studies (83–85). In relation to skull-stripping, 37 studies performed an intensity-based approach (using BET2/ITK software), one study used an atlas-based approach [Moon et al. (57) using Kirby/MMRR template], and one study used DL to reduce sensitivity-to-noise (86) [Liu et al. (32) using in-house “UNet BrainMask”]. Inter-patient image registration onto a standard space (e.g., MINI) and/or intra-patient registration (e.g., registration of different sequences) were performed in 29 studies. Notably, Gui et al. (80) introduced the unsupervised, attention-based ConvNXMorph model to perform cascaded image registration before feeding the data into the segmentation algorithm.

**Table 4:** Data pre-processing in the reviewed studies.

First author		Data pre-processing methods				
	Dataset	Intensity-based	Atlas-based	Morphology-based	Deformable surface-based	Machine learning-based
	Dataset used for model training	Data pre-processing techniques used prior to model training				
Karthik et al. (47)	ISLES2015 SISS	- Normalization - - Skull-stripping	–	- Resizing	- Registration	–
Gómez et al. (28)	ISLES2017	- Normalization - Contrast adjustment - Skull-stripping	–	- Resizing - Rescaling	- Registration	–
Olivier et al. (54)	Proprietary	- Normalization	–	- Rescaling - Zero-padding - Cropping	–	–
Clèrigues et al. (55)	ISLES2015 SISS ISLES2015 SPES	- Normalization - Skull-stripping	–	–	- Registration	–
Liu et al. (56)	ISLES2015 SISS	- Skull-stripping	–	–	- Registration	–
Moon et al. (57)	Proprietary	- Normalization	- Skull-stripping	- Zero-padding - Resizing	- Registration	–
Zhang et al. (19)	Proprietary	- Normalization	–	- Zero-padding - Cropping Resizing	–	–
Wong et al. (52)	Proprietary	- Normalization	–	–	–	–
Khezrpour et al. (58)	ISLES2015 SISS	- Contrast adjustment - RGB to greyscale - Skull-stripping	–	- Cropping - Resizing	- Registration	–
Hu et al. (59)	ISLES2017	- Skull-stripping	–	- Resizing - Cropping	- Registration	–
Gheibi et al. (42)	Proprietary	–	–	- Zero-padding	- Splitting into 2D	–
Kumar et al (60)	ISLES2015 SPES ISLES2015 SSIS ISLES2017	- Normalization - Skull-stripping		- Resizing	- Converting to 3D - Registration	- Slice classification
Liu et al. (16)	ISLES2015 SPES Proprietary	- Normalization - Smoothing - Skull-stripping	–	–	- Registration	–
Zhao et al. (61)	Proprietary	- Normalization	–	–	–	–
Liu et al. (32)	Proprietary	- Normalization	–	- Resizing - Rescaling	–	- Slice classification - Skull-stripping
Karthik et al. (48)	ISLES2015 SISS	- Skull-stripping	–	–	- Registration	–
Liu et al (62)	ISLES2015 SPES ISLES2015 SISS	- Normalization - Skull-stripping	–	- Zero-padding - Cropping - Resizing	- Registration - Splitting into 2D	–
Aboudi et al. (63)	ISLES2015 SISS	- RGB to greyscale - Skull-stripping	–	- Resizing - Rescaling	- Registration	–
Pinto et al. (64)	ISLES2017	- Normalization - Bias field correction - - Skull-stripping	–	- Resizing	- - Registration	–
Choi et al. (65)	ISLES2016	- Normalization - Skull-stripping	–	- Resizing - Rescaling	- Registration	–
Kim et al. (66)	Proprietary	- Normalization	–	- Resizing	–	–
Woo et al. (30)	Proprietary	- Normalization	–	–	- Registration	–
Lee et al. (31)	Proprietary	- Normalization	–	- Resizing	- Registration	–
Lee et al. (67)	Proprietary	–	–	- Resizing - Rescaling	- Registration	-
Karthik et al. (68)	ISLES2015 SISS	- Normalization - Skull-stripping	–	- Cropping - Rescaling	- Registration	- Slice classification
Zhang et al. (69)	ISLES2015 SISS	- Normalization - Skull-stripping	–	- Cropping Rescaling	- Registration	- Slice classification
Ou et al. (70)	Proprietary	- Normalization - Skull-stripping	–	- Resizing	–	–
Vupputuri et al. (71)	ISLES2015 SPES ISLES2015 SISS ISLES2017 (IS17)	- RGB to greyscale - Normalization - Skull-stripping	–	–	- Registration	–
Abdmouleh et al. (72)	ISLES2015 SISS	- Normalization - - Skull-stripping	–	–	- Registration	–
Duan et al. (73)	Proprietary	- Normalization - Skull-stripping	–	- Resizing	–	–
Lucas et al. (74)	ISLES2017	- Skull-stripping	–	- Rescaling	- Registration	–
Nazari-Farsani et al. (49)	UCLA iCAS DEFUSE DEFUSE-2	- Normalization	–	-	- Registration	–
Wei et al. (53)	Proprietary	- Skull-stripping	–	- Rescaling	- Registration	–
Li and Ji (75)	Proprietary	–	–	- Resizing	–	–
Liu et al. (62)	Proprietary	- Normalization - - Skull-stripping	–	- Cropping - Resizing	- Registration	–
Cornelio et al. (77)	ISLES2017	- RGB to greyscale - Contrast adjustment - Normalization - - Skull-stripping	–	- Resizing	- Registration	–
Yu et al. (50)	iCAS DEFUSE-2	- Normalization	–	–	- Registration	–
Wu et al. (78)	ISLES2022	- Skull-stripping	–	- Resizing Rescaling	- Registration	–
Guerrero et al. (39)	Proprietary	- Normalization	–	- Resizing	- Registration	–
Jeong et al. (79)	ISLES2022	- Skull-stripping	–	- Resizing - Rescaling	- Registration	–
Gui et al. (80)	ISLES2022	- Skull-stripping	–	- Resizing - Rescaling	–	- Registration

### 4.5 Deep learning (DL) architectures

**Figure 5:**
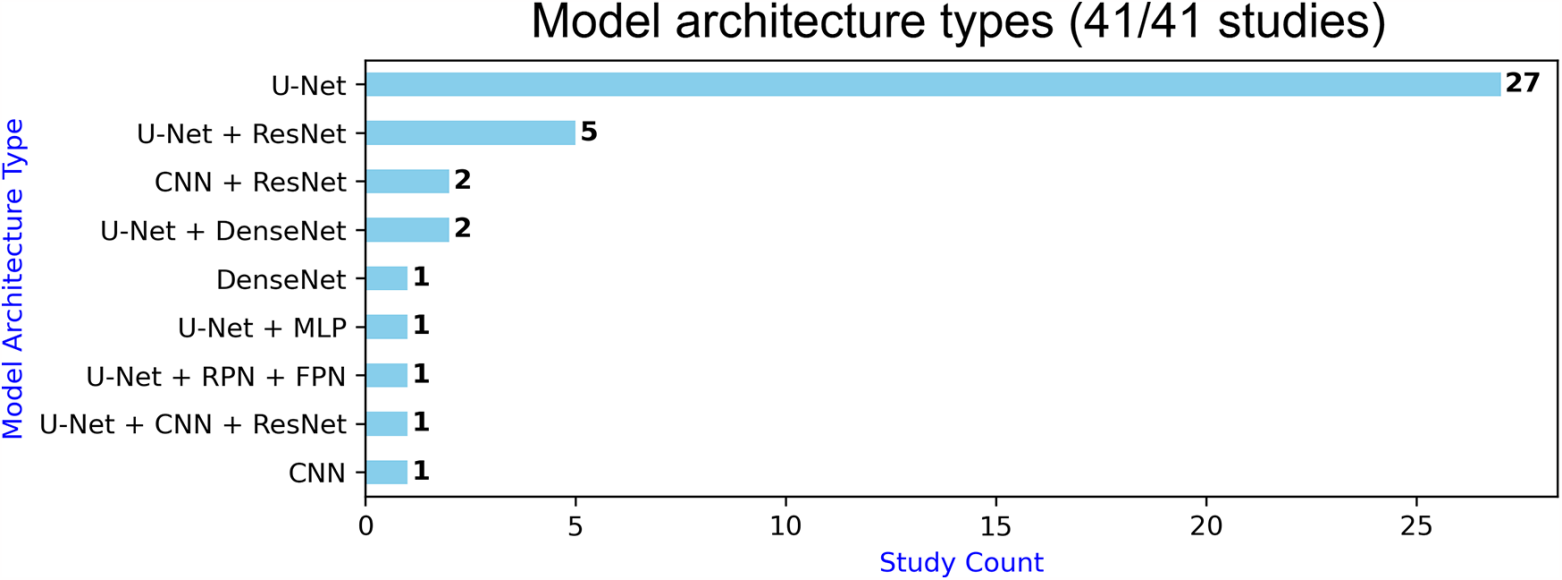
Model architecture types.

Within the 39/41 studies that performed semantic segmentation, 37 studies used U-Net-based models (Figure 5). But none of them used the original U-Net as-is (11), with perhaps Cornelio et al. (77) and Aboudi et al. (63) being the closest. ResNet architecture was the second most used (8 studies), while DenseNets were only used in three studies. Data augmentation was the most used regularisation method (30 studies), whereas each of dropout, early stopping, weight decay, class weighting, and learning rate adjustment were used in 9–13 studies. More papers used image-wise training (27 studies vs. 16 for patch-wise training); 7/8 studies that were dealing with smaller mean lesion volumes (<40 ml) used patch-wise training. In addition, none of the papers performed uncertainty quantification, and 32 algorithms were end-to-end (vs. 9 multi-module). Twenty-five studies used Dice loss (Table 5), either mixed with other loss functions (10 studies) or standalone (15 studies). Cross-entropy loss was used in 19 papers, nine times standalone. Focal loss was only used in four papers, and two papers used Liu et al.'s custom-built loss function (16). Twelve studies used attention: five used hybrid attention, four spatial attention, and three used channel attention (Table 5). Studies deploying ResNet-based architectures did not incorporate attention. Four studies embedded deep supervision layers within their U-Net architecture, effectively applying auxiliary supervision to intermediate decoder outputs (i.e., lesion masks) in order to refine feature representation. Such layers are also part of the self-configuring and task-agnostic nnU-Net model (20), which was leveraged by two studies in our review, both on 3D image inputs.

**Table 5:** Deep learning (DL) architectures of the models presented in the studies included (see corresponding summary graphs in the Supplementary Data Sheet 1 B).

First author	Architecture (segmentation type)	Loss function	Attention mechanism/type	Activation functions	Regularisation method	Optimisation method	Epochs
Karthik et al. (47)	U-Net (Semantic)	Dice	None	Leaky ReLU, ReLU, Softmax	Data augmentation	Adam	120
Gómez et al. (28)	U-Net (Semantic)	Focal	Additive cross-attention/spatial	ReLU, Sigmoid	- Data augmentation - Weight decay - Class weighting	AdamW	600
Olivier et al. (54)	U-Net (Semantic)	Dice	None	Leaky ReLU, Softmax	- Data augmentation - ES on validation loss	Adam	–
Clèrigues et al. (55)	U-Net (Semantic)	Focal	None	PReLU, Softmax	- Data augmentation - ES on MAE/L1 loss - Dropout - Class weighting	AdaDelta	–
Liu et al. (56)	U-Net (Semantic)	Dice	Self-gated soft attention/hybrid	ReLU, Sigmoid	- Data augmentation - Dropout	Adam	150
Moon et al. (57)	U-Net (Semantic)	BCE	None	ReLU, Sigmoid	–	Adam	200
Zhang et al. (19)	DenseNet (Semantic)	Dice	None	ReLU, Softmax	- Data augmentation - Weight decay - Learning rate adjust.	SGD	2,000
Wong et al. (52)	U-Net (Semantic)	Dice	None	ReLU,?	- Data augmentation	–	–
Khezrpour et al. (58)	U-Net (Semantic)	Dice	None	ReLU, Sigmoid	- Data augmentation - ES on validation loss	Adam	–
Hu et al. (59)	U-Net + ResNet (Semantic)	Focal	None	ReLU, Sigmoid	- Data augmentation - Class weighting	Adam	1,500
Gheibi et al. (42)	U-Net + ResNet (Semantic)	Custom	None	ReLU, Sigmoid	- Data augmentation - Weight decay - Dilution	Adam	–
Kumar et al. (60)	U-Net (Semantic)	BCE-Dice	None	ReLU, Softmax	- Data augmentation - Dropout - ES on validation set - Learning rate adjust.	Adam	200
Liu et al. (16)	U-Net + ResNet (Semantic)	Custom	None	Leaky ReLU, Sigmoid	- Data augmentation	–	70
Zhao et al. (61)	CNN (Semantic)	BCE	Squeeze-excitation/channel	ReLU, Sigmoid	- Data augmentation - - ES on validation loss	RAdam	–
Liu et al. (32)	U-Net (Semantic)	BCE-Dice	Dual attention gates/hybrid	SeLU (Self-normalized), Sigmoid	- Weight decay - ES on training & val. - Learning rate adjust. - Class weighting	Adam	200
Karthik et al. (48)	U-Net (Semantic)	Dice	Attention gates/spatial	ReLU, Sigmoid	- Data augmentation	–	150
Liu, L. (62)	U-Net + DenseNet (Semantic)	CE-Dice	None	ReLU, Sigmoid	- Data augmentation - Dropout	Adam	8
Aboudi, F. (63)	U-Net (Semantic)	CE	None	ReLU, Sigmoid	- Data augmentation	Adam	100
Pinto et al. (64)	U-Net (Semantic)	Dice	None	–	–	Adam	–
Choi et al. (65)	U-Net + CNN + ResNet (Semantic)	CE-Dice	None	ReLU, Softmax	- Data augmentation - Weight decay - Dropout - *ES*	Adam	–
Kim et al. (66)	U-Net (Semantic)	Dice	None	ReLU, Sigmoid	–	Adam	1,000
Woo et al. (30)	U-Net + DenseNet (Semantic)	–	Squeeze-excitation/channel	ReLU, Sigmoid	–	–	–
Lee et al. (31)	U-Net (Semantic)	Dice	Squeeze-excitation/channel	ReLU, Sigmoid	–	–	–
Lee et al. (67)	U-Net (Semantic)	Dice	None	ReLU, Sigmoid	ES on validation loss	Adam	–
Karthik et al. (68)	U-Net (Semantic)	Dice-CE + Softmax-CE	Multi-residual attention/hybrid	ReLU, Softmax	- Data augmentation - Masked dropout - Dropout - Learning rate adjust.	Adam	150
Zhang et al. (69)	U-Net (Semantic + Instance)	CE	None	ReLU, Softmax	- Data augmentation - Momentum - Weight decay	SGD	–
Ou et al. (70)	U-Net (Semantic)	BCE	None	ReLU, Softmax	–	RMSprop	100
Vupputuri et al. (71)	U-Net (Semantic)	BCE	Multi-path attention/hybrid (includes self-attention)	Leaky ReLU, Softmax	- ES on validation set - Dropout	Adam	30
Abdmouleh et al. (72)	U-Net (Semantic)	CE	None	ReLU, Sigmoid	Data augmentation	Adam	20
Duan et al. (73)	CNN + ResNet (Semantic)	Dice-CE	None	PReLU, Softmax	Data augmentation	Adam	600
Lucas et al. (74)	U-Net (Semantic)	Soft QDice	None	ReLU, Sigmoid	Data augmentation	Adam	100
Nazari-Farsani et al. (49)	U-Net (Semantic)	BCE-Volume-MAE-Dice	Attention gates/spatial	ReLU, Sigmoid	- Data augmentation - Class weighting - Dropout	Adam	80
Wei et al. (53)	U-Net + ResNet (Semantic)	Focal Tversky	None	ReLU, Softmax	- Data augmentation - Class weighting - Learning rate adjust.	Adam	150
Li and Ji (75)	U-Net (Instance)	CE	None	ReLU, Sigmoid	- Data augmentation - Class weighting	SGD	200
Liu et al. (76)	CNN + ResNet (Semantic)	Dice	None	ReLU, Sigmoid	- Data augmentation - Weight decay - Learning rate adjust.	Adam	500
Cornelio et al. (77)	U-Net (Semantic)	Dice	None	ReLU, Sigmoid	- Dropout - Weight decay	Adam	50
Yu et al. (50)	U-Net (Semantic)	BCE-Volume-MAE-Dice	Attention gates/spatial	ReLU, Sigmoid	- Data augmentation - Class weighting - Dropout	Adam	120
Wu et al. (78)	U-Net + MLP (Semantic)	Dice + Boundary	Multi-head self-attention/hybrid (includes self-attention)	ReLU, Softmax	- Weight decay - Learning rate adjust	AdamW	35
Guerrero et al. (39)	U-Net + ResNet (Semantic)	CCE	None	ReLU, Softmax	- Data augmentation - Class weighting - Learning rate adjust	Adam	–
Jeong et al. (79)	Ensemble of 2 (nn)U-Nets (Semantic)	Soft Dice-BCE	None	ReLU, Sigmoid	- Weight decay - Momentum - Learning rate adjust. - Data augmentation - Dropout	Adam	1,000
Gui et al. (80)	(nn)U-Net (Semantic)	Soft Dice-BCE	None	Leaky ReLU, Sigmoid	- Data augmentation - Dropout	AdamW	300

### 4.6 Performance and generalisability

As Table 6 shows, the performance metrics most frequently used across the studies reviewed were the overlap metrics Dice, Recall, and Precision, as well as the Hausdorff distance (87). Six papers only used one single metric. To comparatively evaluate the models according to their performance, we assigned a generalisability score to each of the included studies based on sample representativeness—considering sample size, number of study sites, gender equality, age range, length of the data collection period, number of scanners, external validation performed –, ground-truth data, and access to clean code (Table 6, third column from right to left). Liu et al. (32) and Jeong, et al.'s (79) algorithms were deemed “highly” generalisable, whereas 19 algorithms had “low” generalisability. Plotting the reported performance against the generalisability scores obtained revealed that Dice and generalisability scores were positively correlated (Supplementary Data Sheet 2 S4a).

**Table 6:** Performance and generalisability data (see corresponding summary graphs in the Supplementary Data Sheet 1 B).

First author	Dice	Precision	Recall	Hausdorff distance	Lesion size- based results	General-isability	Train time	Training library and infrastructure
	‐ Only scores reported on test sets are extracted ‐ When scores are reported per input dataset, the average score is provided ‐ Format: mean score ± standard deviation				Results as reported based on lesion size			
Karthik et al. (47)	0.701	–	–	–	N	L	7h30	CPU: 3.6 GHz QuadCore Intel Gen7 RAM: 32GB GPU: Nvidia Quadro P4000 Library: Keras/TensorFlow
Gómez et al. (28)	0.36 ± 0.21	0.42 ± 0.25	0.48 ± 0.29	–	N	L	–	–
Olivier et al. (54)	0.703 ± 0.2	–	–	–	Sensitivity: S (<20 ml): 0.987 L (>=20 ml): 0.923 Specificity: S (<20 ml): 0.923 L (>=20 ml): 0.987	M	–	GPU: Nvidia Tesla K80 Library: Keras/TensorFlow
Clèrigues et al. (55)	0.715 ± 0.205	0.735 ± 0.25	0.745 ± 0.18	27.7 ± 21.45	N	M	–	CPU: Intel CoreTM i7–7800X OS: Ubuntu 18.04 RAM: 64GB GPU: Nvidia Titan × (12GB) Library: Torch
Liu et al. (56)	0.764	–	0.944	3.19	N	M	–	–
Moon et al. (57)	0.737 ± 0.32	0.758	0.755	22.047	Relation dice-lesion size: Observed *R*^2^ = 0.195	L	24 h	Library: O Keras/TensorF loS: Centos7 GPU: 4×Nvidia Quadro RTX 8000 w
Zhang et al. (19)	0.791	0.927	0.782	–	N	M	6h23	CPU: Intel Core i7-4790 3.60 GHz RAM: 16 GB GPU: Nvidia Titan X Library: PyTorch
Wong et al. (52)	0.84 ± 0.03	0.84 ± 0.03	0.89 ± 0.03	–	N	M	–	–
Khezrpour et al. (58)	0.852	0.998	0.856	–	N	L	–	GPU: Google Cloud Compute (K80) Library: Keras/TensorFlow
Hu et al. (59)	0.30 ± 0.22	0.35 ± 0.27	0.43 ± 0.27	-	N	L	–	GPU: 4×Nvidia Titan Xp
Gheibi et al. (42)	0.792	–	–	–	N	M	1h27	GPU: Nvidia Tesla P100 Library: Keras
Kumar et al. (60)	–	0.633 ± 0.213	0.653 ± 0.223	–	N	M	11h45	CPU: 2× Intel Xeon Silver 4114 (2.2 GHz, 10C/20 T) RAM: 192GB GPU: Nvidia Tesla V100 PCIe Library: Keras/TensorFlow
Liu et al. (16)	0.817	–	–	1.92	N	M	0h36	Library: Keras/TensorFlow
Zhao et al. (61)	0.699 ± 0.128	0.852	0.923	–	Dice: S: 0.718 (0.12) L: 0.689 (0.222)	M	–	CPU: Intel Core i7-6800K RAM: 64GB GPU: Nvidia GeForce 1080Ti Library: PyTorch
Liu et al. (32)	0.76 ± 0.16	0.83 ± 0.17	0.73 ± 0.19	–	Dice: S (<1.7 ml): 0.68 (0.19); M (≥1.7 & <14 ml): 0.75 (0.14); L (≥14 ml): 0.83 (0.10)	H	–	CPU: Intel Core E5-2620v4 (2.1 GHz) GPU: 2×Nvidia Titan XP Library: Keras/TensorFlow
Karthik et al. (48)	0.7535	–	–	–	N	L	34h04	CPU: 3.6 GHz QuadCore Intel (Gen 7) RAM: 32GB GPU: Nvidia Quadro P4000 Library: Keras/TensorFlow
Liu et al. (62)	0.68 ± 0.19	–	–	39.975 ± 27.95	N	M	–	–
Aboudi et al. (63)	0.558	0.998	–	–	N	L	–	CPU: Intel Core i5 8th gen RAM: 8GB GPU: Nvidia GeForce GTX 1050 Library: Keras/TensorFlow
Pinto et al. (64)	0.29 ± 0.21	0.23 ± 0.21	0.66 ± 0.29	41.58 ± 22.04	N	L	–	GPU: Nvidia GeForce GTX-1070 Library: Keras/Theano
Choi et al. (65)	0.31	–	–	37.7	N	L	3h	CPU: 2×Intel Xeon CPU E5-2630 v3 (2.4 GHz) GPU: 4× Nvidia GeForce GTX TITANX Library: Keras
Kim et al. (66)	0.6 ± 0.23	–	–	–	Dice:>0.75 for lesion volumes >70 ml	L	20h	CPU: Intel Xeon Processor E5-2680 (14 CPU, 2.4 GHz) OS: Ubuntu Linux 14.04 SP1 RAM: 64GB GPU: Nvidia GeForce GTX 1080 Library: TensorLayer
Woo et al. (30)	0.858 ± 0.0734	–	–	–	Dice: - S (<10 ml): 0.82 - L (>10 ml): 0.89	L	–	–
Lee et al. (31)	0.854 ± 0.008	0.845	0.995	–	N	L	–	–
Lee et al. (67)	0.422 ± 0.277	0.48 ± 0.308	0.467 ± 0.32	–	Dice: S (<10 ml): 0.377 L (>10 ml): 0.607	M	52h30	CPU: Xeon Processor E5-2650 v4 GPU: Nvidia Titan X Library: Keras/TensorFlow
Karthik et al. (68)	0.775	0.751	0.801	–	N	L	–	CPU: 4 cores OS: Ubuntu 16.04 RAM: 32GB GPU: 2×Nvidia Tesla P100 Library: PyTorch
Zhang et al. (69)	0.433	–	0.356	–	N	L	–	GPU: Nvidia GeForce GTX 1080 Ti Library: Keras/TensorFlow
Ou et al. (70)	0.865	0.894	0.818	–	N	M	4h	GPU: 4×Nvidia Quadro RTX 6000 Library: PyTorch
Vupputuri et al. (71)	0.71	–	0.897	–	N	M	–	GPU: Nvidia Tesla K80
Abdmouleh et al. (72)	0.71 ± 0.11	–	–	–	N	L	–	–
Duan et al. (73)	0.677 ± 0.165	–	–	85.462 ± 14.496	N	M	–	GPU: Nvidia GTX 1080 Ti Library: PyTorch
Lucas et al. (74)	0.35	0.52	0.35	21.48	N	L	–	GPU: Nvidia Titan Xp (12GB) Library: PyTorch
Nazari-Farsani et al. (49)	0.5	–	0.6	–	N	M	–	–
Wei et al. (53)	0.828	–	–	–	Dice: S (<769 pixels): 0.761 L (>769): 0.83	M	–	–
Li and Ji (75)	–	–	–	38.27mm	N	L	–	–
Liu et al. (76)	0.658	0.61	0.6	51.04	N	M	–	CPU: Intel Core i7-7700K RAM: 48GB GPU: Nvidia GeForce 1080Ti Library: Keras/TensorFlow
Cornelio et al. (77)	0.34	–	–	–	N	L	5h	OS: Ubuntu v.16.04.3 GPU: Nvidia GeForce GTX Library: Keras/TensorFlow
Yu et al. (50)	0.53	0.53	0.66	–	N	M	35h	GPU: Nvidia Quadro GV100 & Nvidia Tesla V100-PCIE Library: Keras/TensorFlow
Wu et al. (78)	0.856	0.883	0.854	27.34	N	M	0h21	GPU: 6×Nvidia Tesla 4s Library: PyTorch
Guerrero et al. (39)	0.4 ± 0.252	–	–	–	N	L	–	Library: Lasagne/Theano
Jeong et al. (79)	0.787	–	–	–	N	H	–	RAM: 80GB GPU: Nvidia A100 Library: PyTorch
Gui et al. (80)	0.801 ± 001	–	0.783 ± 0.001	3.01 ± 0.03	N	M	–	GPU: Nvidia 3090Ti Library: PyTorch

**Figure 6:**
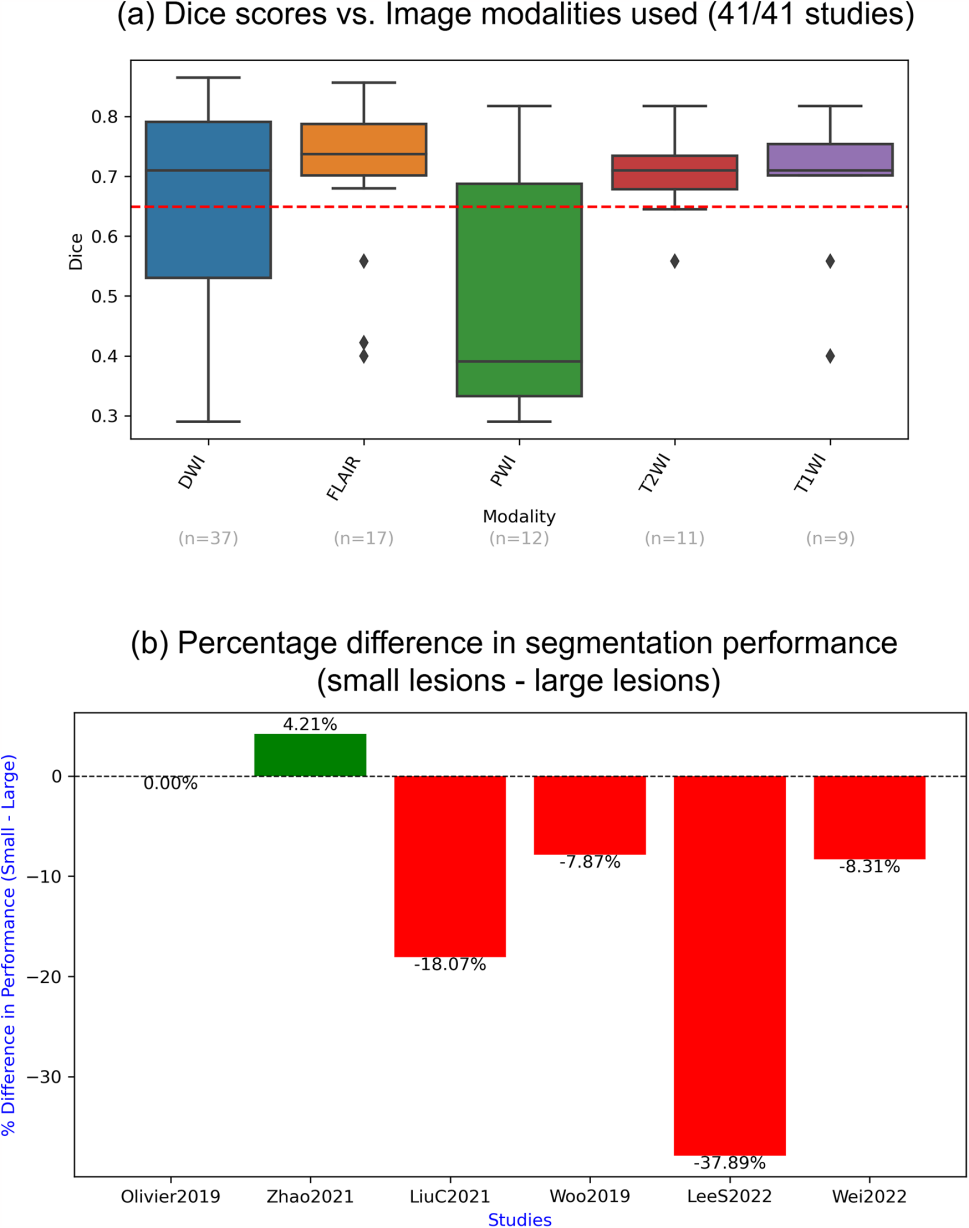
Impact of different MRI modalities on the accuracy of lesion segmentation. **(a)** Box plot showing the correlation between Dice scores and imaging modalities used; **(b)** percentage difference in lesion segmentation performance for small vs. large lesions, calculated as (small lesion performance—large lesion performance) relative to large lesion performance. Positive values indicate better performance on small lesions, while negative values indicate better performance on large lesions.

Only six papers analysed segmentation performance in relation to lesion size (i.e., on small vs. large lesions), and in four of them, accuracy on small lesions was lower or significantly lower (Figure 6b). As shown in Supplementary Data Sheet 2 S4c, lesion volume ranges differed substantially between studies, and all cases with low mean Dice (<0.5) (8 studies) reported low mean lesion volumes (<40 ml), while all cases with higher lesion volumes (>60 ml) (4 studies) reported high Dice scores (>0.68). In other words, segmentation performance was generally better when lesions were larger.

As shown in Figure 6a, Dice scores were above the overall mean and relatively consistent across T2-WI, T1-WI, and FLAIR imaging modalities (mean Dice around 0.7), while PWI exhibited lower-than-average performance (mean Dice 0.38). Only for DWI did all the data points fall within the IQR (between 25^th^ and 75th percentiles), as outliers with below-average Dice scores were observed for FLAIR (three), T1WI (two) and T1WI (one). Additionally, the lower half of the IQR (25th-to-50th percentile) was substantially wider than the upper half (50th-to-75th percentile) for DWI, whereas the opposite pattern appeared in the IQR for PWI.

We also saw a positive correlation between spatial resolution and reported segmentation performance (Supplementary Data Sheet 2 S4c). Nine studies performed external validation of their models on unseen data, and 5/7 studies obtained higher Dice values on their test set than on the external validation set. We also observed a positive correlation between sample size and segmentation performance. Also, single-centre studies showed better performance (mean Dice 0.71) than multi-centre studies (mean Dice 0.6).

Dice scores were much higher for studies using ISLES-2022 (mean Dice >0.8), ISLES-2015 (mean Dice >0.7) or proprietary datasets (mean Dice >0.7), than when using ISLES-2017 (mean Dice 0.38) or DEFUSE (mean Dice 0.52) (Supplementary Data Sheet 2 S5a). When attention-based networks were deeper, or when U-Nets were deeper, Dice scores were higher. The mean Dice was also higher when attention was used (0.71 vs. 0.6 if not used) (Supplementary Data Sheet 2 S4d).

Models using focal loss heavily under-performed, while those using learning rate adjustment over-performed. There was negative correlation between Dice scores and numbers of epochs used. Interestingly, only one of the algorithms that used a relatively high number of epochs was also using early stopping regularisation, which means that for all the others, the full (high) amount of epochs was used during training, hence substantially increasing the probability of overfitting.

### 4.7 Reported dice scores and segmentation quality

**Figure 7:**
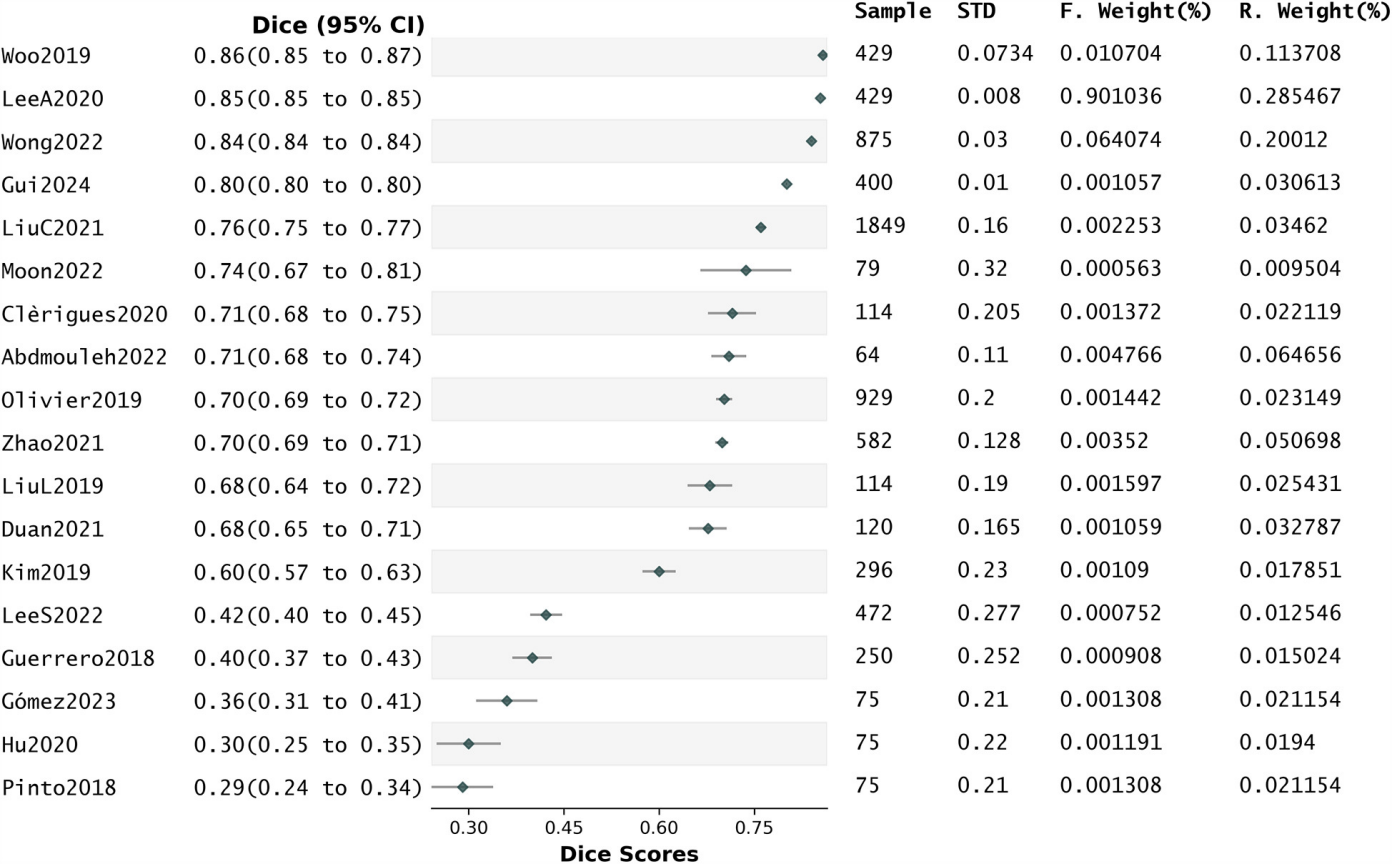
Forest plot related to the whole group analysis.

We explored whether the reported Dice scores are a legitimate indicator of segmentation quality in this review. For this we generated a forest plot using the data from the 18 papers that reported their Dice along with their standard deviation (Figure 7). In this analysis, the percentage of variation across studies due to heterogeneity rather than chance (*I*^2^) was 23.44%.

We also conducted a sensitivity analysis using Precision scores as effects size instead of Dice scores. This analysis involved only eight studies, which reported their precision scores along with standard deviations. But in this analysis, *I*^2^ was 8.49%, indicating a reduced level of heterogeneity between studies, therefore precluding us to derive conclusions from it (Supplementary Data Sheet 2 S6).

Funnel plots and Egger's tests (Supplementary Data Sheet 2 S7, S8) conducted using the Dice scores reported by the included studies indicated the presence of publication bias in favour of studies reporting high values of this metric.

### 4.8 Influence of attention on dice scores

**Figure 8:**
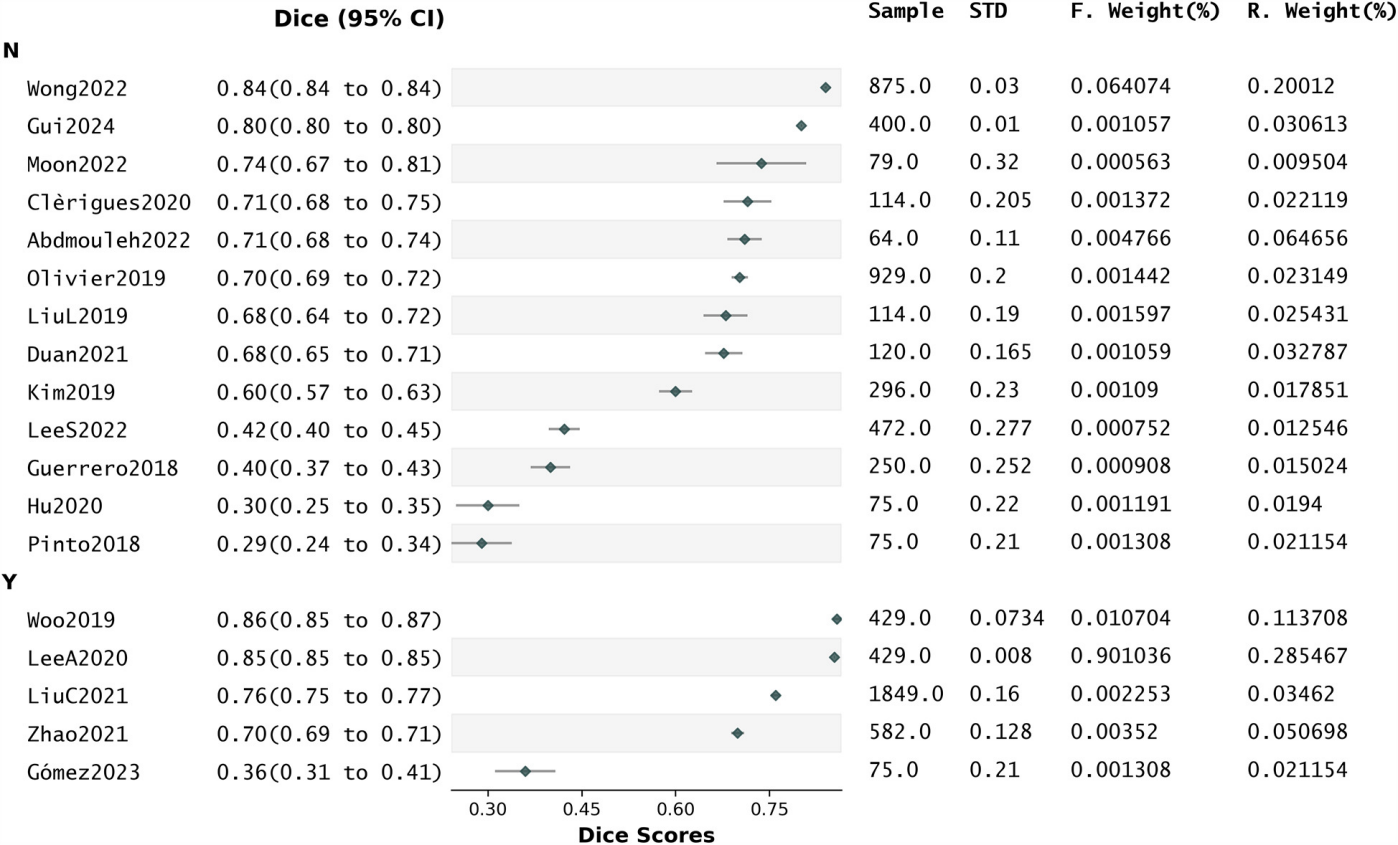
Forest plot related to the subgroup analysis.

We conducted a subgroup analysis to evaluate the association between attention mechanisms and Dice scores. The resulting forest plot is shown in Figure 8.

There were no statistically significant differences in effect sizes between the groups. The subgroup “with attention” indicated moderate heterogeneity in I^2^ (31.63%) and a very high Z-stat (39.03, *p* < 0.001), suggesting a substantially large overall effect. While this implies that the presence of attention may enhance segmentation performance, the small number of studies in this subgroup (five) limits the conclusiveness of this result. In contrast, the subgroup “without attention” comprised 13 studies, showing significant heterogeneity in the Q-stat (Q = 20.06, *p* = 0.07) and in I^2^ (35.20%). Despite the absence of attention, a large overall effect was also observed (z = 3.13, *p* < 0.001). This suggests that when attention is not used, the Dice scores differ between studies.

Further meta-regression analysis to assess the statistical significance of the relationship between “attention mechanisms” and “Dice scores” (Supplementary Data Sheet 2 S9) revealed that 8.1% of the variance in Dice scores was explained by the presence of attention (R-squared: 0.081), but the slope indicating the change in Dice associated with the presence of attention was not statistically significant [0.117, *p* = 0.27, 95% CI of the slope (−0.100,0.334)]. This indicates that from the literature analysis we cannot conclude that the presence of attention has a significant impact on the likelihood of high Dice.

### 4.9 Risk of bias assessment

After assessing the possibility of biases in the included studies, 33 studies scored “GOOD”, and eight scored “FAIR” in the NIH study QA (Supplementary Data Sheet 1 C). Although these results are positive, we identified cases of potential *spectrum bias* (88), mostly due to the following factors: acute stroke studies were more represented than subacute (30 vs. 18), exposure was often only assessed once (i.e., no follow-up scans) (26 studies), variance and effect estimates were not both provided (23 studies), few experiments were conducted to assess the different levels of exposure related to the outcome (11 studies), period of data collection was relatively short (10 studies), study population was poorly defined (3 studies), and the age range of participants was not always consistent [e.g., Kim et al. (66) only included patients between 58 and 79 years old].

We also noticed cases of *selection bias*. Multiple studies used the same ISLES datasets to evaluate the performance of their segmentation methods. This, although advantageous (e.g., cost effective, allows comparability), introduces selection bias. These were also studies where males were over-represented in the sample.

Also, ground-truth data were most often obtained by manually refining semi-automatic segmentations (e.g., thresholding followed by region-growing), which introduces *observer bias*. Sixteen studies did not provide information about labelling criteria, so it is unclear whether observer bias was present in those.

We identified two other forms of bias: *verification bias* in 10 studies, where only one expert did the labelling of ground-truth images, and *measurement bias*, as mean Dice scores on ISLES-2017 were generally much lower than those on ISLES-2015 or on ISLES-2022, and when segmentation performance was reported for small vs. large lesions, the definition of a small and a large lesion (in ml) was not consistent across studies.

### 4.10 Pilot analysis

The best performing model was “UResNet50” on DWI (single-modality approach), using a weighted compound loss (BCE = 0.3 + Dice = 0.7), with a Dice score on the validation set of 0.692 ± 0.132 (Table 7).

**Table 7:** Results from the pilot analysis.

UResNet50	Mean dice score (± STD)			
	DWI	DWI	DWI + FLAIR + T1WI + T2WI	DWI + FLAIR + T1WI + T2WI
UResNet50	Train	Validation	Train	Validation
BCE = 0.3 + Dice = 0.7	0.911 ± 0.11	0.692 ± 0.132	0.908 ± 0.041	0.675 ± 0.128
BCE = 0.5 + Dice = 0.5	0.893 ± 0.102	0.610 ± 0.055	0.884 ± 0.318	0.619 ± 0.301
BCE = 0 + Dice = 1	0.902 ± 0.205	0.625 ± 0.306	0.886 ± 0.16	0.608 ± 0.04
UNet	Train	Validation	Train	Validation
BCE = 0.3 + Dice = 0.7	0.843 ± 0.322	0.556 ± 0.083	0.838 ± 0.072	0.570 ± 0.159
BCE = 0.5 + Dice = 0.5	0.829 ± 0.031	0.521 ± 0.29	0.836 ± 0.2	0.547 ± 0.234
BCE = 0 + Dice = 1	0.837 ± 0.202	0.560 ± 0.105	0.842 ± 0.085	0.555 ± 0.18
AG-UResNet50	Train	Validation	Train	Validation
BCE = 0.3 + Dice = 0.7	0.907 ± 0.121	0.676 ± 0.222	0.909 ± 0.177	0.664 ± 0.313
BCE = 0.5 + Dice = 0.5	0.899 ± 0.06	0.642 ± 0.176	0.873 ± 0.096	0.630 ± 0.269
BCE = 0 + Dice = 1	0.893 ± 0.19	0.669 ± 0.091	0.877 ± 0.231	0.631 ± 0.164
AG-UNet	Train	Validation	Train	Validation
BCE = 0.3 + Dice = 0.7	0.829 ± 0.258	0.522 ± 0.142	0.817 ± 0.109	0.536 ± 0.22
BCE = 0.5 + Dice = 0.5	0.793 ± 0.2	0.518 ± 0.207	0.802 ± 0.163	0.515 ± 0.082
BCE = 0 + Dice = 1	0.797 ± 0.32	0.529 ± 0.099	0.784 ± 0.27	0.498 ± 0.105

The second best was “AG-UResNet50” (0.676 ± 0.222), with a single-modality approach, and using the same compound loss (BCE = 0.3 + Dice = 0.7).

Experiments with “UNet” and “AG-UNet” generated relatively poor Dice scores. Performance was better in single-modality experiments. Abdmouleh et al. (72) made the same test on the same dataset, but they achieved quasi-equal performance in their DWI-only and multi-modal experiments (Dice 0.71). Performance was also better when using compound loss “BCE = 0.3 + Dice = 0.7” vs. the other two types. The 12 experiments using attention and the 12 not using attention yielded similar average Dice scores.

Average training times for UResNet50 was 5 h 43 min, for U-Net it was 5 h 31 min, for AG-UResNet50 it was 6 h 15 min, and for AG-UNet it was 5 h 55 min. Multi-modal experiments took longer to train in all cases (∼3 h longer each time). Same was true for attention-based experiments (∼30 min longer each time).

## 5 Discussion

### 5.1 Systematic review and meta-analysis

We performed a comprehensive systematic search in seven large databases for sources presenting algorithms that identify and segment acute and subacute ischaemic stroke lesions from brain MRI, to inform on the most promising DL architectures for successfully carry out this task. From 1,485 initially identified sources, 41 were ultimately retained. Their analyses allowed us to conclude that the use of a U-Net configuration with residual connections seems to be the most appropriate configuration for this task, despite the generalisability of the algorithms reviewed being generally below par.

#### 5.1.1 Sample representativeness

Although our review protocol did not have age restriction, samples never included patients below 18 years old. This stresses the lack of research in paediatric stroke, which may be due to multiple factors, e.g., delayed identification of stroke, numerous stroke aetiologies and risk factors in children, and limited imaging data (89). The underrepresentation of females in studies can be partially explained by the difficulty of diagnosing females with stroke, due to factors such as higher proportion of stroke mimics (e.g., migraine), pre-stroke disability, or neglect of symptoms among females (90). These uneven distributions of gender and age data can affect the universality of our research outcomes.

We also noticed relatively small sample sizes across studies, which is not new in AIS research (91). Data augmentation is a common way to mitigate this issue, and Clèrigues et al. (55) proposed a novel “symmetric modality augmentation” technique, which leveraged learned features based on the symmetry of brain hemispheres. Other ways to deal with small sample sizes include active learning [e.g., Olivier et al. (54)], semi-supervised learning using weakly labelled data [e.g., Zhao et al. (61)], or transfer learning [e.g., Li et al. (75) used TernausNet (92) which was pre-trained on ImageNet (93), and Jeong et al. (79) used an ensemble of nnU-Nets which were pre-trained on BraTS 2021 (94)].

#### 5.1.2 Disease representativeness

Most studies focused exclusively on minor-to-moderate stroke cases with focus on acute stroke, since DWI and FLAIR are able to show high signal in AIS-affected brain areas, whereas signal begins to diminish gradually in DWI towards the subacute stage, often leading to lower sensitivity for stroke identification if this modality is used (4). Such differences in MRI signal between subacute and acute lesions give the idea that combining acute and subacute cases in one single dataset, as seen in Liu et al. (32) and Liu et al. (76), might require highly trained observers to manually delineate the lesions (i.e., generate the reference labels).

#### 5.1.3 MRI protocols

Most studies used DWI, known as the gold standard for early stroke detection (95), and many used T1-WI, a staple in subacute stroke research (96), T2-WI, PWI, or FLAIR. PWI was frequently applied to detect the ischaemic penumbra (86), and most used FLAIR as it offers enhanced lesion clarity by suppressing CSF details (97). For instance, Khezrpour et al.'s U-Net used only FLAIR and got very high accuracy (58). ADC maps were also often used with DWI for more robust ground-truth data, as lesions appear simultaneously hyperintense on DWI and hypointense on ADC in early stroke stages.

The impact of using different imaging modalities (i.e., T1-WI, T2-WI, DWI, PWI, FLAIR) on lesion segmentation accuracy was also observed, as each modality may highlight distinct pathological features, which may, in turn, influence algorithm performance. More generally, using 3 T magnetic field strength, as done by 36/41 studies, can also help with small lesions, as it offers better signal-to-noise ratio and spatial resolution vs. 1.5 T, and it reduces imaging artifacts by offering more uniform B1 inhomogeneity (98).

DWI-PWI mismatch (99) was commonly used to create ground-truth sets [e.g., Lee S. et al. (67)], since PWI identifies penumbral tissue, while DWI delineates the core infarct [i.e., areas of restricted water diffusion (96)]. Despite its utility though, DWI-PWI mismatch analysis remains challenging. Establishing clear imaging boundaries for recoverable tissue is not straight-forward (96). Large perfusion abnormalities may be observed in patients without corresponding clinical deficits (100). There is no universally defined mismatch ratio, although Kakuda et al. tried to define one (101) DWI-FLAIR mismatch, on the other hand, is mostly used for TSS assessment in hyper-acute-to-early-acute stage (102). Combining both mismatch analyses can definitely help experts effectively delineate stroke lesions.

#### 5.1.4 Data configurations

Many argue that using 3D images is crucial for DL-based stroke lesion segmentation, but few methods address the associated computational challenges (103), which explains why the majority of retained studies used 2D images.

Several studies used high spatial resolution images to capture more fine-grained features from the data and improve segmentation performance on small lesions. Other deepened their networks further to collect more nuanced features, but the higher the number of down-sampling operations, the lower the resolution of the feature maps, to a point where reconstructing lesions in the up-sampling path becomes virtually impossible. Furthermore, risks of overfitting/over-learning increase substantially when networks are deeper, especially in absence of skip connections.

Cutting 3D images into 3D patches (i.e., patch-wise training) is a way to mitigate both the computational challenges, by reducing memory overhead (13), and the small lesions challenge, by forcing the model to focus on a smaller area of the entire image. That explains why ten out of twelve 3D studies in this review have used patch-wise training.

On the other hand, the majority of studies that used ISLES-2015/2017 have processed those as 2D images, mainly due to their low-resolution when processed as 3D (slice thickness: 5 mm). However, it was surprising to see so many 3D models use low resolution images, since the whole point of 3D models is to capture detailed information from images (104). For instance, Zhang R. et al. (19) proposed a 3D model that captured both low-level local features and high-level ones, but they used low-resolution images.

#### 5.1.5 Validation metrics

Dice was the most used performance metric across studies, as (i) it is simple to interpret, (ii) it handles class imbalance, and (iii) its widespread use facilitates comparison between different methods. However, it remains an overlap metric that is prone to instability, especially with small lesions (78), and for an evaluation to be holistic, it must be accompanied by other types of metrics (e.g., surface-based, boundary-based, volume-based). Dice scores were higher for single-centre studies, but since too few of these studies performed external validation, we cannot exclude “over-adaptation” to the image acquisition protocol(s) from that one centre, and therefore poor model generalisability.

#### 5.1.6 Loss functions

CE loss quantifies the difference between two probability distributions (e.g., predictions and ground-truth), but it cannot handle class imbalance since each pixel/voxel contributes equally to the loss, and therefore the learning process may easily fall into a local optimal solution (105). Focal loss is an adaptation of CE loss that introduces a modulating factor aimed at down-weighting the impact of well-classified examples (106), but since “lesion” is already the minority class in our case, focal loss overly penalizes correctly classified lesion pixels, which explains the very bad performance of studies using it [e.g., Hu et al.'s Brain SegNet (59)].

Generally, overlap-based loss functions (e.g., Dice loss) are more robust to data imbalance issues (106). By penalising false positives and false negatives differently, Dice loss indirectly encourages better performance on minority classes. However, despite its common usage, Dice loss has some limitations (106): it fails to capture the distance between non-overlapping but close lesions, overlooks precise contour details (combining it with a boundary-based loss may help), and it disproportionately penalises small lesions, especially in presence of large lesions, as opposed to distribution-based loss functions (e.g., CE loss) which have no such bias. A few custom loss functions have also been proposed to address class imbalance [e.g., Rachmadi et al.'s “ICI loss” (107), loss with data fusion (108)].

#### 5.1.7 Deep learning architectures

Since most studies were U-Net-based, they primarily performed semantic lesion segmentation. Perhaps the fact that only two studies did instance segmentation is linked to the difficulty of delineating individual lesions in presence of motion artefacts and irregular shapes (109, 110), as shown by Wu et al. (78).

Meanwhile, several studies proposed quite innovative methods. Liu et al. (76) proposed a ResNet and a global convolution network-based (GCN) encoder-decoder where each modality was concatenated to a three-channel image, then passed as input image to a series of residual blocks. The output of each block was then passed to its corresponding up-sampling layer using a skip connection incorporating a GCN and a boundary refinement layer. Liu L. et al.'s “MK-DCNN” (62) consisted of two sub-DenseNets with different convolution kernels, aiming to extract more image features than with a single kernel by combining low and high resolution. Four studies proposed “ensemble mechanisms” (i.e., different networks that process data inputs in parallel and whose outputs are combined) in order to reduce overfitting, since sub-networks can learn different features from the data (13) and/or to decrease prediction variance [e.g., Choi et al. (65)]. Wu et al.'s W-Net (78) tackled variability in lesion shape by trying to capture both local and global features in input scans. A U-Net first captures local features, which then go through a Boundary Deformation Module, then finally through a Boundary Constraint Module that uses dilated convolution to ensure pixels neglected in previous layers can also contribute to the final segmentation. Pinto at al. (64), Duan et al. (73) and Zhang et al. (69) proposed “information fusion mechanisms” that effectively fuse different features either from multiple modalities, or multiple plane views, thus improving their models' ability to capture intricate lesion features. Jeong et al. (79) implemented a hybrid image fusion approach in their multimodal study, using all modalities during training to leverage complementary features, while relying solely on DWI images for inference to mitigate overfitting and enhance generalizability. Lucas et al. (74) added to their U-Net skip connections around each convolution block, besides those linking encoder-decoder layers.

The nnU-Net is particularly useful as it automates complex and rapidly evolving stages of the pipeline—data pre-processing, network configuration, optimization, regularization, and data post-processing (20). The nnU-Net has demonstrated strong generalizability (79), partly due to its standardized pipelines, its multiple regularization techniques, and a balanced network depth that helps reduce overfitting. Its success leverages the modular nature of U-Net architectures, but it remains relatively rigid; it does not natively support architectural enhancements like residual or attention or transformer blocks, custom loss functions, or late/hybrid fusion strategies for multimodal data, all of which have shown potential to further improve segmentation performance.

#### 5.1.8 Attention mechanisms

The main purpose of attention mechanisms is to address the loss of information during down-sampling and up-sampling operations. Self-attention was often used across studies, since it allows the model to capture global dependencies within the input data, which can help in identifying subtle features that span across larger regions.

Overall, there were several interesting implementations, or pseudo-implementations, of attention. Karthik et al. (68) embedded multi-residual attention blocks in their U-Net, hence allowing the network to use auxiliary contextual features to strengthen gradient flow between blocks and prevent vanishing gradient issues. Vupputuri et al. (71) used self-attention through multi-path convolution, aiming to compensate for information loss, while using weighted average across filters to provide more optimal attention-enabled feature maps. Ou et al. (70) used lambda layers, which work by transforming intra-slice and inter-slice context around a pixel into linear functions (or “lambdas”), which are then applied to the pixel to produce enhanced features. As opposed to attention, lambdas do not give “weights” to pixels. We believe that it is only a coincidence that ResNet-based models never incorporated attention across reviewed studies, as numerous relevant publications combine ResNet with attention (111–113).

#### 5.1.9 Optimization methods

In terms of optimisation methods, RMSProp can be effective in DL [e.g., Ou et al. (70)], as it is able to discard history from the extreme past and thus enable rapid convergence during training. However, Adam remains the most popular method as it incorporates momentum, which speeds up the optimisation of model parameters, while performing bias corrections to improve the accuracy of gradient estimates during training. Also, Adam's default hyperparameters often work well in DL, mainly thanks to the adaptive learning rates which allow smooth parameter updates even in presence of noisy gradients.

While never performed, uncertainty quantification to obtain true network uncertainty estimates (88) is of utmost importance to promote the use of such algorithms in clinical practice, as it would allow physicians to assess when the network is giving unreliable predictions (6).

#### 5.1.10 Generalisability & sources of bias in retained studies

The generalisability of our studies was generally low, for issues that have already been highlighted above (e.g., small sample sizes, loose verification of labelled data), but researchers can easily improve the generalisability of their models by performing external validation, publishing their code, combining image acquisition protocols, and/or combining data from multiple centres.

Our risk of bias assessment yielded fairly good results. However, several instances of potential or actual bias warrant attention. Findings drawn from reported performance metrics (e.g., Dice) must be carefully interpreted, as performance depends on the quality of the data being used, which was variable across studies. Results of this review may be skewed towards acute stroke (rather than subacute), which impacts the applicability of its results and recommendations in stroke research and clinical practice. Over-reliance on specific public datasets, which may have selection biases, may limit the generalisability of the research findings, as reported results may not fully represent all possible clinical scenarios. Findings in terms of segmentation of small vs. large lesions are slightly flawed, due to the various ways in which these two categories were defined across studies. Data augmentation helped reduce overfitting by increasing the size of the training data, but effects of bias cannot be balanced-out by increasing the sample size by repetition (114). Differences in expert annotation policies, commonly referred to as inter-observer (dis)agreement, were identified as a source of selection bias. Unsupervised or semi-supervised methods could mitigate this issue. Reframing the segmentation task as an in-context learning task where the model is prompted with a small number of example segmentations from a previously unseen policy at inference time could also be a solution, but this is still to be tested. Ensembles of different networks have proven effective for different tasks, and could be, in fact, the best approach to tackle this issue.

#### 5.1.11 Meta-analyses

Our whole group analysis included 18 studies, which is enough to consider findings meaningful (115). The random-effects model worked better for us, which is aligned with the literature, where RE is considered a more natural choice than FE in medical research (116). The most interesting finding resulted from the subgroup analysis. It is the uncertainty in the evidence that incorporating attention into DL architecture for AIS lesion segmentation improves model performance.

Meanwhile, the significant heterogeneity observed through these analyses may be linked to several factors, such as differences in image acquisition protocols (e.g., spatial resolution, scanners), patient populations (e.g., stroke stage, severity, aetiology), network architecture (e.g., U-Net, ResNet), model hyper-parameters, and more. Therefore, when looking into ways to improve DL-based stroke lesion segmentation algorithms, our analysis suggests that one might want to look at factors other than attention (e.g., image quality, model architecture and complexity).

### 5.2 Pilot analysis

The relatively high Dice scores obtained on training sets vs. validation sets are likely caused by overfitting, partly due to the small sample size, despite efforts to mitigate this with data augmentation and pixel dropout.

We used the ISLES-2015-SISS dataset for this analysis. It is worthwhile noting that it may not sufficiently capture the variability across different populations and lesion types, and the limited sample diversity could limit the generalizability of the model across different demographics or lesion types. However, from the 39 publications analyzed, only 12 used this sample in the development of their proposed algorithm sometimes as part of a wider sample (5/12 publications) (Table 4). In terms of number of 3D volumes the sample is small, but we use a 2D model for which the number of input samples with image information multiplies the available data sources by a factor of approximately 100 considering only one dimension (e.g., considering horizontal-only or sagittal-only or coronal-only slices), but if slices in the three main imaging axes are considered, then the increase is three times that.

Not using attention yielded slightly better than using it. In this case, with a small sample size and a relatively deep network, increasing the number of learnable parameters using attention gates might have accentuated the overfitting problem. Complementing our analysis with additional performance metrics (e.g., HD, Accuracy, Precision) could further support this observation.

The fact that the single-modality approach (DWI-based) performed better than the multi-modal approach is counter-intuitive, since combining sequences has often led to an improved segmentation performance, as shown by Liu et al. (16) and Liu et al. (76), who did the same comparison of approaches. However, it could be that specifically in the ISLES-2015-SISS dataset, the mix of image acquisition protocols across centres, sequences' mismatches, and annotation policies have introduced noise in the data, which was not properly removed during data pre-processing or managed by the networks (86).

Compound loss (Dice + CE) outperformed Dice loss, as it was the case with Kumar et al.'s “CSNet” (60). Since Dice loss is not suitable for small diffuse lesions, combining distribution-based loss with region-based loss has certainly helped.

UResNet50 addresses the challenge of distinguishing stroke lesions from other pathologies, which can vary by stage. Its effectiveness confers it potential to improve diagnostic accuracy and treatment planning for stroke patients, ultimately contributing to better clinical outcomes.

## 6 Study limitations

This review has various limitations. Only articles published in (or translated to) English that were accessible via institutional login were reviewed. Accordingly, relevant papers may have been missed. Incongruences between search terms and article keywords in the various databases may have also caused relevant articles to be missed. Since most of the included studies were not longitudinal, this review lacks an assessment of long-term patient outcomes, which is an essential factor in validating the clinical relevance and predictive value of segmentation algorithms. While the review outlines the impact of lesion size on segmentation performance, the pilot analysis does not specifically assess how algorithms can be optimized for lesions of varying sizes.

## 7 Conclusions and future works

While we included a fair number of studies in this review, the identified generalisability issues hinder the robustness of our findings. However, we were able to (i) identify the often subtle elements and configurations that can make a DL model perform better its AIS lesion segmentation task, and to (ii) demonstrate with confidence that attention mechanisms do not necessarily improve current DL architectures for AIS semantic lesion segmentation, and that other details such as model design were much more important.

We have compared multiple model artefacts (e.g., loss functions, optimisation methods), discussing their potential impacts on segmentation performance. A more formal decision tree could complement our research, helping to (i) facilitate decision-making during model development, and (ii) enhance model transparency and trustworthiness in clinical settings.

In this review, algorithms were assessed solely based on performance (using Dice coefficients). A more comprehensive evaluation of their practical value could be conducted in future work by considering other metrics or a combination of them (117), and factors such as processing time, and resource consumption.

More generally, further well-conducted and well-reported research is needed in this field to accelerate their use in routine clinical practice, with special emphasis on: (i) larger datasets, potentially by leveraging consortia such as the Human Connectome Project (https://www.humanconnectome.org/) or ENIGMA (https://enigma.ini.usc.edu/), or curating and fully anonymising large nationwide data from national health services, (ii) higher-quality data, such as generating structured labels from radiologist reports (118), and (iii) longitudinal data to better assess how segmentation results impact patient treatment and prognosis.

Interpretability of algorithms must also improve, as today, computer scientists focus primarily on reaching higher levels of accuracy, while clinical researchers focus on verifying associations with patient outcomes (119). For instance, deconvolution networks and guided back-propagation can explain the inner workings of DL networks (120, 121).

Also, model fine-tuning remains time-consuming. Perhaps “Neural Architecture Search” will soon be a robust solution for automatic selection and parameterization of DL models (122).

At last, following the big leap DL took with the advent of GPU, many scientists are getting prepared for the next big leap, with quantum computing. Although this review did not focus on such technological advancements, the application of quantum algorithmic principles (e.g., running quantum operations on qubits) to ML has already begun (123), and expertise is being built for when quantum hardware will be commercially available. This may increase computing speed significantly.

## Data Availability

The datasets presented in this study can be found in online repositories. The names of the repository/repositories and accession number(s) can be found in the article/Supplementary Material.

## Supplementary material

Supplementary Data Sheet 1A

Supplementary Data A.

Supplementary Data Sheet 1B

Supplementary Data B.

Supplementary Data Sheet 1C Supplementary Data C.

Supplementary Data Sheet 2 Supplementary Figures.

## References

[1] Tsao CW Aday AW Almarzooq ZI Anderson CAM Arora P Avery CL Heart disease and stroke statistics—2023 update: a report from the American Heart Association. Circulation. (2023) 147(8):431–68. 10.1161/CIR.0000000000001123 PMC12135016 36695182

[2] Saka O McGuire A Wolfe C. Cost of stroke in the United Kingdom. Age Ageing. (2008) 38(1):27–32. 10.1093/ageing/afn281 19141506

[3] Zhou Y Huang W Dong P Xia Y Wang S. D-UNet: a dimension-fusion U shape network for chronic stroke lesion segmentation. IEEE/ACM Trans Comput Biol Bioinform. (2021) 18(3):940–50. 10.1109/TCBB.2019.2939522 31502985

[4] Hernandez Petzsche MR de la Rosa E Hanning U Wiest R Valenzuela W Reyes M ISLES 2022: a multi-center magnetic resonance imaging stroke lesion segmentation dataset. Sci Data. (2022). 9(1):762. 10.1038/s41597-022-01875-5 36496501 PMC9741583

[5] Lo EH. A new penumbra: transitioning from injury into repair after stroke. Nat Med. (2008) 14(5):497–500. 10.1038/nm1735 18463660

[6] Litjens G Kooi T Bejnordi BE Setio AAA Ciompi F Ghafoorian M A survey on deep learning in medical image analysis. Med Image Anal. (2017) 42:60–88. 10.1016/j.media.2017.07.005 28778026

[7] Caceres P. Introduction to Neural Network Models of Cognition (NNMOC). (2020). Available at: https://com-cog-book.github.io/com-cog-book/features/cov-net.html (Accessed February 15, 2024).

[8] Goodfellow I Bengio Y Courville A. Deep Learning. Cambridge, MA: MIT Press (2016). Available at: http://www.deeplearningbook.org (Accessed February 15, 2024).

[9] Abang Isa AMAA Kipli K Mahmood MH Jobli AT Sahari SK Muhammad MS A review of MRI acute ischemic stroke lesion segmentation. Int J Integr Eng. (2020) 12(6):3–7. 10.30880/ijie.2020.12.06.014

[10] Hesamian MH Jia W He X Kennedy P. Deep learning techniques for medical image segmentation: achievements and challenges. J Digit Imaging. (2019) 32(4):582–96. 10.1007/s10278-019-00227-x 31144149 PMC6646484

[11] Ronneberger O Fischer P Brox T. U-Net: convolutional networks for biomedical image segmentation. In: Wells WM Frangi AF Navab N Hornegger J, editors. Medical Image Computing and Computer-Assisted Intervention—MICCAI 2015. Cham: Springer International Publishing (2015). p. 234–41.

[12] Lundervold AS Lundervold A. An overview of deep learning in medical imaging focusing on MRI. Zeitschrift Für Medizinische Physik. (2019) 29(2):102–27. 10.1016/j.zemedi.2018.11.002 30553609

[13] Liu L Cheng J Quan Q Wu F-X Wang Y-P Wang J. A survey on U-shaped networks in medical image segmentations. Neurocomputing. (2020) 409:244–58. 10.1016/j.neucom.2020.05.070

[14] He K Zhang X Ren S Sun J. Deep residual learning for image recognition. 2016 IEEE Conference on Computer Vision and Pattern Recognition (CVPR) (2016). p. 770–8

[15] Surekha Y Koteswara Rao K Lalitha Kumari G Ramesh Babu N Saroja Y. Empirical investigations to object detection in video using ResNet-AN implementation method. J Theor Appl Inform Technol. (2022) 100(10):3432–4.

[16] Liu L Chen S Zhang F Wu F-X Pan Y Wang J. Deep convolutional neural network for automatically segmenting acute ischemic stroke lesion in multi-modality MRI. Neural Comput Appl. (2020) 32(11):6545–58. 10.1007/s00521-019-04096-x

[17] Veit A Wilber MJ Belongie S. Residual networks behave like ensembles of relatively shallow networks. *arXiv* [preprint]. *arXiv:1605.06431* (2016). Available at: 10.48550/arXiv.1605.06431 (Accessed February 15, 2024).

[18] Milletari F Navab N Ahmadi S-A. V-Net: fully convolutional neural networks for volumetric medical image segmentation. 2016 Fourth International Conference on 3D Vision (3DV) (2016). p. 565–71

[19] Zhang R Zhao L Lou W Abrigo JM Mok VCT Chu WCW Automatic segmentation of acute ischemic stroke from DWI using 3-D fully convolutional DenseNets. IEEE Trans Med Imaging. (2018) 37(9):2149–60. 10.1109/TMI.2018.2821244 29994088

[20] Isensee F Jaeger PF Kohl SAA Petersen J Maier-Hein KH. nnU-Net: a self-configuring method for deep learning-based biomedical image segmentation. Nat Methods. (2021) 18(2):203–11. 10.1038/s41592-020-01008-z 33288961

[21] Diganta M. Attention mechanisms in computer vision: CBAM. DigitalOcean. (2024). Available at: https://www.digitalocean.com/community/tutorials/attention-mechanisms-in-computer-vision-cbam (Accessed February 15, 2024).

[22] Schlemper J Oktay O Schaap M Heinrich M Kainz B Glocker B Attention gated networks: learning to leverage salient regions in medical images. Med Image Anal. (2019) 53:197–207. 10.1016/j.media.2019.01.012 30802813 PMC7610718

[23] Zheng Z Wang Y Huang Y Song S Yang M Tang B Attention heads of large language models. Patterns (N Y). (2025) 6(2):101176. 10.1016/j.patter.2025.101176 40041856 PMC11873009

[24] Chen X Wang X Zhang K Fung KM Thai TC Moore K Recent advances and clinical applications of deep learning in medical image analysis. Med Image Anal. (2022) 79:102444. 10.1016/j.media.2022.102444 35472844 PMC9156578

[25] Zhao Y Wang X Che T Bao G Li S. Multi-task deep learning for medical image computing and analysis: a review. Comput Biol Med. (2023) 153:106496. 10.1016/j.compbiomed.2022.106496 36634599

[26] Kim J Lee S Hwang E Ryu KS Jeong H Lee JW Limitations of deep learning attention mechanisms in clinical research: empirical case study based on the Korean diabetic disease setting. J Med Internet Res. (2020) 22(12):e18418. 10.2196/18418 33325832 PMC7773508

[27] Takyar A. Attention mechanism. LeewayHertz - AI Development Company Blog. (2021). Available at: https://www.leewayhertz.com/Attention-Mechanism/ (Accessed February 15, 2024).

[28] Gómez S Mantilla D Rangel E Ortiz A Vera DD Martínez F. A deep supervised cross-attention strategy for ischemic stroke segmentation in MRI studies. Biomed Phys Eng Express. (2023) 9(3):035026–6. 10.1088/2057-1976/acc853 36988115

[29] Hu J Shen L Albanie S Sun G Wu E. Squeeze-and-excitation networks. *arXiv* [preprint]. *arXiv:1709.01507* (2017). Available at: 10.48550/arXiv.1709.01507 (Accessed December 01, 2023). 10.1109/TPAMI.2019.2913372 31034408

[30] Woo I Lee A Jung SC Lee H Kim N Cho SJ Fully automatic segmentation of acute ischemic lesions on diffusion-weighted imaging using convolutional neural networks: comparison with conventional algorithms. Korean J Radiol. (2019) 20(8):1275. 10.3348/kjr.2018.0615 31339015 PMC6658883

[31] Lee A Woo I Kang D-W Jung SC Lee H Kim N. Fully automated segmentation on brain ischemic and white matter hyperintensities lesions using semantic segmentation networks with squeeze-and-excitation blocks in MRI. Inform Med Unlocked. (2020) 21:100440. 10.1016/j.imu.2020.100440

[32] Liu C-F Hsu J Xu X Ramachandran S Wang V Miller MI Deep learning-based detection and segmentation of diffusion abnormalities in acute ischemic stroke. Commun Med. (2021) 1(1):61. 10.1038/s43856-021-00062-8 35602200 PMC9053217

[33] Page MJ McKenzie JE Bossuyt PM Boutron I Hoffmann TC Mulrow CD The PRISMA 2020 statement: an updated guideline for reporting systematic reviews. Br Med J. (2021) 372(71):2–6. 10.1136/bmj.n71 PMC8008539 33781348

[34] Linares-Espinós E Hernández V Domínguez-Escrig JL Fernández-Pello S Hevia V Mayor J Methodology of a systematic review. Actas Urol Esp (Engl Ed). (2018) 42(8):499–506. 10.1016/j.acuroe.2018.07.002 29731270

[35] Maier O Menze BH von der Gablentz J Häni L Heinrich MP Liebrand M ISLES 2015—a public evaluation benchmark for ischemic stroke lesion segmentation from multispectral MRI. Med Image Anal. (2017) 35:250–69. 10.1016/j.media.2016.07.009 27475911 PMC5099118

[36] Chen L Bentley P Mori K Misawa K Fujiwara M Rueckert D. DRINet for medical image segmentation. IEEE Trans Med Imaging. (2018) 37(11):2453–62. 10.1109/TMI.2018.2835303 29993738

[37] Alom MZ Hasan M Yakopcic C Taha TM Asari VK. Recurrent residual convolutional neural network based on U-Net (R2U-Net) for medical image segmentation. arXiv [preprint]. *arXiv:1802.06955* (2018). Available at: 10.48550/arXiv.1802.06955 (Accessed February 15, 2024).

[38] Chen H Dou Q Yu L Qin J Heng P-A. Voxresnet: deep voxelwise residual networks for brain segmentation from 3D MR images. NeuroImage. (2018) 170:446–55. 10.1016/j.neuroimage.2017.04.041 28445774

[39] Guerrero R Qin C Oktay O Bowles C Chen L Joules R White matter hyperintensity and stroke lesion segmentation and differentiation using convolutional neural networks. NeuroImage Clin. (2018) 17:918–34. 10.1016/j.nicl.2017.12.022 29527496 PMC5842732

[40] Drozdzal M Chartrand G Vorontsov E Shakeri M di Jorio L Tang A Learning normalized inputs for iterative estimation in medical image segmentation. Med Image Anal. (2018) 44:1–13. 10.1016/j.media.2017.11.005 29169029

[41] Jin Q Meng Z Sun C Cui H Su R. RA-UNet: a hybrid deep attention-aware network to extract liver and tumor in CT scans. Front Bioeng Biotechnol. (2020) 8:41–2. 10.3389/fbioe.2020.605132 33425871 PMC7785874

[42] Gheibi Y Shirini K Razavi SN Farhoudi M Samad-Soltani T. CNN-Res: deep learning framework for segmentation of acute ischemic stroke lesions on multimodal MRI images. BMC Med Inform Decis Mak. (2023) 23(1):192. 10.1186/s12911-023-02289-y 37752508 PMC10521570

[43] Lenyk Z Park J. Microsoft Vision Model ResNet-50 combines web-scale data and multi-task learning to achieve state-of-the-art. Microsoft Research Blog (2024). Available at: https://www.microsoft.com/en-us/research/blog/microsoft-vision-model-resnet-50-combines-web-scale-data-and-multi-task-learning-to-achieve-state-of-the-art/ (Accessed February 15, 2024).

[44] Drozdzal M Vorontsov E Chartrand G Kadoury S Pal C. The importance of skip connections in biomedical image segmentation. *arXiv* [Preprint]. *arXiv:1608.04117* (2016). Available at: 10.48550/arXiv.1608.04117 (Accessed February 15, 2024).

[45] Zhang Y Liu S Li C Wang J. Application of deep learning method on ischemic stroke lesion segmentation. J Shanghai Jiaotong Univ (Science). (2022) 27(1):99–111. 10.1007/s12204-021-2273-9

[46] Wang S-H Phillips P Sui Y Liu B Yang M Cheng H. Classification of Alzheimer’s disease based on eight-layer convolutional neural network with leaky rectified linear unit and max pooling. J Med Syst. (2018) 42(5):85. 10.1007/s10916-018-0932-7 29577169

[47] Karthik R Gupta U Jha A Rajalakshmi R Menaka R. A deep supervised approach for ischemic lesion segmentation from multimodal MRI using fully convolutional network. Appl Soft Comput. (2019) 84:105685. 10.1016/j.asoc.2019.105685

[48] Karthik R Radhakrishnan M Rajalakshmi R Raymann J. Delineation of ischemic lesion from brain MRI using attention gated fully convolutional network. Biomed Eng Lett. (2021) 11(1):3–13. 10.1007/s13534-020-00178-1 33747599 PMC7930204

[49] Nazari-Farsani S Yu Y Duarte Armindo R Lansberg M Liebeskind DS Albers G Predicting final ischemic stroke lesions from initial diffusion-weighted images using a deep neural network. NeuroImage Clin. (2023) 37:103278. 10.1016/j.nicl.2022.103278 36481696 PMC9727698

[50] Yu Y Xie Y Thamm T Gong E Ouyang J Huang C Use of deep learning to predict final ischemic stroke lesions from initial magnetic resonance imaging. JAMA Network Open. (2020) 3(3):e200772. 10.1001/jamanetworkopen.2020.0772 32163165 PMC7068232

[51] Shore J Johnson R. Axiomatic derivation of the principle of maximum entropy and the principle of minimum cross-entropy. IEEE Trans Inform Theory. (1980) 26(1):26–37. 10.1109/TIT.1980.1056144

[52] Wong KK Cummock JS Li G Ghosh R Xu P Volpi JJ Automatic segmentation in acute ischemic stroke: prognostic significance of topological stroke volumes on stroke outcome. Stroke. (2022) 53(9):2896–905. 10.1161/STROKEAHA.121.037982 35545938

[53] Wei Y-C Huang W-Y Jian C-Y Hsu C-CH Hsu C-C Lin C-P Semantic segmentation guided detector for segmentation, classification, and lesion mapping of acute ischemic stroke in MRI images. NeuroImage Clin. (2022) 35:103044. 10.1016/j.nicl.2022.103044 35597030 PMC9123273

[54] Olivier A Moal O Moal B Munsch F Okubo G Sibon I Active learning strategy and hybrid training for infarct segmentation on diffusion MRI with a U-shaped network. J Med Imaging. (2019) 6(04):1. 10.1117/1.JMI.6.4.044001 PMC6777650 31592439

[55] Clèrigues A Valverde S Bernal J Freixenet J Oliver A Lladó X. Acute and sub-acute stroke lesion segmentation from multimodal MRI. Comput Methods Programs Biomed. (2020) 194:105521. 10.1016/j.cmpb.2020.105521 32434099

[56] Liu L Kurgan L Wu F Wang J. Attention convolutional neural network for accurate segmentation and quantification of lesions in ischemic stroke disease. Med Image Anal. (2020) 65:101791. 10.1016/j.media.2020.101791 32712525

[57] Moon HS Heffron L Mahzarnia A Obeng-Gyasi B Holbrook M Badea CT Automated multimodal segmentation of acute ischemic stroke lesions on clinical MR images. Magn Reson Imaging. (2022) 92:45–57. 10.1016/j.mri.2022.06.001 35688400 PMC9949513

[58] Khezrpour S Seyedarabi H Razavi SN Farhoudi M. Automatic segmentation of the brain stroke lesions from MR flair scans using improved U-net framework. Biomed Signal Process Control. (2022) 78:103978. 10.1016/j.bspc.2022.103978

[59] Hu X Luo W Hu J Guo S Huang W Scott MR Brain SegNet: 3D local refinement network for brain lesion segmentation. BMC Med Imaging. (2020) 20(1):17. 10.1186/s12880-020-0409-2 32046685 PMC7014943

[60] Kumar A Upadhyay N Ghosal P Chowdhury T Das D Mukherjee A CSNet: a new DeepNet framework for ischemic stroke lesion segmentation. Comput Methods Programs Biomed. (2020) 193:105524. 10.1016/j.cmpb.2020.105524 32417618

[61] Zhao B Liu Z Liu G Cao C Jin S Wu H Deep learning-based acute ischemic stroke lesion segmentation method on multimodal MR images using a few fully labeled subjects. In: Chen L, editor. Computational and Mathematical Methods in Medicine. London: Hindawi (2021). p. 3628179. 10.1155/2021/3628179 PMC7867461 33564322

[62] Liu L Wu F-X Wang J. Efficient multi-kernel DCNN with pixel dropout for stroke MRI segmentation. Neurocomputing. (2019) 350:117–27. 10.1016/j.neucom.2019.03.049

[63] Aboudi F Drissi C Kraiem T. Efficient U-net CNN with data augmentation for MRI ischemic stroke brain segmentation. 2022 8th International Conference on Control, Decision and Information Technologies (CoDIT), 1 (2022). p. 724–8

[64] Pinto A Pereira S Meier R Alves V Wiest R Silva CA Enhancing clinical MRI perfusion maps with data-driven maps of complementary nature for lesion outcome prediction. In: Davatzikos C Alberola-Lpez C Gabor F Frangi AF Schnabel JA, editors. Medical Image Computing and Computer Assisted Intervention—MICCAI 2018. Cham: Springer International Publishing (2018). p. 107–15.

[65] Choi Y Kwon Y Lee H Kim BJ Paik MC Won J-H. Ensemble of deep convolutional neural networks for prognosis of ischemic stroke. In: Crimi A Menze B Maier O Reyes M Winzeck S Handels H, editors. Brainlesion: Glioma, Multiple Sclerosis, Stroke and Traumatic Brain Injuries. Cham: Springer International Publishing (2016). p. 231–43.

[66] Kim Y-C Lee J-E Yu I Song H-N Baek I-Y Seong J-K Evaluation of diffusion lesion volume measurements in acute ischemic stroke using encoder-decoder convolutional network. Stroke. (2019) 50(6):1444–51. 10.1161/STROKEAHA.118.024261 31092169

[67] Lee S Sunwoo L Choi Y Jung JH Jung SC Won J-H. Impact of diffusion–perfusion mismatch on predicting final infarction lesion using deep learning. IEEE Access. (2022) 10:97879–87. 10.1109/ACCESS.2022.3204048

[68] Karthik R Menaka R Hariharan M Won D. Ischemic lesion segmentation using ensemble of multi-scale region aligned CNN. Comput Methods Programs Biomed. (2021) 200:105831. 10.1016/j.cmpb.2020.105831 33223277

[69] Zhang L Song R Wang Y Zhu C Liu J Yang J Ischemic stroke lesion segmentation using multi-plane information fusion. IEEE Access. (2020) 8:45715–25. 10.1109/ACCESS.2020.2977415

[70] Ou Y Yuan Y Huang X Wong K Volpi J Wang JZ LambdaUNet: 2.5D Stroke Lesion Segmentation of Diffusion-weighted MR Images. arXiv [Preprint]. arXiv:2104.13917 (2021). Available at: 10.48550/arXiv.2104.13917 (Accessed December 01, 2023).

[71] Vupputuri A Gupta A Ghosh N. MCA-DN: multi-path convolution leveraged attention deep network for salvageable tissue detection in ischemic stroke from multi-parametric MRI. Comput Biol Med. (2021) 136:104724. 10.1016/j.compbiomed.2021.104724 34388469

[72] Abdmouleh N Echtioui A Kallel F Hamida AB. Modified U-net architecture based ischemic stroke lesions segmentation. 2022 IEEE 21st International Conference on Sciences and Techniques of Automatic Control and Computer Engineering (STA) (2022). p. 361–5

[73] Duan W Zhang L Colman J Gulli G Ye X. Multi-modal brain segmentation using hyper-fused convolutional neural network. In: Mostafa S Mohamad H Vinod K Maryam RJ Chantal T Thomas W, editors. Machine Learning in Clinical Neuroimaging. Cham: Springer International Publishing (2021). p. 82–91.

[74] Lucas C Kemmling A Mamlouk AM Heinrich MP. Multi-scale neural network for automatic segmentation of ischemic strokes on acute perfusion images. 2018 IEEE 15th International Symposium on Biomedical Imaging (ISBI 2018) (2018). p. 1118–21

[75] Li C Ji P. TernausNet-based segmentation of cerebral infarction in magnetic resonance images. J Radiat Res Appl Sci. (2023) 16(3):100619. 10.1016/j.jrras.2023.100619

[76] Liu Z Cao C Ding S Liu Z Han T Liu S. Towards clinical diagnosis: automated stroke lesion segmentation on multi-spectral MR image using convolutional neural network. IEEE Access. (2018) 6:57006–16. 10.1109/ACCESS.2018.2872939

[77] Cornelio LKS del Castillo MA Naval PC Jr. U-ISLES: ischemic stroke lesion segmentation using U-net. In: Bhatia R Arai K Kapoor S, editors. Intelligent Systems and Applications. Cham: Springer International Publishing (2019). pp. 326–36.

[78] Wu Z Zhang X Li F Wang S Huang L Li J. W-Net: a boundary-enhanced segmentation network for stroke lesions. Expert Syst Appl. (2023) 230:120637. 10.1016/j.eswa.2023.120637

[79] Jeong H Lim H Yoon C Won J Lee GY de la Rosa E Robust ensemble of two different multimodal approaches to segment 3D ischemic stroke segmentation using brain tumor representation among multiple center datasets. J Imaging Inform Med. (2024) 37(5):2375–89. 10.1007/s10278-024-01099-6 38693333 PMC11522214

[80] Gi C An X Li T Liu S Ming D. St-RegSeg: an unsupervised registration-based framework for multimodal magnetic resonance imaging stroke lesion segmentation. Quant Imaging Med Surg. (2024) 14(12):9459–76. 10.21037/qims-24-725 39698611 PMC11651977

[81] Brott T Adams HP Olinger CP Marler JR Barsan WG Biller J Measurements of acute cerebral infarction: a clinical examination scale. Stroke. (1989) 20(7):864–70. 10.1161/01.STR.20.7.864 2749846

[82] Winzeck S Hakim A McKinley R Pinto JAADSR Alves V Silva C ISLES 2016 and 2017-benchmarking ischemic stroke lesion outcome prediction based on multispectral MRI. Front Neurol. (2018) 9:2–10. 10.3389/fneur.2018.00679 30271370 PMC6146088

[83] Lansberg MG Straka M Kemp S Mlynash M Wechsler LR Jovin TG MRI profile and response to endovascular reperfusion after stroke (DEFUSE 2): a prospective cohort study. Lancet Neurol. (2012) 11(10):860–7. 10.1016/S1474-4422(12)70203-X 22954705 PMC4074206

[84] Marks MP Heit JJ Lansberg MG Kemp S Christensen S Derdeyn CP Endovascular treatment in the DEFUSE 3 study. Stroke. (2018) 49(8):2000–3. 10.1161/STROKEAHA.118.022147 29986935 PMC6202142

[85] Zaharchuk G Marks MP Do HM Bammer R Lansberg M Kemp S Abstract W MP16: introducing the imaging the collaterals in acute stroke (iCAS) multicenter MRI trial. Stroke. (2015) 46(suppl_1). 10.1161/str.46.suppl_1.wmp16

[86] Karthik R Menaka R Johnson A Anand S. Neu2roimaging and deep learning for brain stroke detection—a review of recent advancements and future prospects. Comput Methods Programs Biomed. (2020) 197:105728. 10.1016/j.cmpb.2020.105728 32882591

[87] Ostmeier S Axelrod B Isensee F Bertels J Mlynash M Christensen S USE-evaluator: performance metrics for medical image segmentation models supervised by uncertain, small or empty reference annotations in neuroimaging. Med Image Anal. (2023) 90:102927. 10.1016/j.media.2023.102927 37672900 PMC11997713

[88] Kendall A Gal Y. What uncertainties do we need in Bayesian deep learning for computer vision? *arXiv* [Preprint]. *arXiv:1703.04977* (2017). Available at: 10.48550/arXiv.1703.04977 (Accessed December 01, 2023).

[89] Pavlakis SG Hirtz DG deVeber G. Pediatric stroke: opportunities and challenges in planning clinical trials. Pediatr Neurol. (2006) 34(6):433–5. 10.1016/j.pediatrneurol.2005.09.009 16765819

[90] Ospel J Singh N Ganesh A Goyal M. Sex and gender differences in stroke and their practical implications in acute care. J Stroke. (2023) 25(1):16–25. 10.5853/jos.2022.04077 36746379 PMC9911850

[91] Wulms N Redmann L Herpertz C Bonberg N Berger K Sundermann B The effect of training sample size on the prediction of white matter hyperintensity volume in a healthy population using BIANCA. Front Aging Neurosci. (2022) 13:1–12. 10.3389/fnagi.2021.720636 PMC8812526 35126084

[92] Iglovikov V Shvets A. TernausNet: U-Net with VGG11 encoder pre-trained on ImageNet for image segmentation. *arXiv* [Preprint]. *arXiv:1801.05746* (2018). Available at: 10.48550/arXiv.1801.05746 (Accessed December 01, 2023).

[93] Deng J Dong W Socher R Li L-J Kai L Li F-F. Imagenet: a large-scale hierarchical image database. 2009 IEEE Conference on Computer Vision and Pattern Recognition (2009). p. 248–55

[94] Baid U Ghodasara S Mohan S Bilello M Calabrese E Colak E The RSNA-ASNR-MICCAI BraTS 2021 benchmark on brain tumor segmentation and radiogenomic classification. *arXiv* [Preprint]. *arXiv:2107.02314* (2021). Available at: 10.48550/arXiv.2107.02314 (Accessed December 01, 2023).

[95] Cui L Fan Z Yang Y Liu R Wang D Feng Y Deep learning in ischemic stroke imaging analysis: a comprehensive review. BioMed Res Int. (2022) 2022:2456550. 10.1155/2022/2456550 36420096 PMC9678444

[96] Wardlaw JM Farrall AJ. Diagnosis of stroke on neuroimaging. Br Med J. (2004) 328(7441):655–6. 10.1136/bmj.328.7441.655 15031215 PMC381208

[97] Karthik R Menaka R. Computer-aided detection and characterization of stroke lesion—a short review on the current state-of-the art methods. Imaging Sci J. (2018) 66(1):1–22. 10.1080/13682199.2017.1370879

[98] Schick F Pieper CC Kupczyk P Almansour H Keller G Springer F 1.5 vs. 3 tesla magnetic resonance imaging. Invest Radiol. (2021) 56(11):680–91. 10.1097/RLI.0000000000000812 34324464

[99] Simonsen CZ Madsen MH Schmitz ML Mikkelsen IK Fisher M Andersen G. Sensitivity of diffusion- and perfusion-weighted imaging for diagnosing acute ischemic stroke is 97.5%. Stroke. (2015) 46(1):98–101. 10.1161/STROKEAHA.114.007107 25388415

[100] Sitburana O Koroshetz WJ. Magnetic resonance imaging: implication in acute ischemic stroke management. Curr Atheroscler Rep. (2005) 7(4):305–12. 10.1007/s11883-005-0023-3 15975324

[101] Kakuda W Lansberg MG Thijs VN Kemp SM Bammer R Wechsler LR Optimal definition for PWI/DWI mismatch in acute ischemic stroke patients. J Cereb Blood Flow Metab. (2008) 28(5):887–91. 10.1038/sj.jcbfm.9600604 18183031 PMC3985735

[102] Zhu H Jiang L Zhang H Luo L Chen Y Chen Y. An automatic machine learning approach for ischemic stroke onset time identification based on DWI and FLAIR imaging. NeuroImage Clin. (2021) 31:102744. 10.1016/j.nicl.2021.102744 34245995 PMC8271155

[103] Avesta A Hossain S Lin M Aboian M Krumholz HM Aneja S. Comparing 3D, 2.5D, and 2D approaches to brain image auto-segmentation. Bioengineering. (2023) 10(2):181. 10.3390/bioengineering10020181 36829675 PMC9952534

[104] Yu L Yang X Chen H Qin J Heng PA. Volumetric ConvNets with mixed residual connections for automated prostate segmentation from 3D MR images. Proc AAAI Conf Artif Intell. (2017) 31(1):69–71. 10.1609/aaai.v31i1.10510

[105] Hashemi SR Mohseni Salehi SS Erdogmus D Prabhu SP Warfield SK Gholipour A. Asymmetric loss functions and deep densely-connected networks for highly-imbalanced medical image segmentation: application to multiple sclerosis lesion detection. IEEE Access. (2019) 7:1721–35. 10.1109/ACCESS.2018.2886371 PMC6746414 31528523

[106] Zhang Y Liu S Li C Wang J. Rethinking the dice loss for deep learning lesion segmentation in medical images. J Shanghai Jiaotong Univ (Science). (2021) 26(1):93–102. 10.1007/s12204-021-2264-x

[107] Rachmadi MF Poon C Skibbe H. Improving segmentation of objects with varying sizes in biomedical images using instance-wise and center-of-instance segmentation loss function. *arXiv* [Preprint]. *arXiv:2304.06229* (2023). Available at: 10.48550/arXiv.2304.06229 (Accessed December 01, 2023).

[108] Inamdar MA Raghavendra U Gudigar A Chakole Y Hegde A Menon GR A review on computer aided diagnosis of acute brain stroke. Sensors. (2021) 21(24):24–5. 10.3390/s21248507 PMC8707263 34960599

[109] Wang S Tan S Gao Y Liu Q Ying L Xiao T Learning joint-sparse codes for calibration-free parallel MR imaging. IEEE Trans Med Imaging. (2018) 37(1):251–61. 10.1109/TMI.2017.2746086 28866485

[110] Babu MS Vijayalakshmi V. A review on acute/sub-acute ischemic stroke lesion segmentation and registration challenges. Multimed Tools Appl. (2019) 78(2):2481–506. 10.1007/s11042-018-6344-3

[111] Cao Y Liu W Zhang S Xu L Zhu B Cui H Detection and localization of myocardial infarction based on multi-scale ResNet and attention mechanism. Front Physiol. (2022) 13:3–12. 10.3389/fphys.2022.783184 PMC8832050 35153827

[112] Liu C Yin Y Sun Y Ersoy OK. Multi-scale ResNet and BiGRU automatic sleep staging based on attention mechanism. PLoS One. (2022) 17(6):e0269500. 10.1371/journal.pone.0269500 35709101 PMC9202858

[113] Marcos L Quint F Babyn P Alirezaie J. Dilated convolution ResNet with boosting attention modules and combined loss functions for LDCT image denoising. 2022 44th Annual International Conference of the IEEE Engineering in Medicine & Biology Society (EMBC) (2022). p. 1548–51 10.1109/EMBC48229.2022.9870993 36086586

[114] Schmidt RL Factor RE. Understanding sources of bias in diagnostic accuracy studies. Arch Pathol Lab Med. (2013) 137(4):558–65. 10.5858/arpa.2012-0198-RA 23544945

[115] Richardson M Garner P Donegan S. Interpretation of subgroup analyses in systematic reviews: a tutorial. Clin Epidemiol Glob Health. (2019) 7(2):192–8. 10.1016/j.cegh.2018.05.005

[116] Hedges L. Statistical Methods for Meta-Analysis. New York, NY: Elsevier (1985).

[117] Reinke A Tizabi MD Baumgartner M Eisenmann M Heckmann-Nötzel D Rädsch T Understanding metric-related pitfalls in image analysis validation. Nat Methods. (2024) 21:182–94. 10.1038/s41592-023-02150-0 38347140 PMC11181963

[118] Karpathy A Fei-Fei L. Deep visual-semantic alignments for generating image descriptions. IEEE Trans Pattern Anal Mach Intell. (2017) 39(4):664–76. 10.1109/TPAMI.2016.2598339 27514036

[119] Pellegrini E Ballerini L Hernandez MCV del Chappell FM González-Castro V Anblagan D Machine learning of neuroimaging for assisted diagnosis of cognitive impairment and dementia: a systematic review. Alzheimer’s Dement Diagn Assess Dis Monit. (2018) 10(1):519–35. 10.1016/j.dadm.2018.07.004 PMC6197752 30364671

[120] Springenberg JT Dosovitskiy A Brox T Riedmiller M. Striving for simplicity: the all convolutional net. *arXiv* [Preprint]. *arXiv:1412.6806* (2014). Available at: 10.48550/arXiv.1412.6806 (Accessed December 01, 2023).

[121] Zeiler MD Fergus R. Visualizing and understanding convolutional networks. *arXiv* [Preprint]. *arXiv:1311.2901*. (2013). Available at: 10.48550/arXiv.1311.2901 (Accessed December 01, 2023).

[122] Qin S Zhang Z Jiang Y Cui S Cheng S Li Z. NG-NAS: node growth neural architecture search for 3D medical image segmentation. Comput Med Imaging Graph. (2023) 108:102268. 10.1016/j.compmedimag.2023.102268 37379669

[123] Allcock J Vangone A Meyder A Adaszewski S Strahm M Hsieh C-Y The prospects of Monte Carlo antibody loop modelling on a fault-tolerant quantum computer. Front Drug Discov. (2022) 2:3–7. 10.3389/fddsv.2022.908870

