# Supplementary material for: Deep learning for MRI-based acute and subacute ischaemic stroke lesion segmentation—a systematic review, meta-analysis, and pilot evaluation of key results
Source: Front Med Technol. 2025 Jun 10;7:1491197. doi: 10.3389/fmedt.2025.1491197 (PMC12185483; doi:10.3389/fmedt.2025.1491197)
Supplement: Supplementary Data Sheet 1C — Supplementary Data C. [file Datasheet1.docx]

**Deep Learning for MRI-based Acute and Subacute Ischaemic Stroke Lesion Segmentation - A Systematic Review, Meta-Analysis, and Pilot Evaluation of Key Results**

- **SUPPLEMENTARY DATA –**

# **A - Full Search Strategy**

## Information Sources

- Electronic databases: MEDLINE, PubMed, IEEE Xplore, Web of Science, ScienceDirect, Springer
- Hand-search all [MICCAI](http://www.miccai.org/) conference proceedings (2015–2023) on [Springer](https://link.springer.com/conference/miccai)
- Hand-search all [MIDL](https://www.midl.io/) conference proceedings (2018–2023) on [OpenReview.net](https://openreview.net/group?id=MIDL.io&referrer=%5BHomepage%5D(%2F))

## Step 1: List Subject Components

PICO analysis:

- Population (P): Patients with acute and/or subacute ischaemic stroke lesions on brain MRI
- Intervention (I): Deep learning model architectures for MRI-based stroke lesion segmentation
- Comparison (C): Compare model architectures and modelling approaches proposed in retained studies in order to recommend best practices for developing
- Outcomes (O):
  - Assess the accuracy of the deep learning stroke lesion segmentation algorithms described in the retained studies
  - Assess the generalizability of the deep learning stroke lesion segmentation algorithms described in the retained studies

List the subject components that were identified from the research question based on the PICO analysis above:

- Accuracy
- Acute ischemic stroke
- Deep learning
- Lesion segmentation
- Magnetic resonance imaging

## Step 2: Expand Subject Components

Related terms, alternative spelling, and synonyms with reference to each subject component have been identified, and summarized in the table below:

**Table S1. Terms related to subject components**

| **What would be the optimal deep learning model architecture for acute and/or subacute ischaemic stroke lesion segmentation on brain MRI?** | | | | |
| --- | --- | --- | --- | --- |
| **Accuracy** | **Acute ischemic stroke** | **Deep learning** | **Lesion segmentation** | **Magnetic resonance imaging** |
| 1. Area under curve 2. ASSD 3. AUC 4. Average symmetric surface distance 5. Dice 6. Hausdorff distance 7. Precision 8. Recall 9. Sensitivity 10. Similarity index 11. Specificity 12. Validity | 1. AIS 2. Atheroma 3. Cerebral infarction 4. Embolism 5. Infarct 6. Ischemia 7. Ischemic 8. Lacunar 9. Large vessel occlusion 10. Small vessel disease 11. Stroke 12. Stroke prognosis 13. Subacute stroke | 1. ANN 2. Artificial neural networks 3. CNN 4. Computer-aided diagnostics 5. Convolutional neural networks 6. Deep neural networks 7. DenseNet 8. Neural networks | 1. Automatic segmentation 2. Image segmentation 3. Lesion 4. Lesion detection 5. Medical image segmentation 6. Parcellation 7. Stroke lesions 8. Supervised segmentation 9. Unsupervised segmentation | 1. ADC 2. Apparent diffusion coefficient 3. Brain biomarker 4. Diffusion-weighted imaging 5. DWI 6. Imaging biomarker 7. MR images 8. MRI 9. Multimodal MRI 10. Neuroimaging 11. Perfusion–diffusion 12. PWI |

## Step 3: Execute Searches

### **MEDLINE**

Use the OVID gateway to access the published articles that are related to our research question, by following these steps:

1. Login via: <https://ovidsp.dc1.ovid.com/ovid-new-b/ovidweb.cgi>
2. Select the following resources:
   - Ovid MEDLINE(R) and In-Process, In-Data-Review & Other Non-Indexed Citations
   - Ovid MEDLINE(R)
3. Run the following search:

1. acute stroke.mp. or exp Stroke/

2. exp Brain Ischemia/ or Brain Infarction/ or Brain Injuries/

3. Cerebral Infarction/cf, di, dg, et [Cerebrospinal Fluid, Diagnosis, Diagnostic Imaging, Etiology]

4. Cerebral Small Vessel Diseases/bl, cf, di, dg, et [Blood, Cerebrospinal Fluid, Diagnosis, Diagnostic Imaging, Etiology]

5. exp Embolic Stroke/ or "Intracranial Embolism and Thrombosis"/

6. Infarction/bl, di, dg, et [Blood, Diagnosis, Diagnostic Imaging, Etiology]

7. exp Ischemic Stroke/

8. Stroke, Lacunar/

9. subacute stroke.mp.

10. exp Thrombotic Stroke/

11. 1 or 2 or 3 or 4 or 5 or 6 or 7 or 8 or 9 or 10

12. Algorithms/

13. Artificial Intelligence/

14. Diagnosis, Computer-Assisted/

15. Image Interpretation, Computer-Assisted/

16. Image Processing, Computer-Assisted/

17. Neural Networks, Computer/ or Machine Learning/ or Pattern Recognition, Automated/

18. Imaging, Three-Dimensional/ or automatic segmentation.mp.

19. Artificial neural networks.mp.

20. convolutional neural networks.mp.

21. exp Deep Learning/ or deep learning based segmentation.mp.

22. fully automated segmentation.mp.

23. lesion segmentation.mp.

24. attention mechanisms.mp.

25. fully convolutional networks.mp.

26. uncertainty quantification.mp.

27. 12 or 13 or 14 or 15 or 16 or 17 or 18 or 19 or 20 or 21 or 22 or 23 or 24 or 25 or 26

28. exp Diffusion Magnetic Resonance Imaging/ or apparent diffusion coefficient.mp.

29. Biomarkers/

30. exp Magnetic Resonance Imaging/ or DWI.mp.

31. MR images.mp.

32. MRI.mp.

33. FLAIR.mp.

34. T1w.mp.

35. T2w.mp.

36. multimodal MRI.mp.

37. Functional Neuroimaging/ or Neuroimaging/

38. Perfusion Index/ or exp Perfusion Imaging/ or Perfusion/

39. PWI.mp.

40. brain imaging.mp.

41. 28 or 29 or 30 or 31 or 32 or 33 or 34 or 35 or 36 or 37 or 38 or 39 or 40

42. Area Under Curve/

43. AUC.mp.

44. average symmetric surface distance.mp.

45. Dice.mp.

46. hausdorff distance.mp.

47. recall.mp.

48. similarity index.mp.

49. 42 or 43 or 44 or 45 or 46 or 47 or 48

50. 11 and 27 and 41 and 49

51. limit 50 to (english language and humans and yr="2015 - 2023")

### **PubMed**

1. Go to the URL: [Advanced Search Results - PubMed (nih.gov)](https://pubmed.ncbi.nlm.nih.gov/advanced/)
2. Run the following search: (((((((((acute stroke[Title/Abstract]) OR (brain ischemia[Title/Abstract])) OR (embolic stroke[Title/Abstract])) OR (infarction[Title/Abstract])) OR (ischemic stroke[Title/Abstract])) OR (subacute stroke[Title/Abstract])) OR (thrombotic stroke[Title/Abstract])) AND (((((((neural network[Title/Abstract]) OR (deep learning[Title/Abstract])) OR (convolutional network[Title/Abstract])) OR (lesion segmentation[Title/Abstract])) OR (brain lesion[Title/Abstract])) OR (attention mechanism[Title/Abstract])) OR (uncertainty quantification[Title/Abstract]))) AND (((((((((apparent diffusion coefficient[Title/Abstract]) OR (magnetic resonance imaging[Title/Abstract])) OR (diffusion-weighted[Title/Abstract])) OR (DWI[Title/Abstract])) OR (perfusion imaging[Title/Abstract])) OR (PWI[Title/Abstract])) OR (MRI[Title/Abstract])) OR (neuroimaging[Title/Abstract])) OR (brain imaging[Title/Abstract]))) AND ((((((dice[Title/Abstract]) OR (similarity index[Title/Abstract])) OR (average symmetric surface distance[Title/Abstract])) OR (ASSD[Title/Abstract])) OR (hausdorff distance[Title/Abstract])) OR (accuracy[Title/Abstract]))
3. Update the “RESULTS BY YEAR” bar chart filter to 2015–2023

### **Web of Science**

1. Go to the URL: <https://www.webofscience.com/wos/woscc/advanced-search>
2. Run the following search:

TS = ("acute stroke" OR "brain ischemia" OR "embolic stroke" OR "infarction" OR "ischemic stroke" OR "subacute stroke" OR “thrombotic stroke”)

AND

TI = (“stroke”)

AND

TS = ("neural network" OR "deep learning" OR "convolutional network" OR "lesion segmentation" OR "machine learning" OR "brain segmentation" OR “brain lesion” OR “automatic segmentation” OR “automated segmentation” OR “fully convolutional” OR “attention mechanism” OR “uncertainty quantification”)

AND

TS = ("apparent diffusion coefficient" OR "magnetic resonance imaging" OR "diffusion-weighted" OR "DWI" OR "perfusion imaging" OR "PWI" OR “MRI” OR “MR imaging” OR “FLAIR” OR “T1w” OR “neuroimaging” OR “brain imaging”)

AND

TS = ("area under curve" OR "AUC" OR "dice" OR "similarity index" OR "recall" OR "average symmetric surface distance" OR “ASSD” OR “hausdorff distance” OR “accuracy” OR “sensitivity” OR “specificity”)

Add date range: From “2015-01-01” to “2023-12-31”

### **IEEE Xplore**

1. Go to the URL: <https://ieeexplore.ieee.org/search/advanced>
2. Run several searches combining above-mentioned subject components:
   - Example search: ("All Metadata":acute) AND ("All Metadata":stroke) AND ("All Metadata":deep learning) AND ("All Metadata":segmentation) AND ("All Metadata":MRI)

Year range: From 2015 To 2023

### **ScienceDirect**

1. Go to the URL: <https://www.sciencedirect.com/search/entry>
2. Run the following advanced search (constrained by the limit of 8 boolean operators per search):

(acute stroke OR acute ischemic) AND (lesion segmentation OR automated segmentation) AND (MRI OR magnetic resonance imaging) AND (deep learning OR neural network)

1. Update search filter “Article type” by selecting “Research articles”
2. Update search filter “Years” by selecting 2015, 2016, 2017, 2018, 2019, 2020, 2021, 2022, 2023

### **Springer**

1. Go to the URL: <https://link.springer.com/advanced-search>
2. Run several searches combining above-mentioned subject components:
   - Example: acute AND stroke AND deep AND learning AND MRI AND lesion AND segmentation

Start: year 2015; End: year 2023

Add filter Content Type = “Article”

1. Hand-search the “Table of contents” of each of the following conference proceedings:
   - Medical Image Computing and Computer Assisted Intervention – MICCAI 2015
   - Medical Image Computing and Computer Assisted Intervention – MICCAI 2016
   - Medical Image Computing and Computer Assisted Intervention – MICCAI 2017
   - Medical Image Computing and Computer Assisted Intervention – MICCAI 2018
   - Medical Image Computing and Computer Assisted Intervention – MICCAI 2019
   - Medical Image Computing and Computer Assisted Intervention – MICCAI 2020
   - Medical Image Computing and Computer Assisted Intervention – MICCAI 2021
   - Medical Image Computing and Computer Assisted Intervention – MICCAI 2022
   - Medical Image Computing and Computer Assisted Intervention – MICCAI 2023

### **OpenReview.net**

1. Go to the URL: <https://openreview.net/group?id=MIDL.io&referrer=%5BHomepage%5D(%2F)>
2. Go to “MIDL 20xx Conference”
3. In “Oral Papers” and “Poster Papers”, hand-search the following conference proceedings:
   - MIDL 2018
   - MIDL 2019
   - MIDL 2020
   - MIDL 2021
   - MIDL 2022
   - MIDL 2023

## Step 4: Manual Citation Tracking

The citation tracking process will begin after the completion of all above searches. It will go as follows:

1. Locate the references section of the first paper that was retained following previous search
2. Examine the list of references and identify those that are directly related to this project
3. Make an abstract-based assessment
4. If conclusive, continue with the full-text assessment
5. If conclusive too, add the paper to the list of retained papers for this review
6. Repeat the process for every retained paper following previous search

## Step 5: Hand-search Journals

Hand-search the following journals, for the sake of comparing the results of this manual search to the results of the previously executed “advanced” searches:

- Stroke: <https://www.ahajournals.org/journal/str>
  - Search query: “Lesion segmentation AND Stroke AND MRI AND Deep learning AND Dice”
  - Date range: from Jan2015-Dec2023
  - Search results: 97 papers
  - Recall score = TP/(TP+FN) = 2/2 = 1.0
- NeuroImage: Clinical: <https://www.sciencedirect.com/journal/neuroimage-clinical>
  - Search query: “Lesion segmentation" AND "Stroke" AND "MRI" AND "Deep learning" AND "Dice"
  - Data range: Jan2015-Dec2023
  - Search results: 17 papers
  - Recall score = 1.0

## Step 6: Search Summary

- Summary of the main reasons why papers were excluded after the title and/or abstract search:
  - Results of the segmentation on the validation or test set not reported, but rather on the train set only, which is unfair when comparing to remaining retained studies
  - Results of the segmentation not reported due to the fact that there is a classification algorithm following the segmentation (i.e., post-processing), and only the results of the latter are reported (e.g., DeepMedic)
  - Predominant inclusion of cases of hyper-acute and/or chronic stroke (e.g., ATLAS 2.0 dataset), rather than acute and/or subacute
  - Machine learning or reinforcement learning algorithm proposed rather than deep learning algorithm
  - Unsure which input image modality was used, or CT images used instead of MRI, or CT used in conjunction with MRI images
  - Focuses predominantly on haemorrhagic stroke rather than ischemic stroke
  - Focuses predominantly on WMH segmentation rather than stroke lesion segmentation
  - Results of the segmentation as a whole not reported, but rather reported specifically for core lesion segmentation and/or penumbra lesion segmentation
  - No information was provided on the model architecture used (e.g., U-Net)
  - Input data incorporated synthetic data created just for the purpose of the experiment
  - Focuses mostly on brain tumour segmentation rather than stroke lesion segmentation
  - Research paper neither open access not accessible via institution credentials
  - Study does outcome prediction on whether an image contains 1+ lesion(s) or not, rather than doing lesion segmentation
  - Study focusing on stroke lesion segmentation in sub-part or specific part of the brain (e.g., brainstem)

# **B – Additional Visualisations**

## AG-UResNet50 architecture


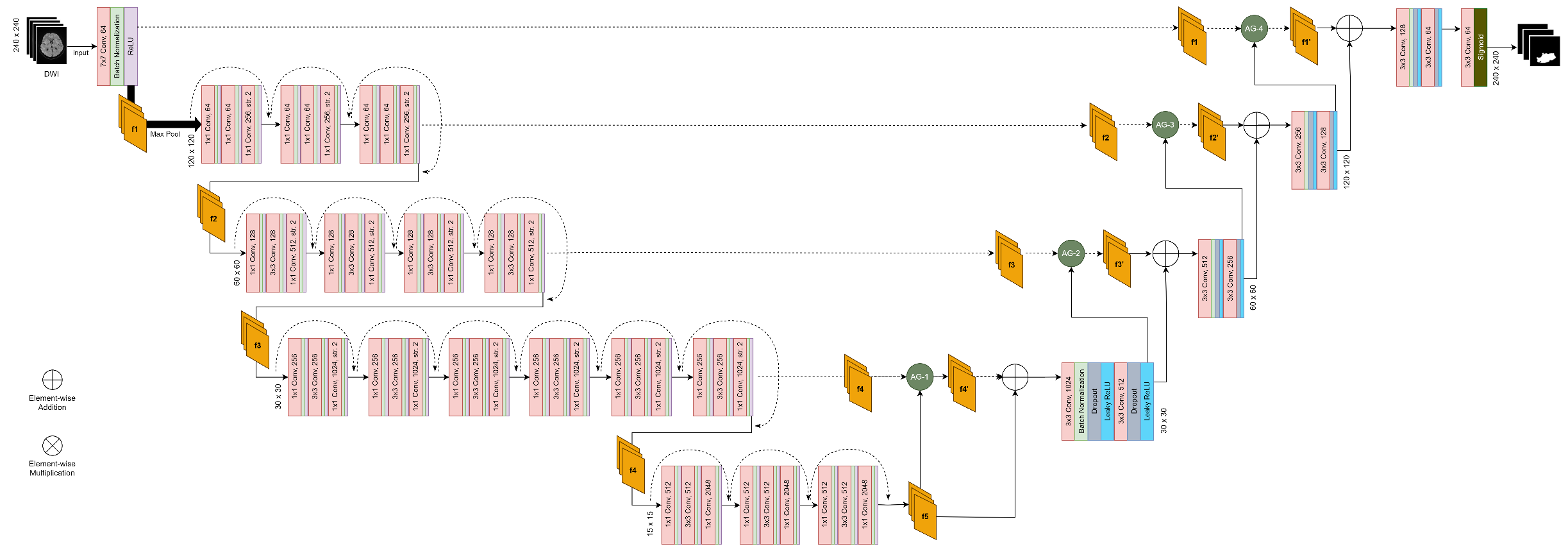


Please feel free to zoom-in for better visualisation.

## Single-feature Analysis


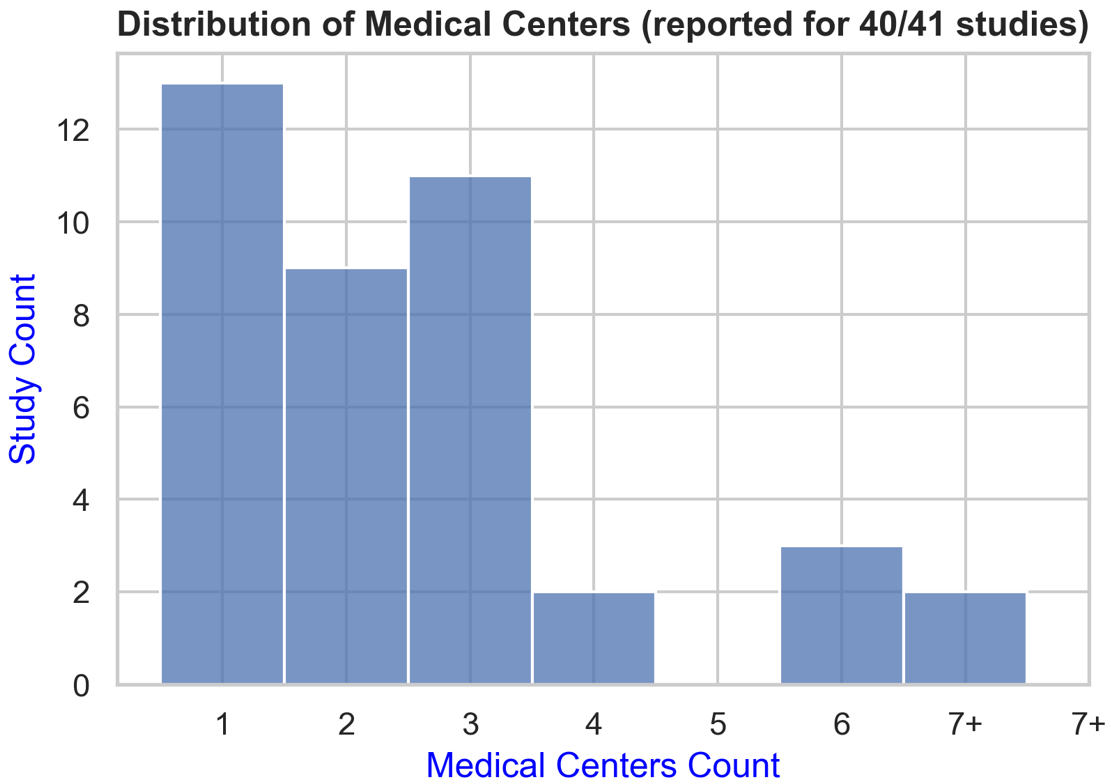


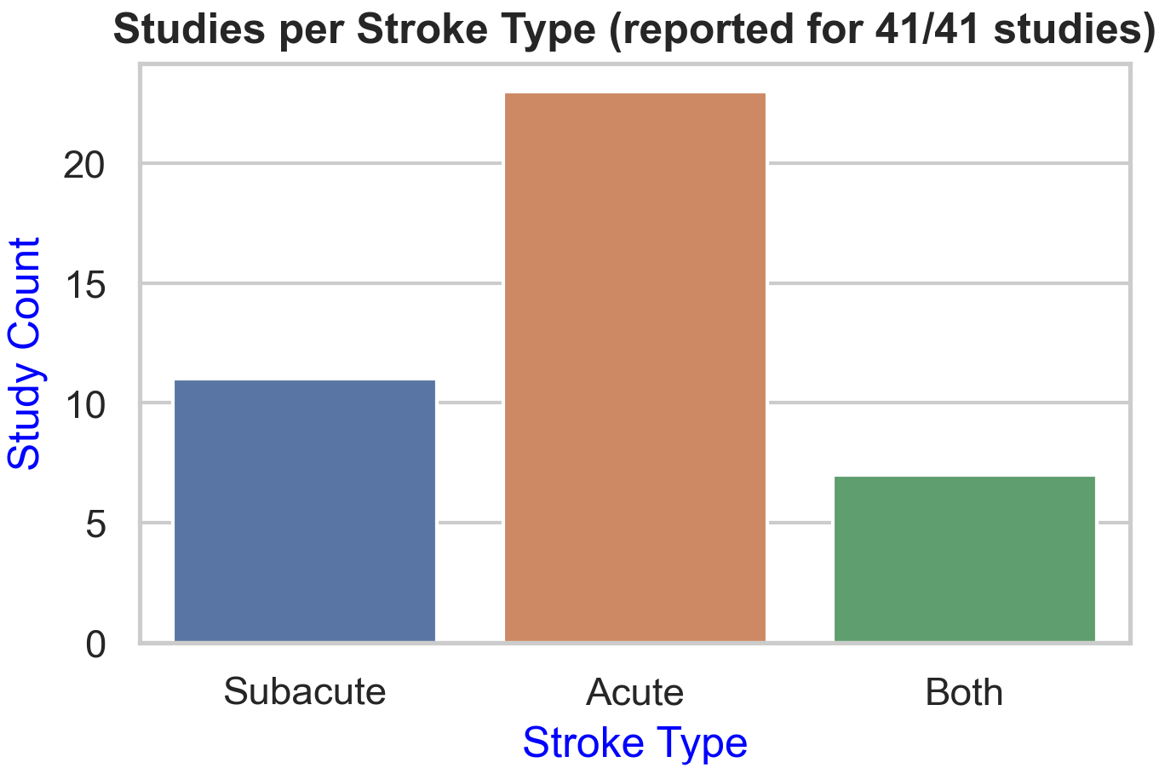


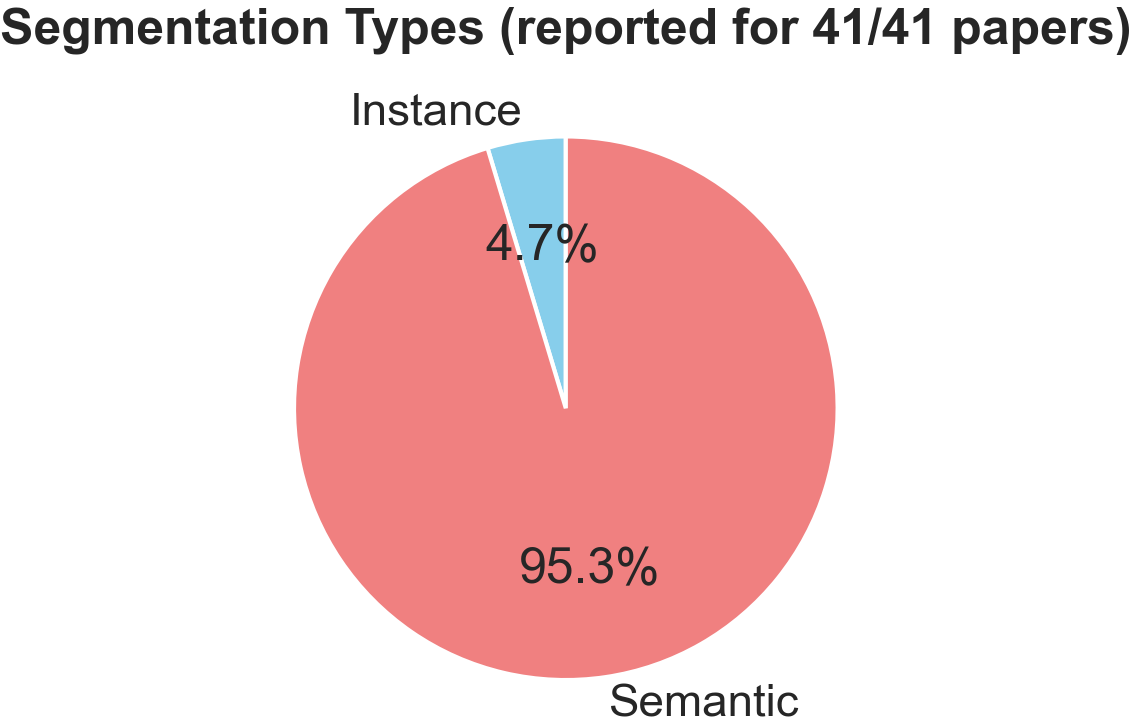


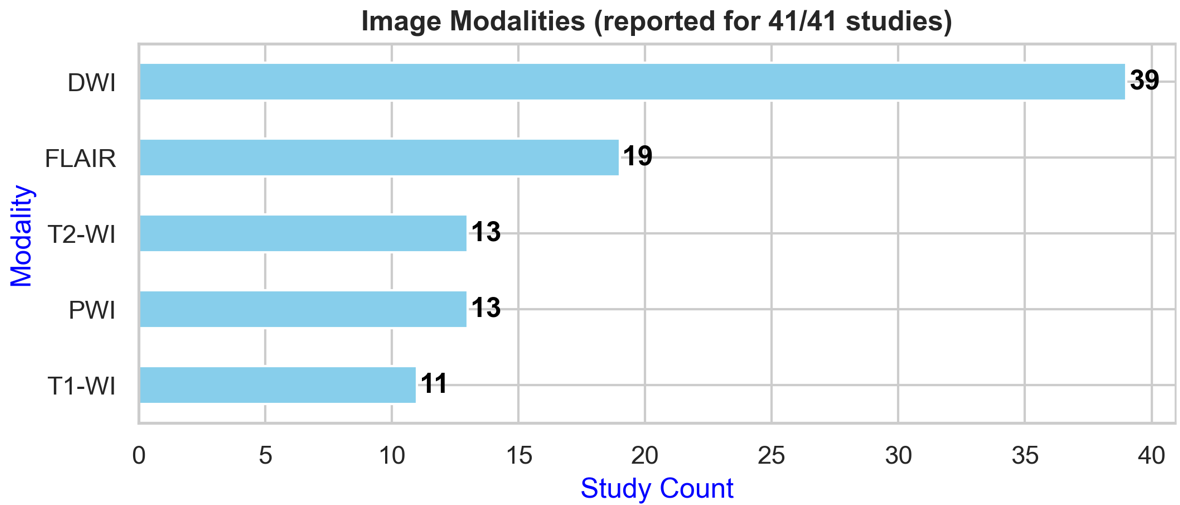


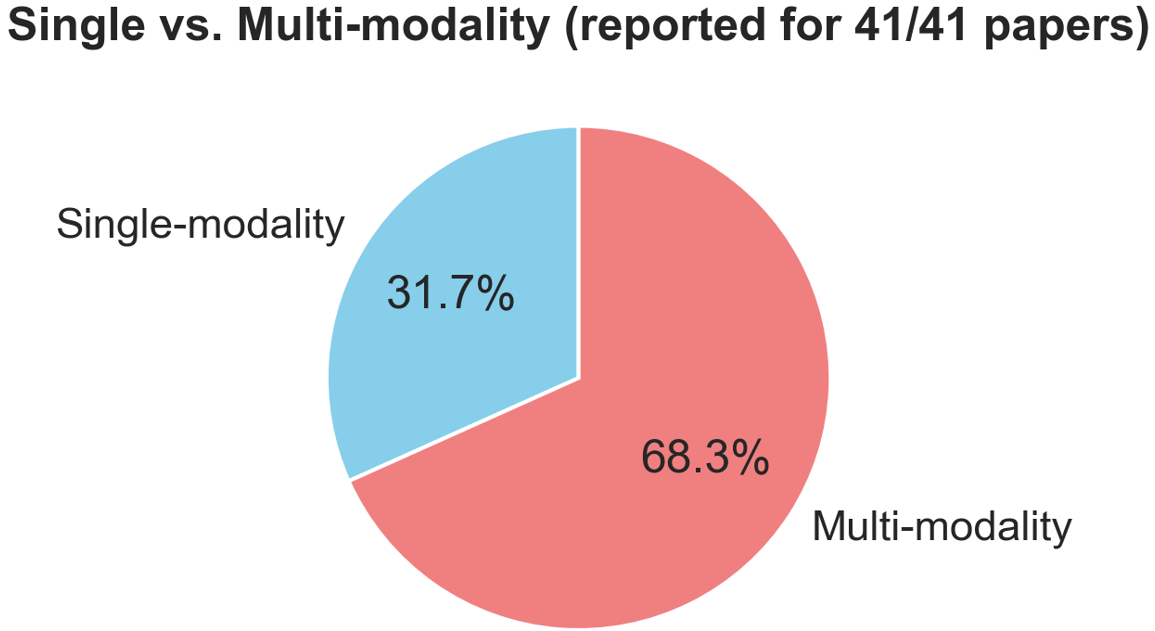


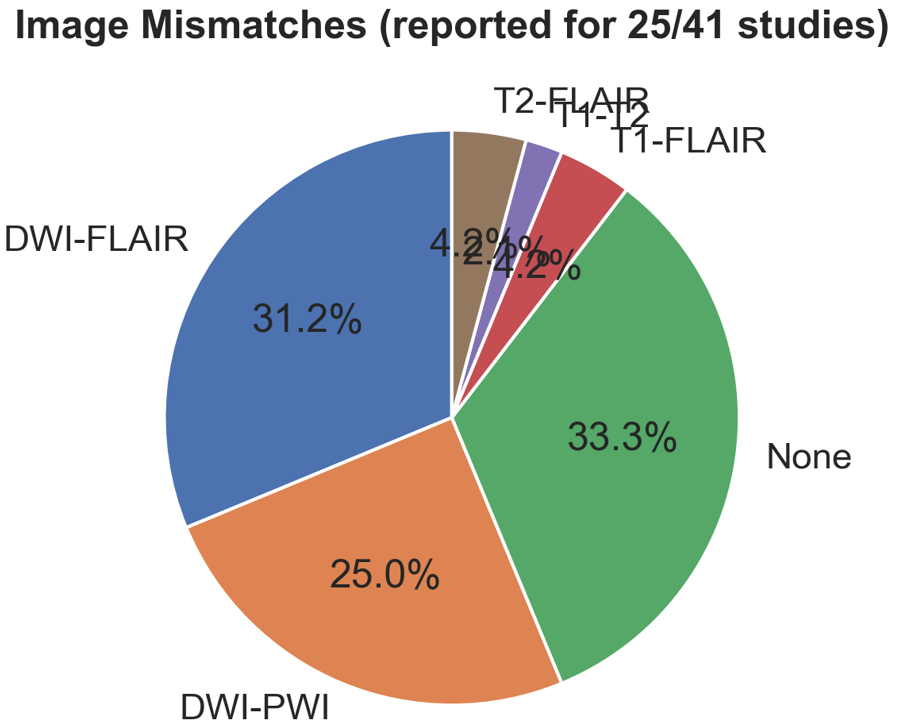


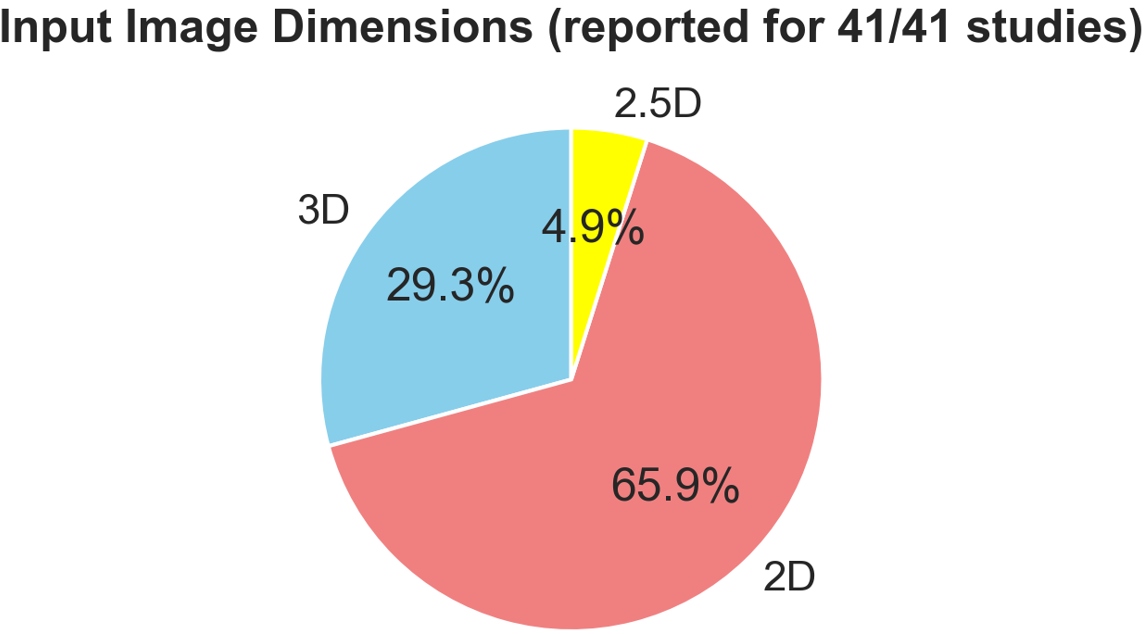


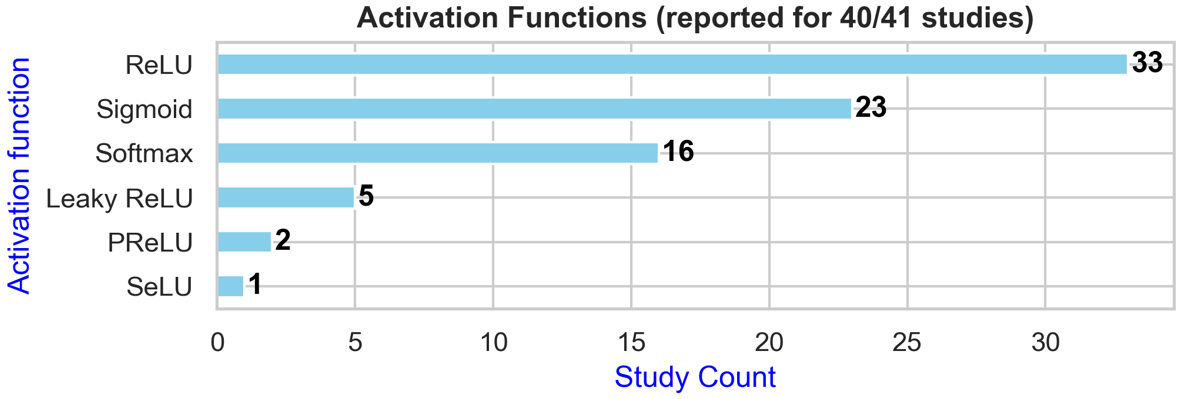


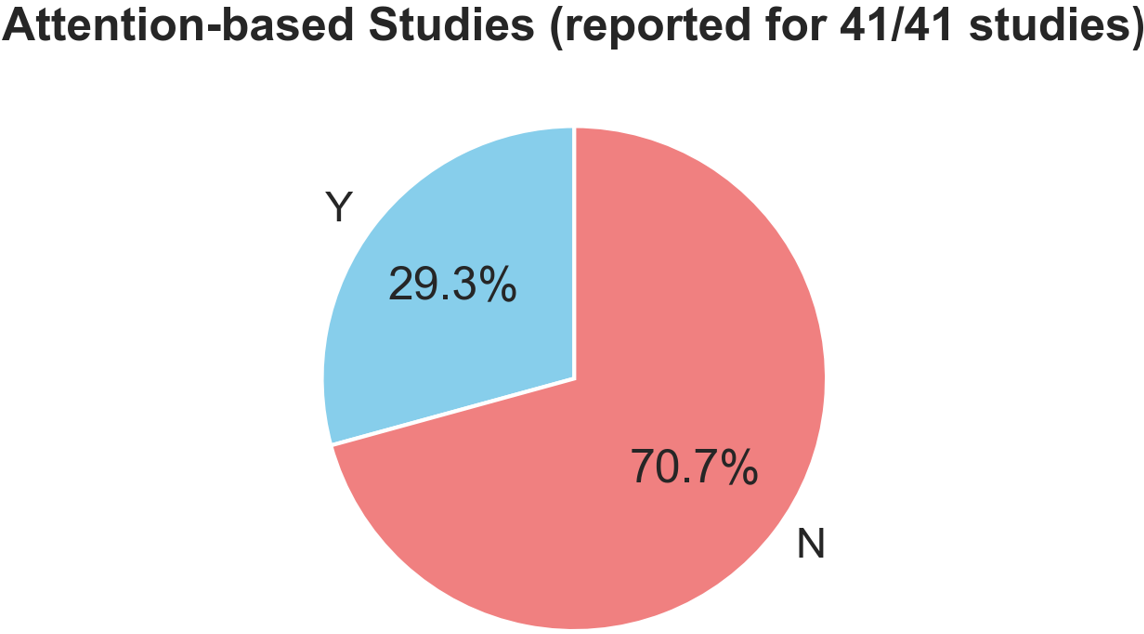


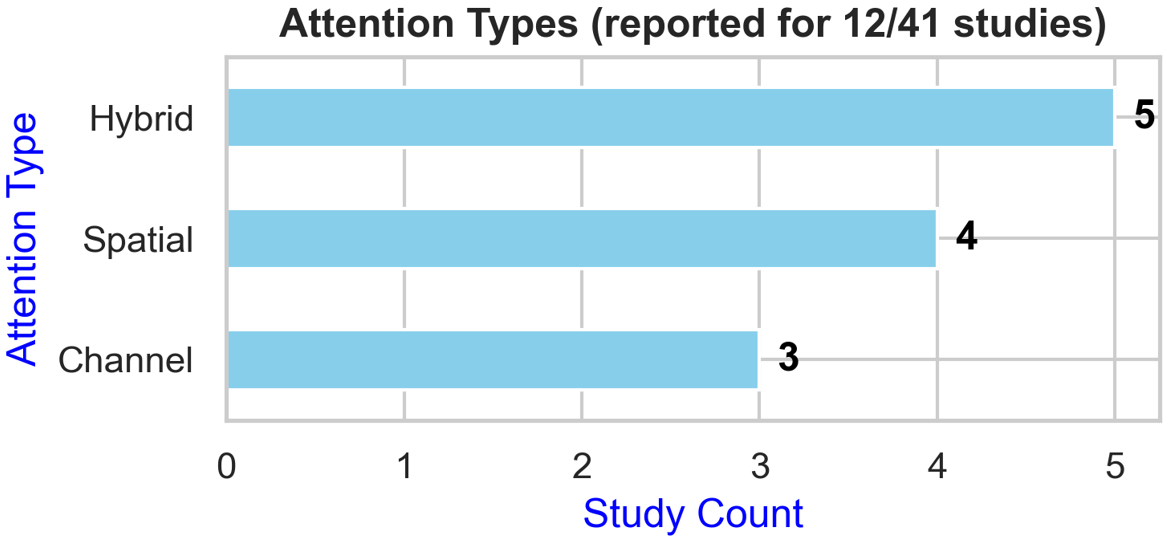


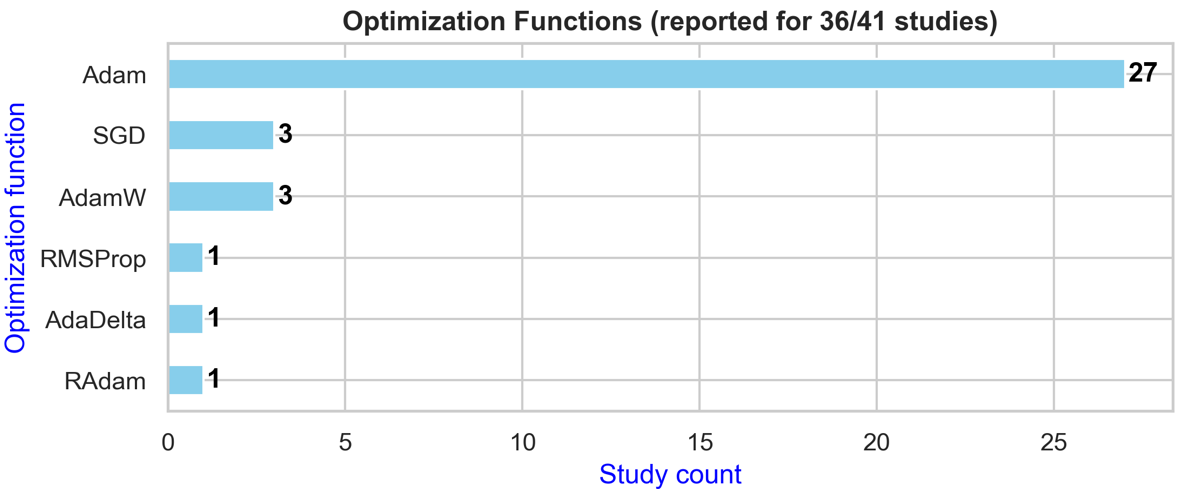


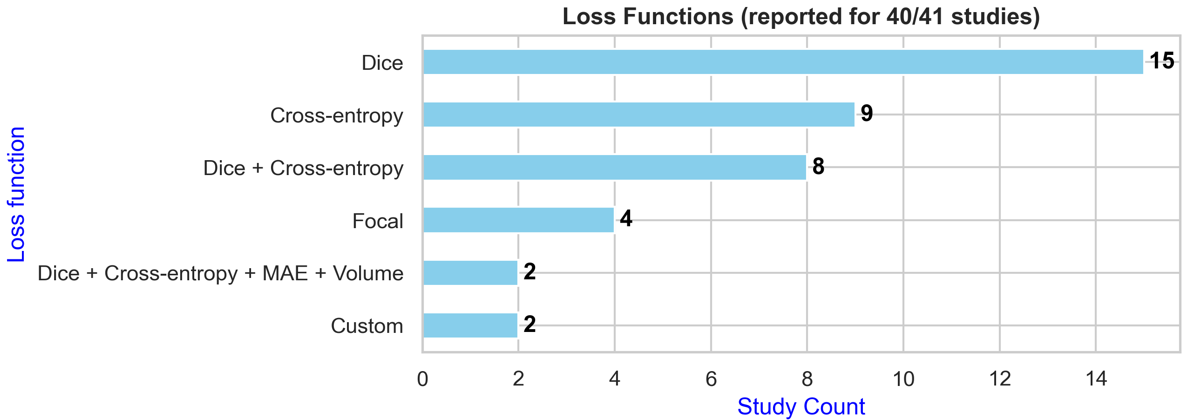


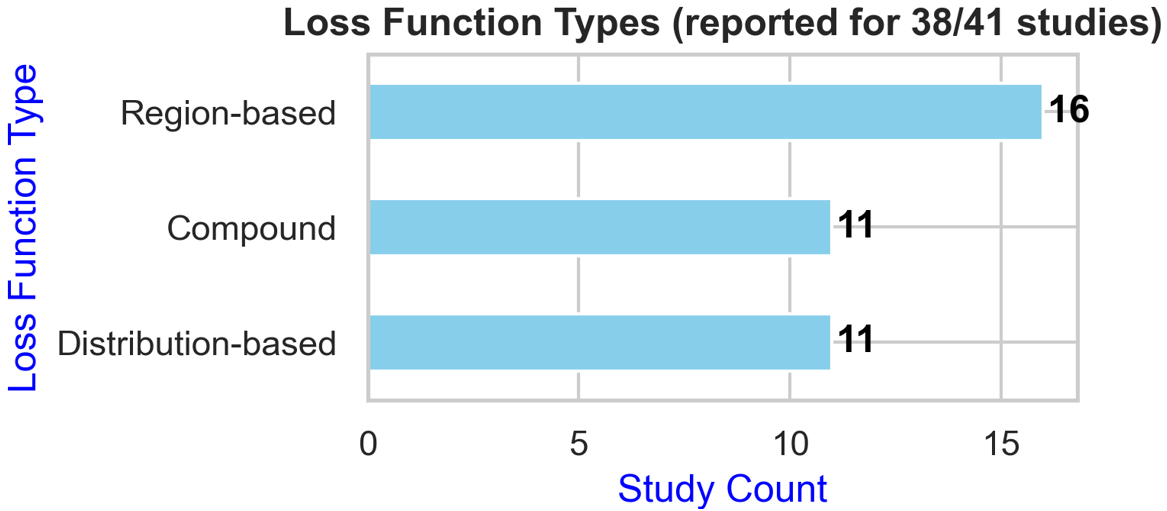


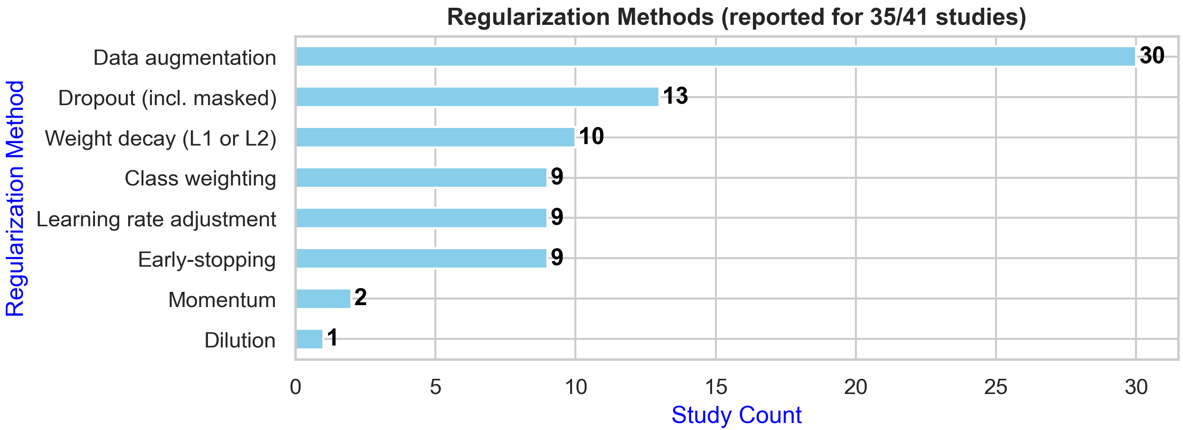


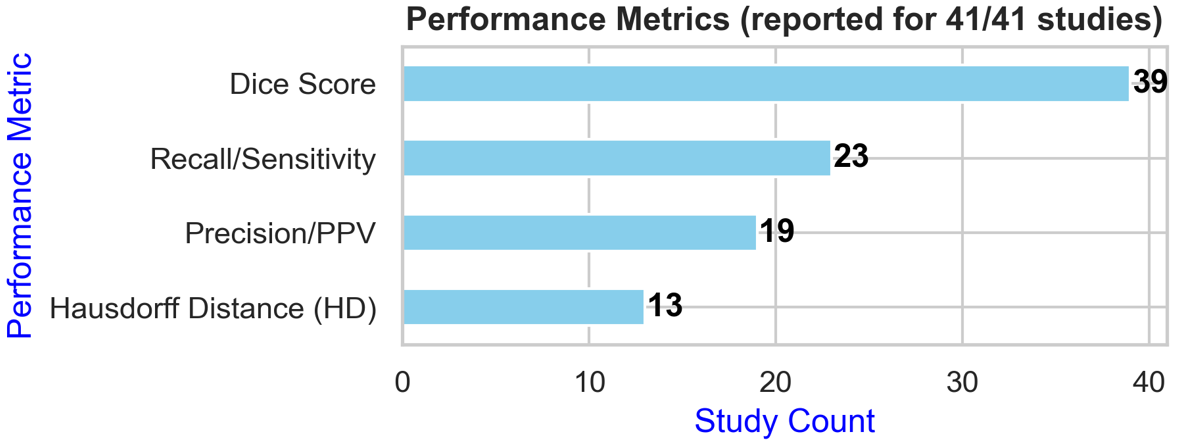


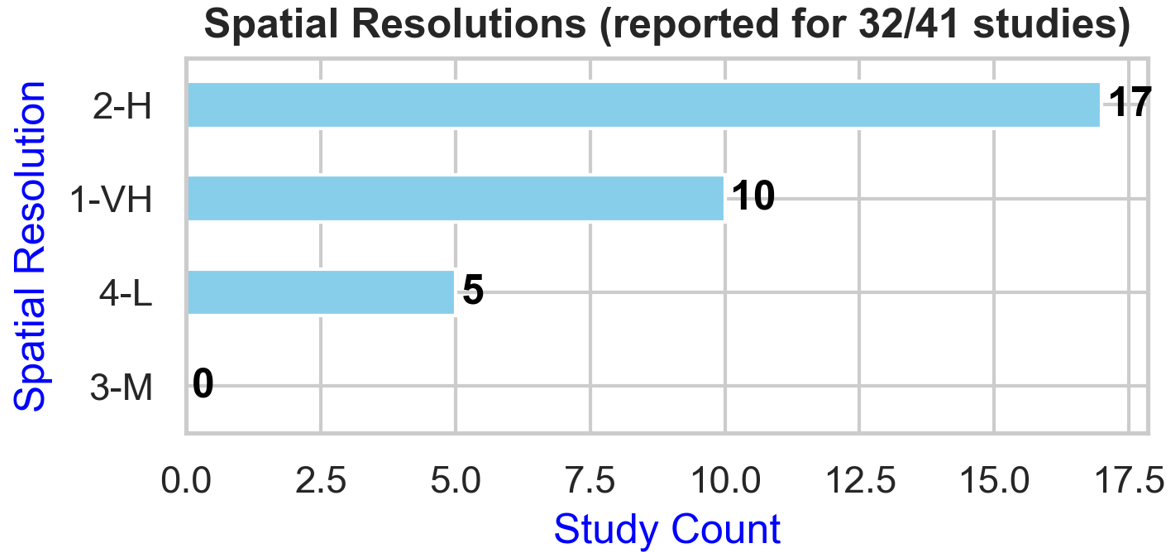


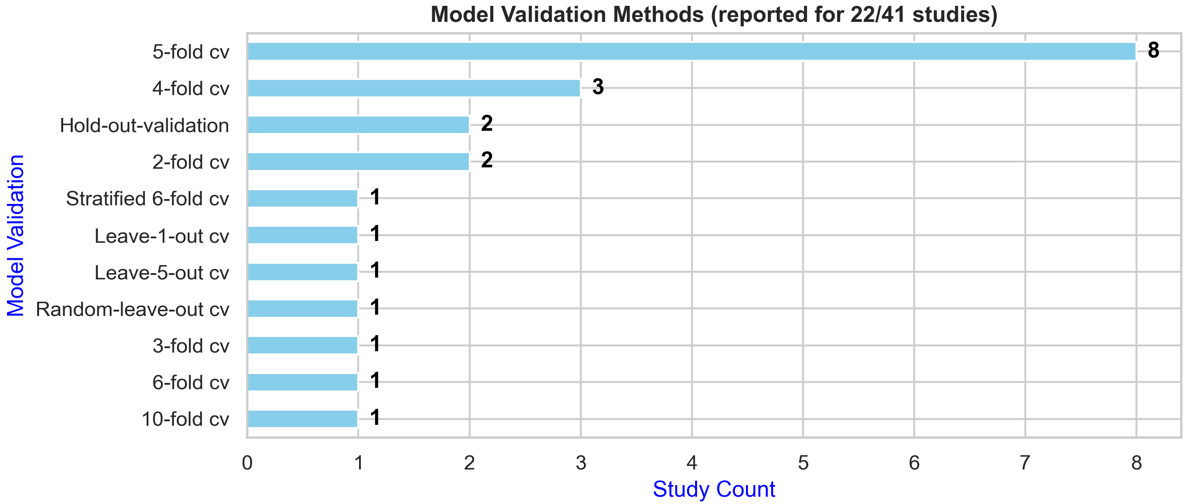


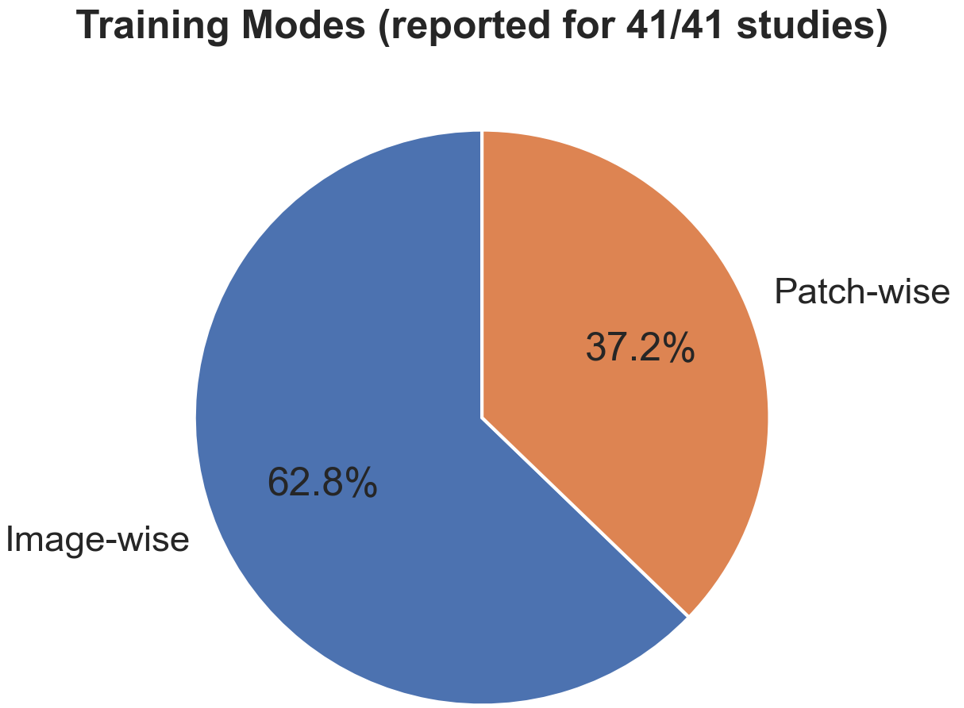


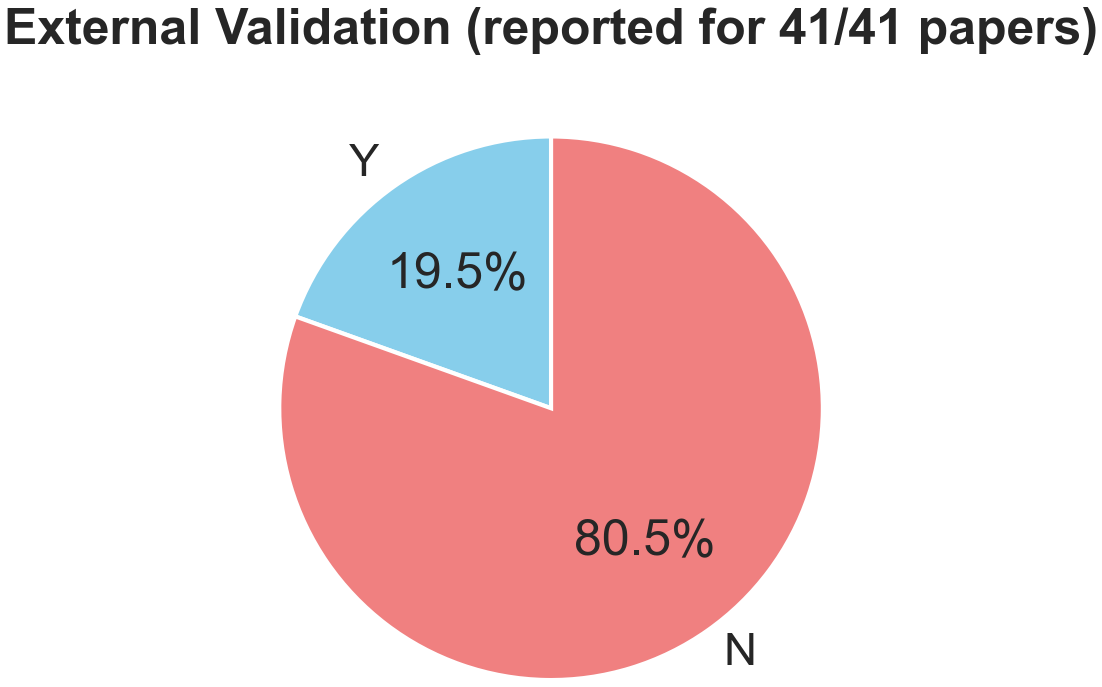


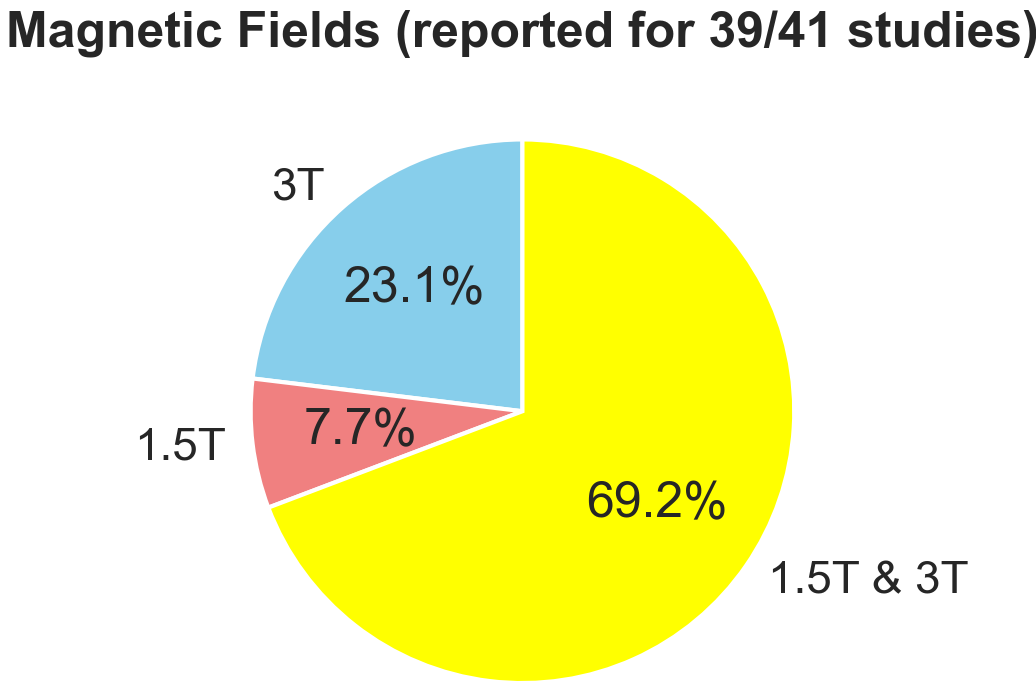


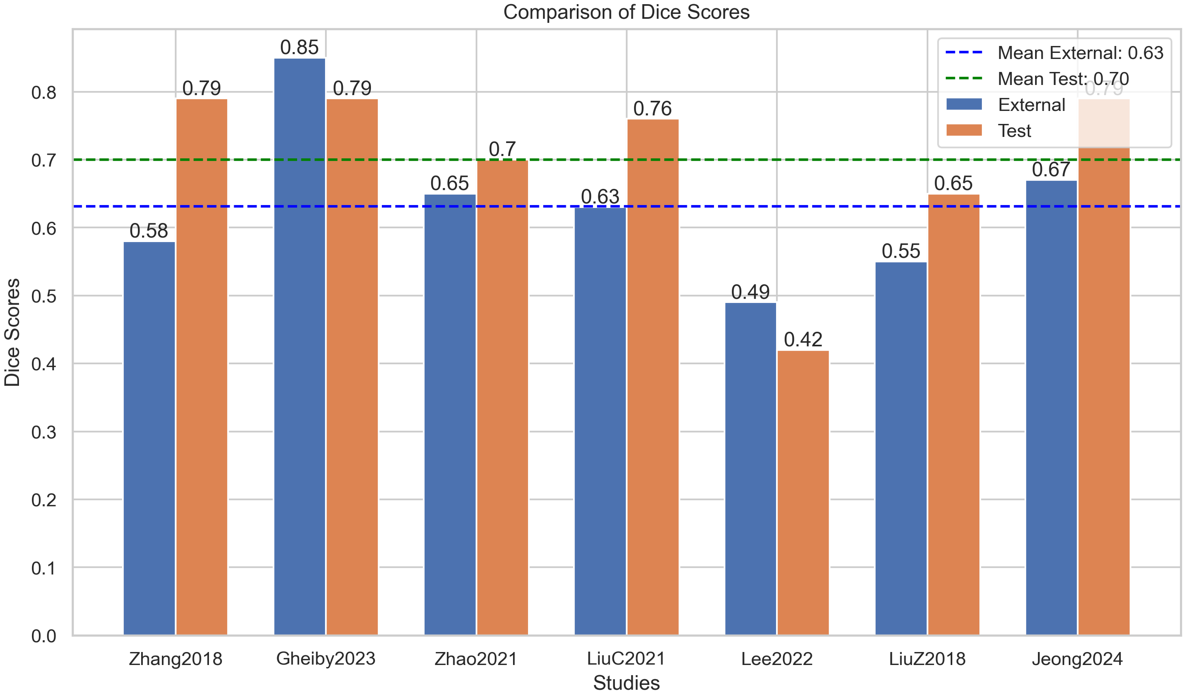


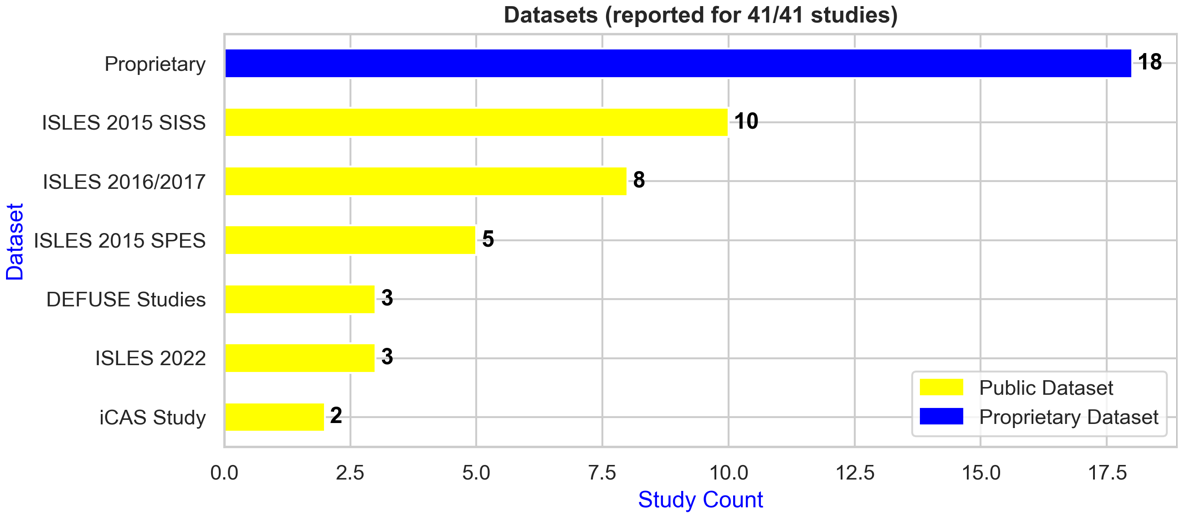


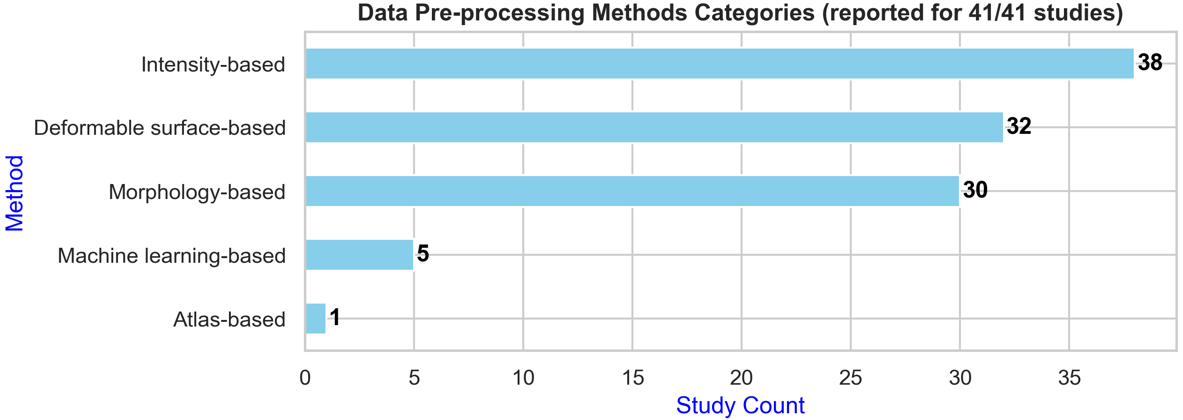


## 3. Correlation Analysis


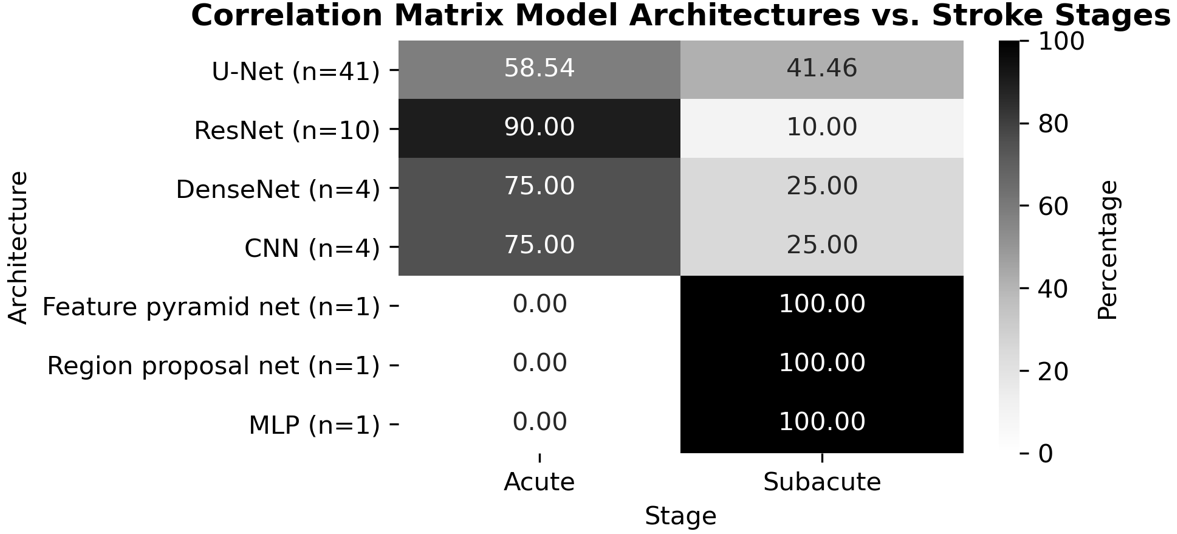


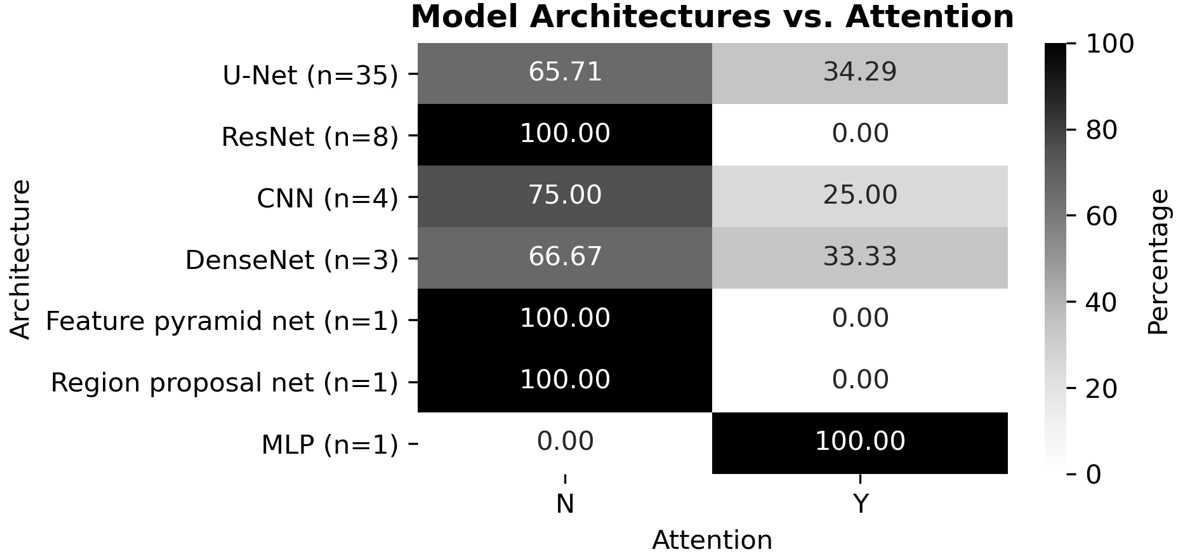


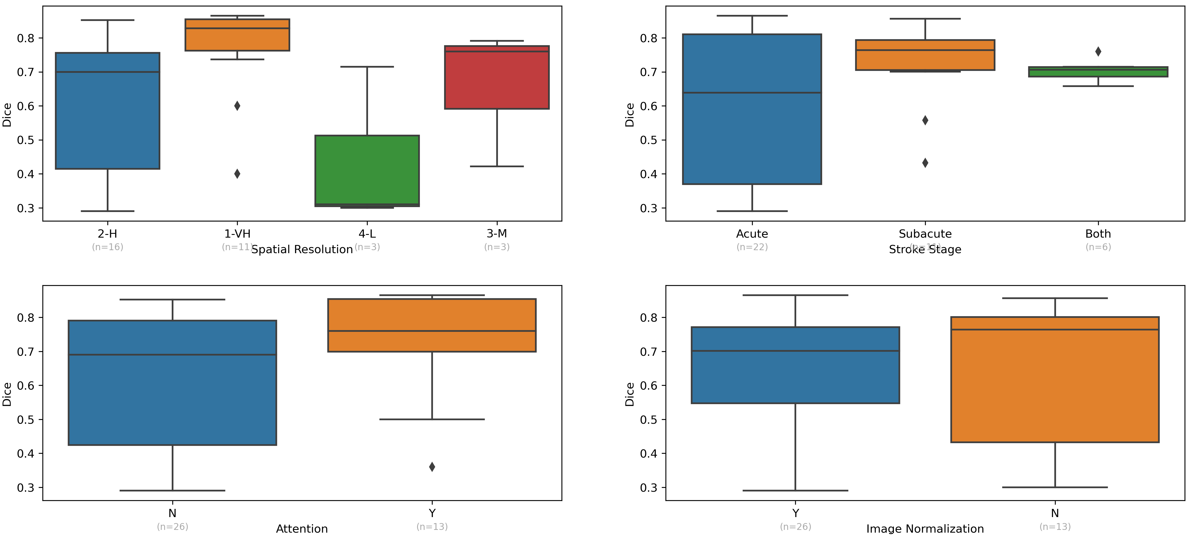


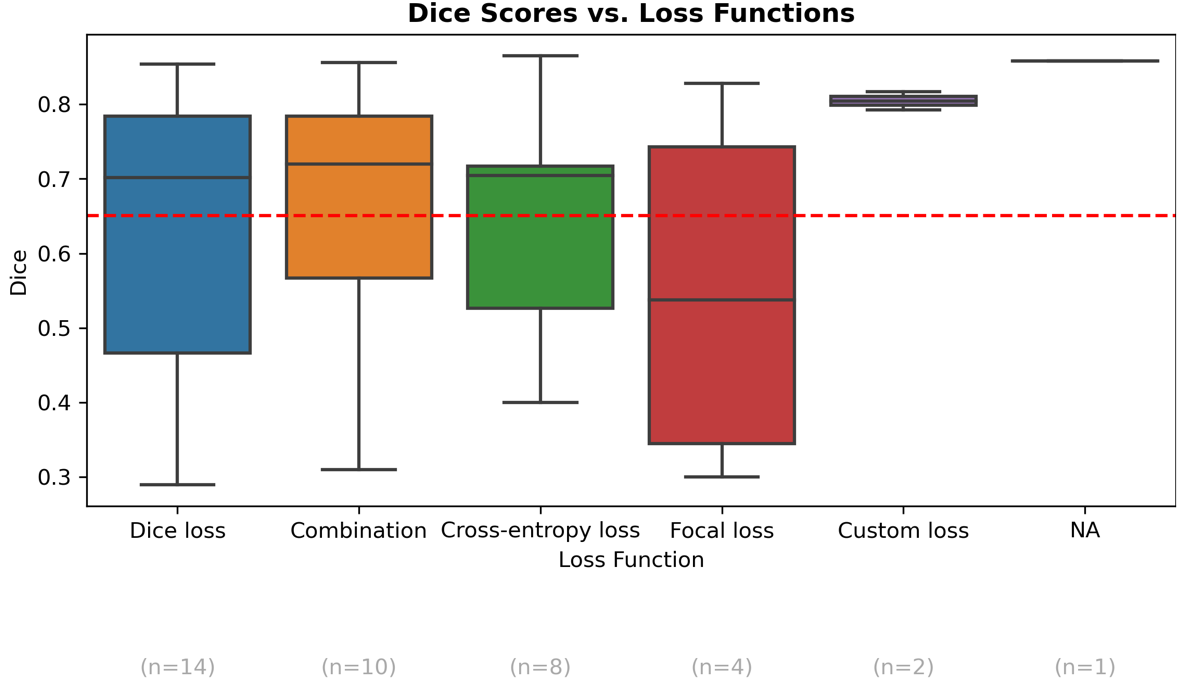


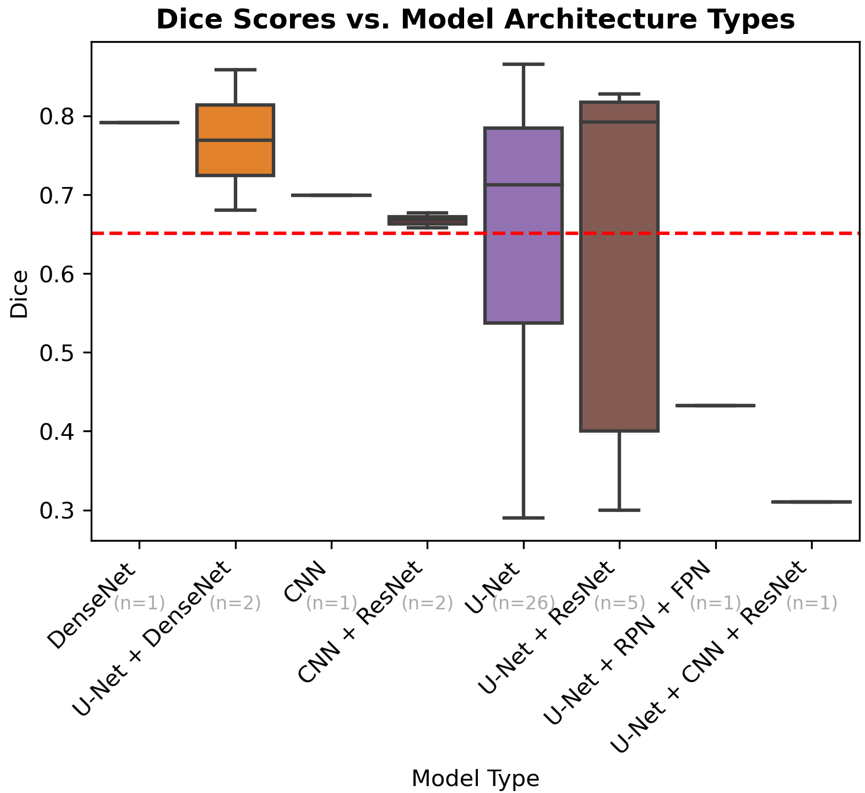


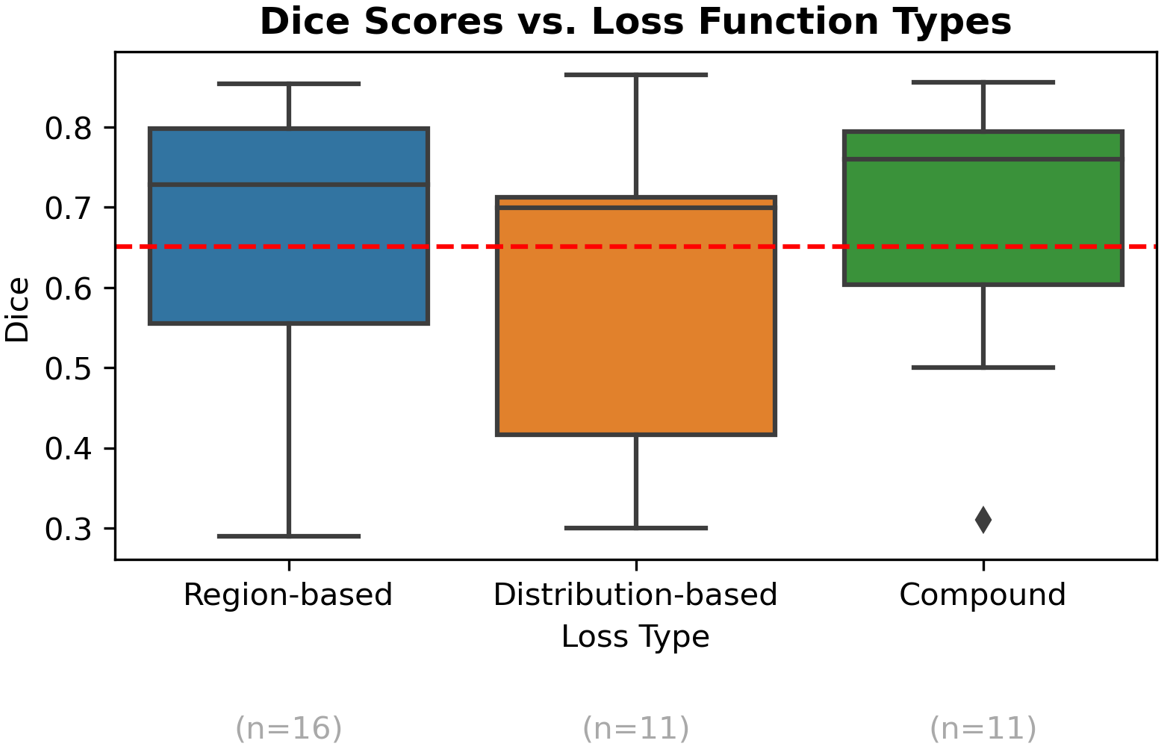


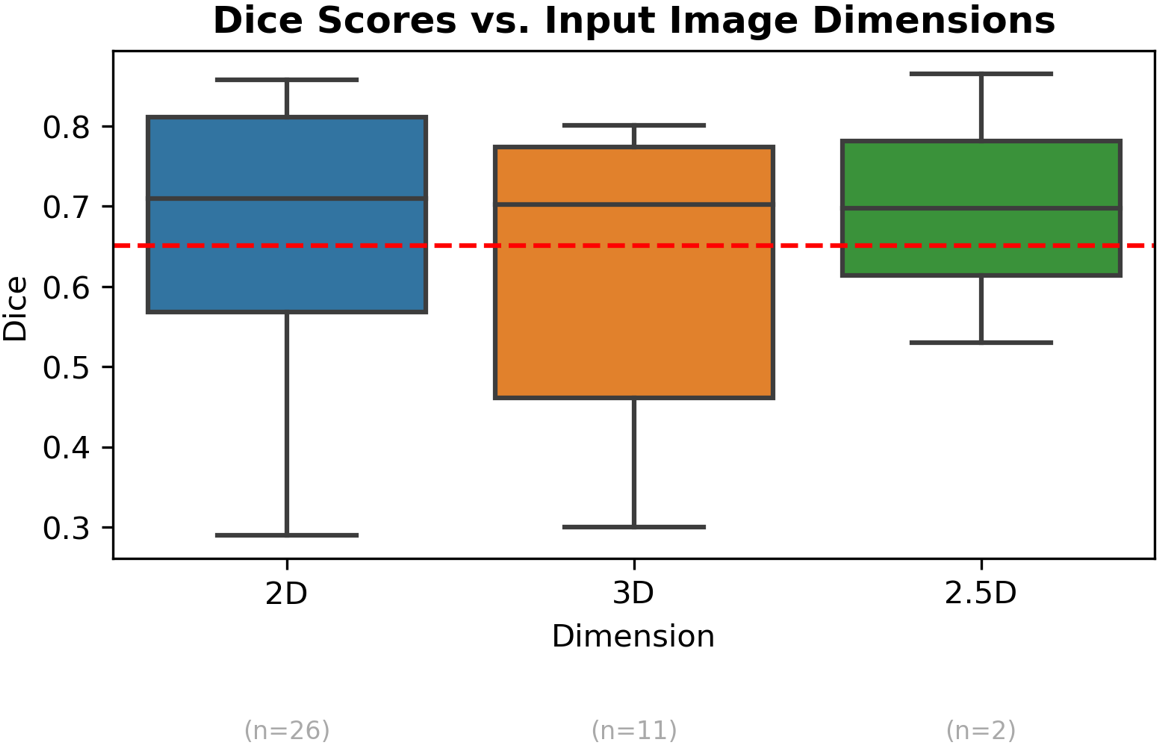


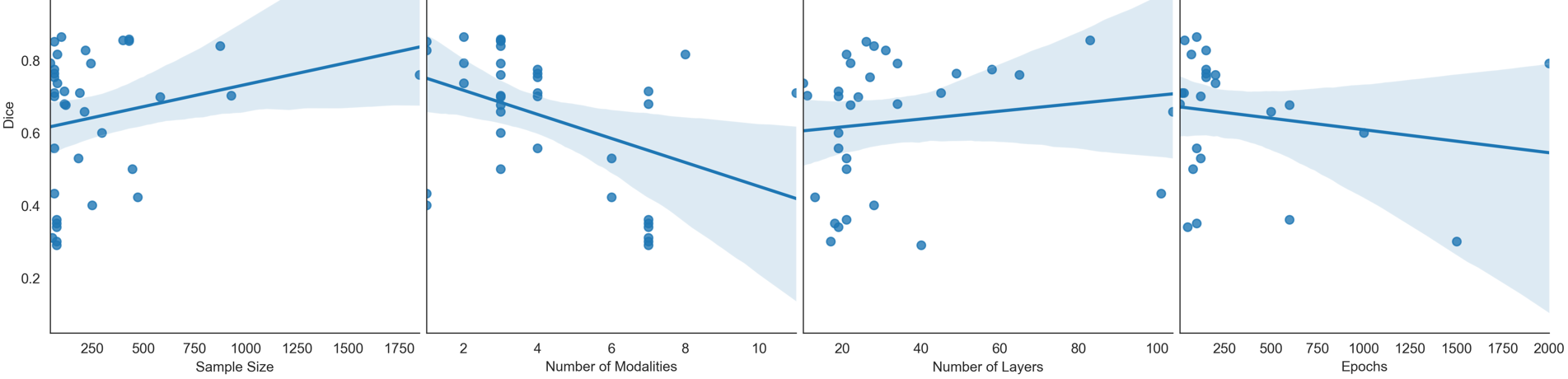


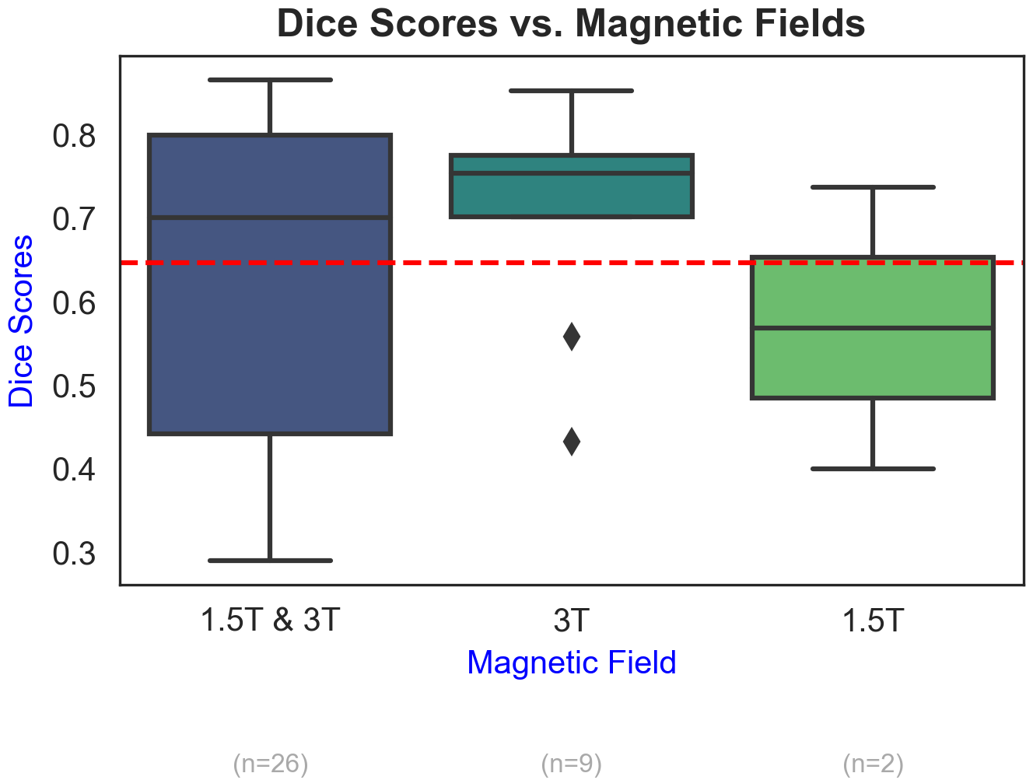


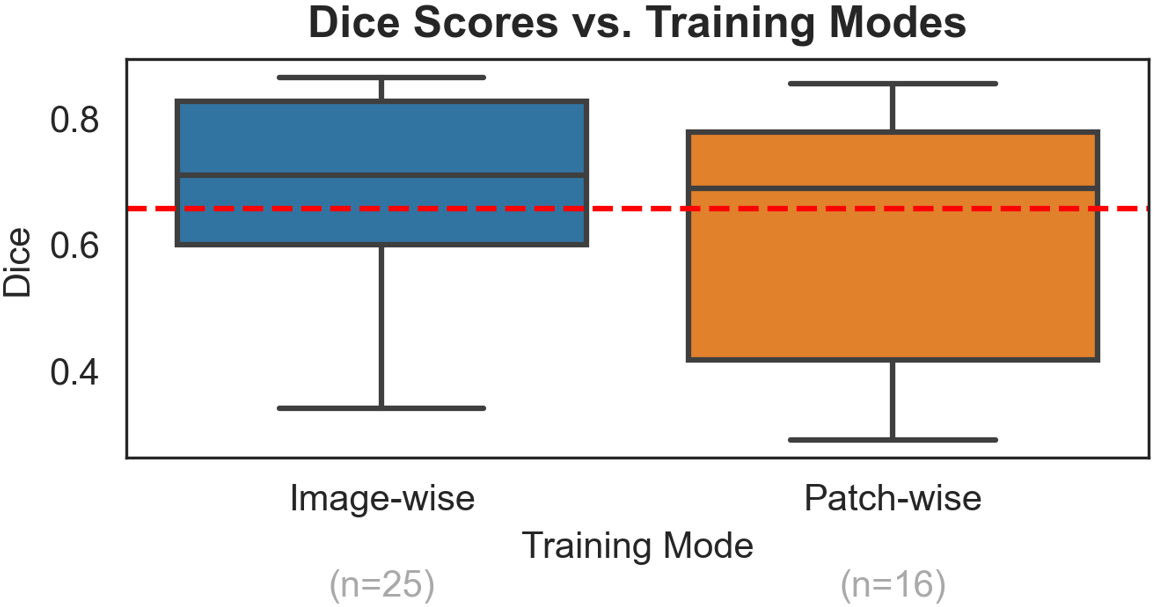


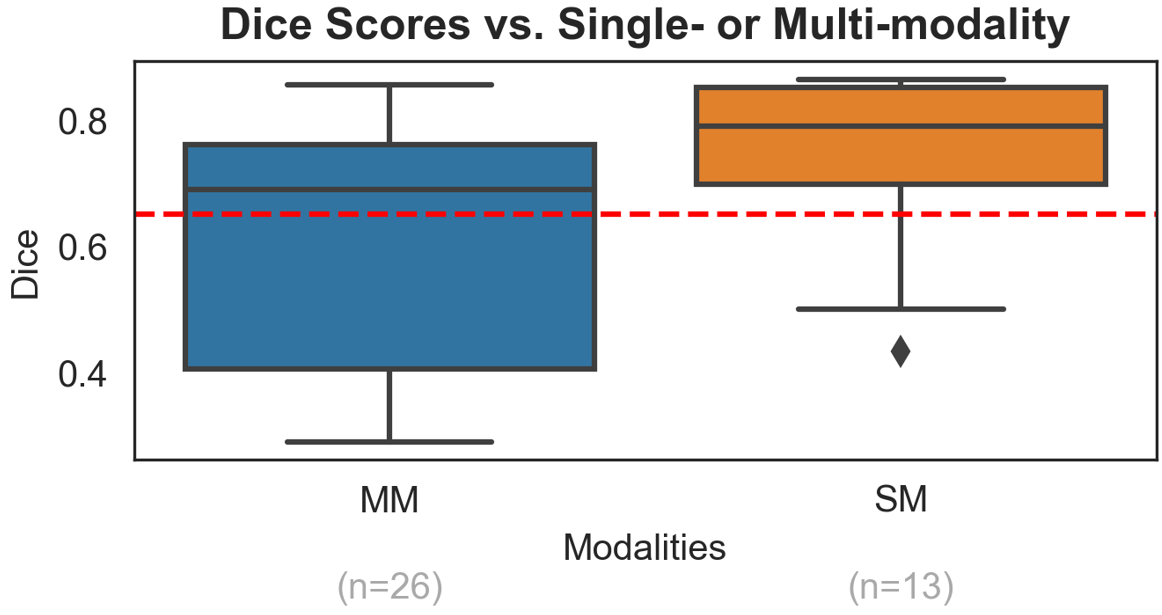


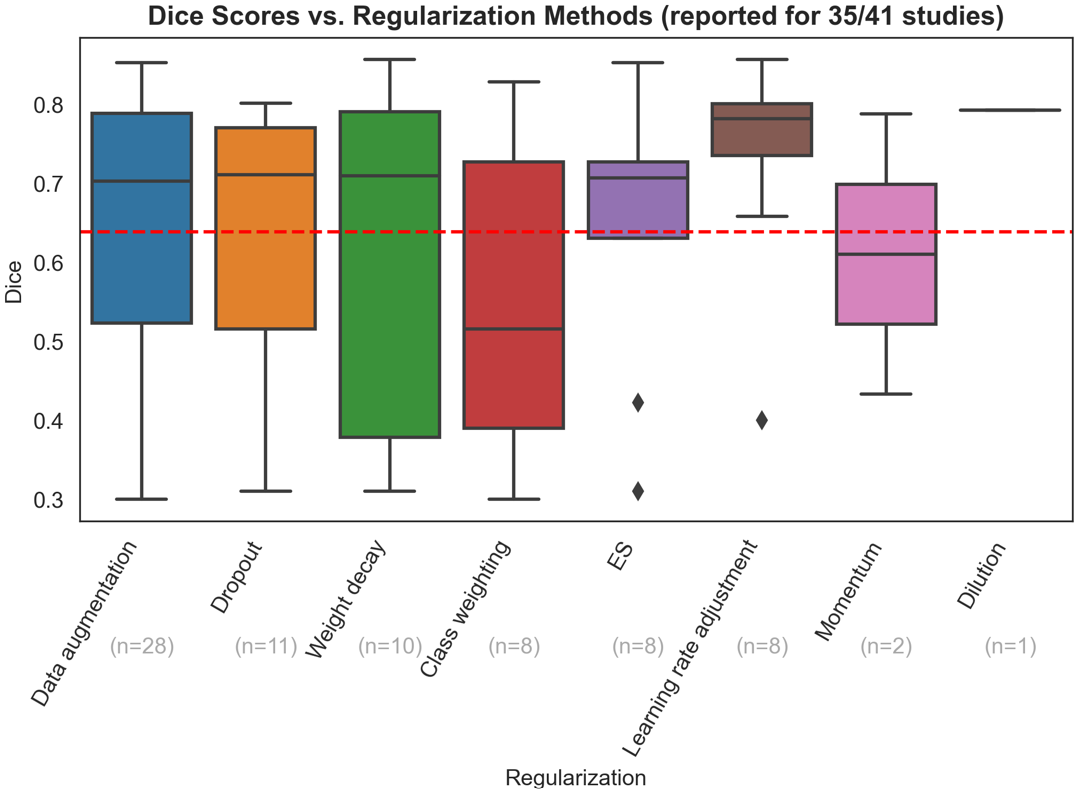


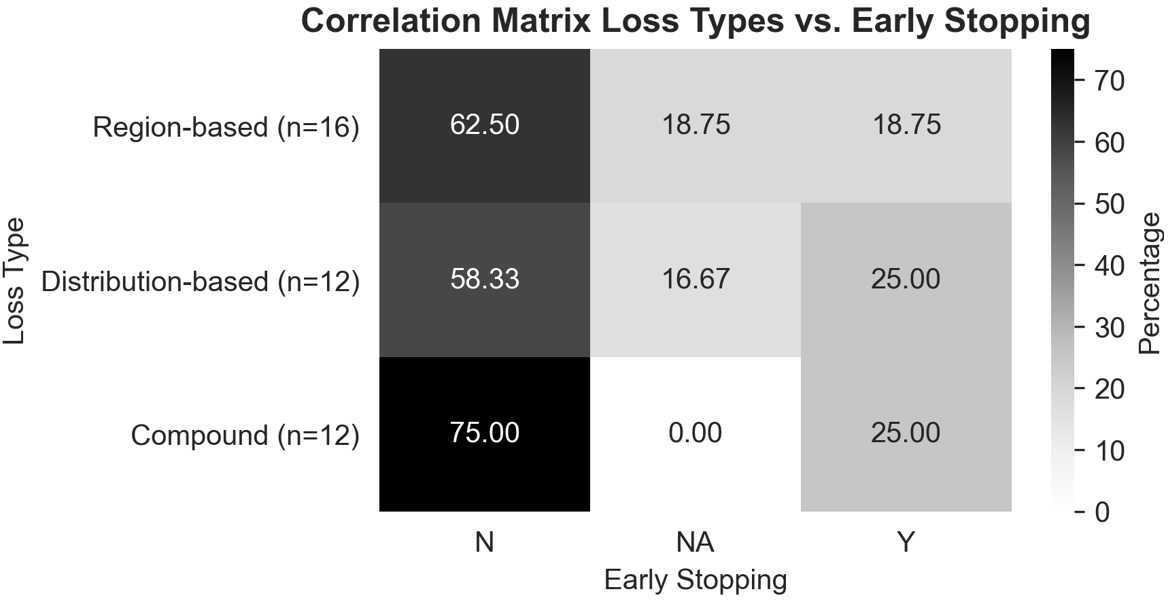


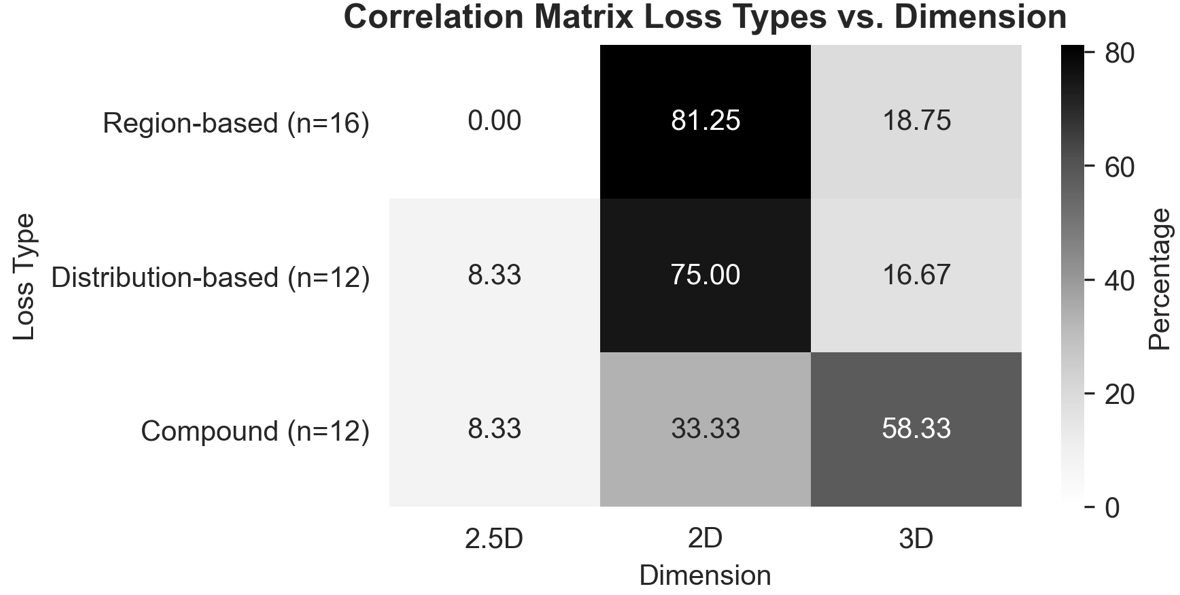


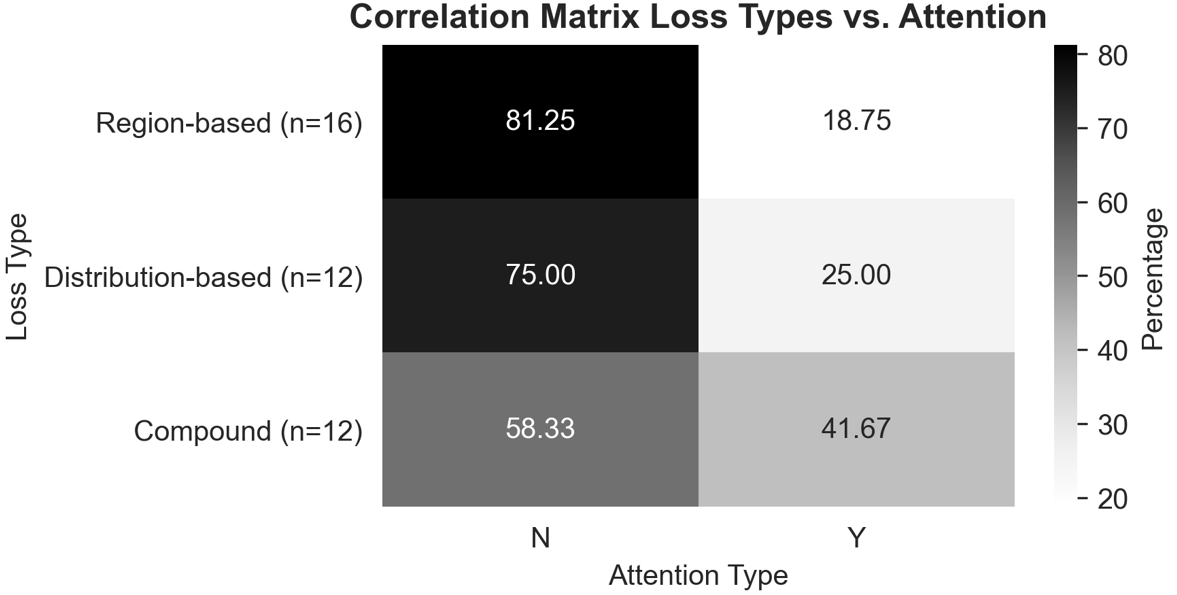


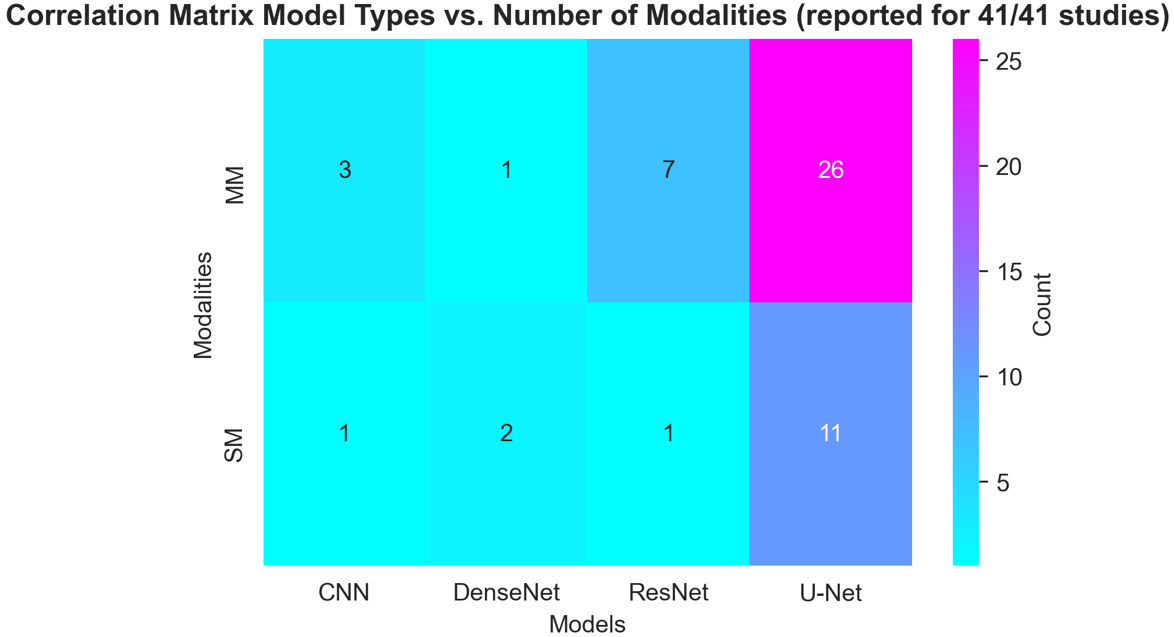


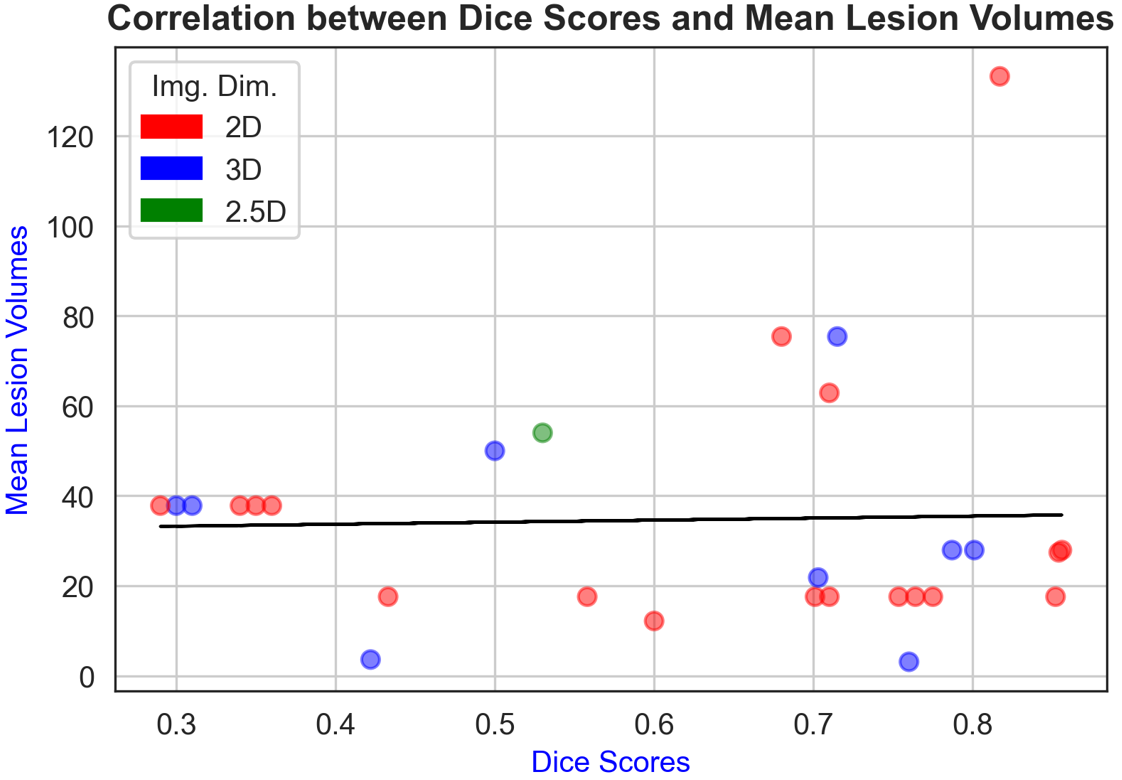


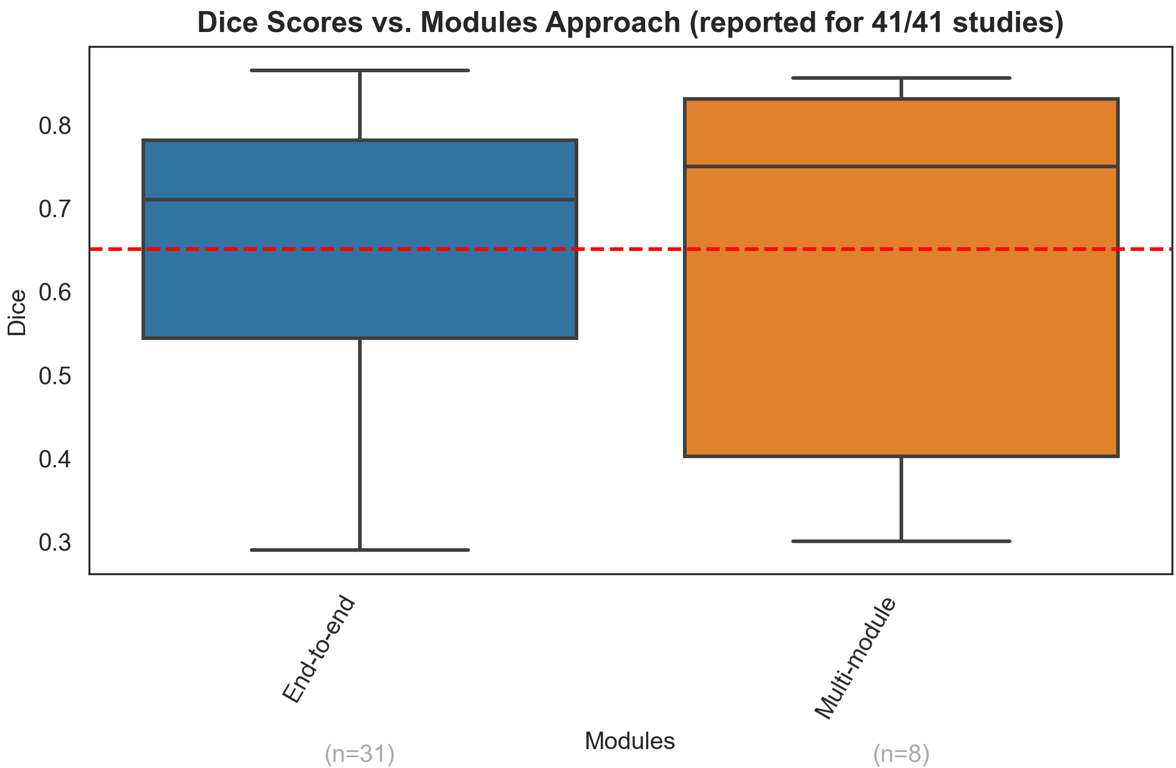


# **C - Full Data Extraction & Study QA Tables**

The attached excel file contains several worksheets:

- Worksheet 1: Data Extraction Table
- Worksheet 2: NIH’s Study Quality Assessment (QA) Table
- Worksheet 3: Heatmap of similar reviews

# **D - General Tips for Developers**

Here is a list of general tips for developers, based on the findings of our systematic review and pilot analysis:

- Computational cost of using dropout in larger models may outweigh its benefits. Also, when very few labelled training examples are available, dropout is less effective, and is outperformed by Bayesian neural networks
- Including skip connections is advisable for very deep networks (> 50 layers)
- Using ResNet architecture or multi-task learning can improve segmentation of small lesions. Network depth should depend on the size of input images (e.g., large image requires deeper network)
- For fine-tuning the learning rate, it is advised to monitor the first several iterations and use a learning rate that is slightly higher than the best-performing learning rate during these first iterations
- Combining Dice loss with boundary-based loss may solve the issue of overlooking precise contour details
- Distribution-based loss functions (e.g., CE loss) are useful when dealing with small diffuse lesions
- 3D models are better suited to handle the complexity of compound loss optimization than 2D models
- Since 2D models treat each slice as a complete input, good performance would depend on whether the spatial context within individual slices is sufficient for accurate segmentation
- Adam is considered fairly robust to the choice of hyperparameters, but it is preferable to use it with learning rate adjustment since Adam optimization introduces noise due to random sampling of training examples, and that noise is not removed even when we arrive at a minimum
- With ES, one must choose a small validation set relatively to the training set (5-10%) for computational efficiency
- Using ES with compound loss is not advisable, as interactions between different loss components in compound loss functions may lead to non-monotonic loss curves or fluctuating gradients during training, and ES may not effectively capture the true convergence state of the model or may halt training before optimal convergence
- It is advised to use dilated convolution blocks in semantic segmentation tasks, as they are able to enlarge the receptive field without pooling operations, hence mitigating feature loss during down-sampling operations
- Dice is an overlap metric, which must be accompanied by surface metrics (e.g., HD), boundary metrics (e.g., Boundary Displacement Error) or volumetric metrics (e.g., Mean Absolute Error) for a more holistic evaluation
- Dice counts both false positives and missed values in each class, which makes it useful for assessing overall pixel accuracy. However, it does not express how well the algorithm identified the true stroke lesions and/or excluded the non-stroke lesions
- Measures such as accuracy and specificity tend to be avoided in AIS lesion segmentation tasks, since they do not discriminate between different segmentation outputs when the lesion is smaller than the background
- Combining both mismatch analyses (i.e., DWI-PWI and DWI-FLAIR) can help effectively delineate stroke lesions
- Quantifying uncertainty can facilitate the clinical use of the algorithm
- Data augmentation aim to reduce overfitting
- 3T magnetic field strength can also help with small lesions
