# Supplementary material for: Deep learning for MRI-based acute and subacute ischaemic stroke lesion segmentation—a systematic review, meta-analysis, and pilot evaluation of key results
Source: Front Med Technol. 2025 Jun 10;7:1491197. doi: 10.3389/fmedt.2025.1491197 (PMC12185483; doi:10.3389/fmedt.2025.1491197)
Supplement: Supplementary Data Sheet 2 — Supplementary Figures. [file Datasheet2.docx]

**Deep Learning for MRI-based Acute and Subacute Ischaemic Stroke Lesion Segmentation - A Systematic Review, Meta-Analysis, and Pilot Evaluation of Key Results**

- **SUPPLEMENTARY FIGURES AND TABLES –**


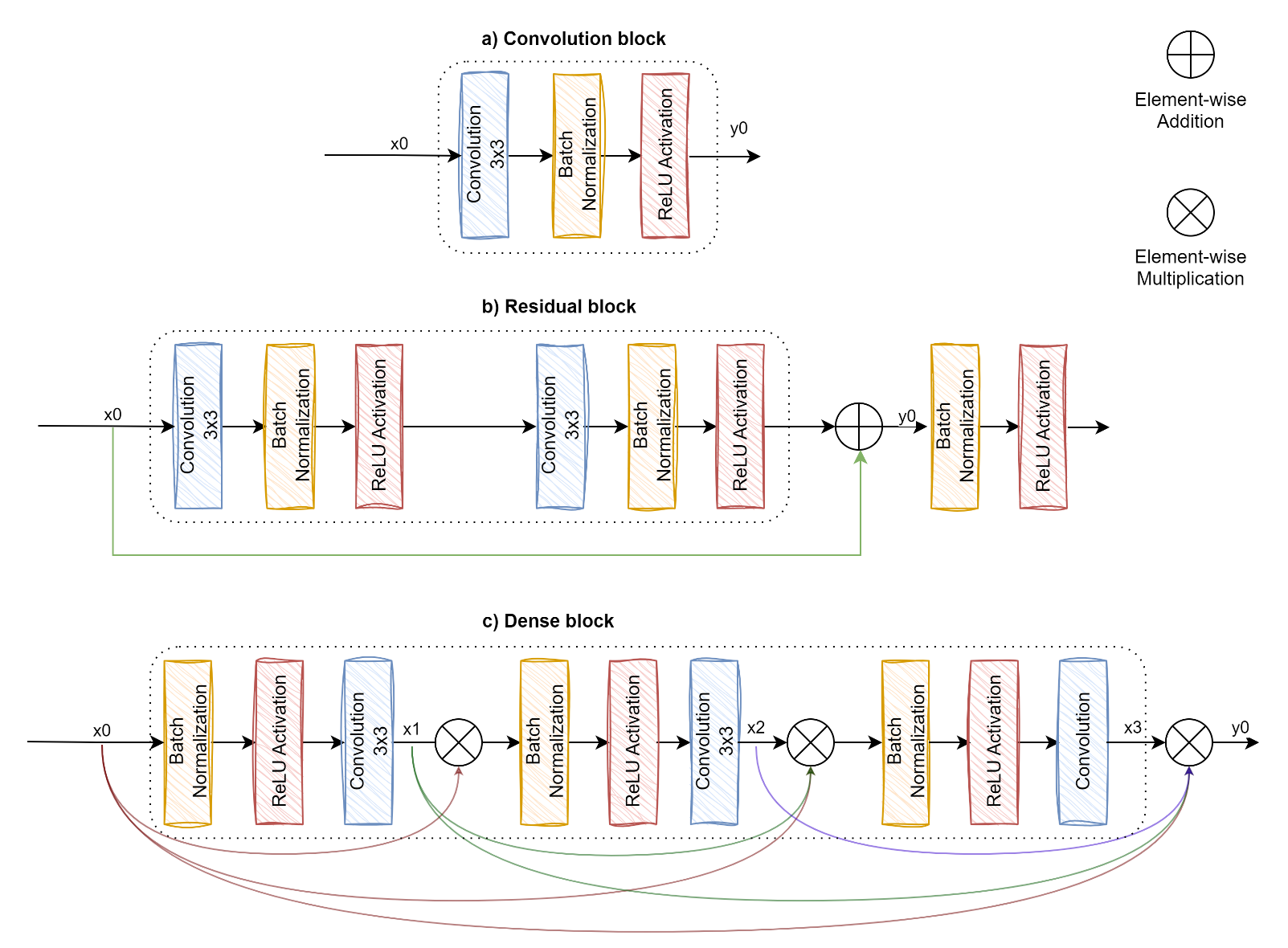


**Supplementary Figure 1.** Architecture of the main types of blocks found in deep learning architectures. **a) Standard convolutional block** (typically included in CNNs): illustrated consisting of a convolution operation on a 3x3 kernel, which produces a feature map on which batch normalization is applied, before passing through a ReLU activation function to introduce non-linearity. **b) Standard residual block** (typically includes in ResNets): consists of an “identity path” (green arrow) that can bypass the “residual path”, thus giving the network the option to simply copy activations from layer to layer and preserve information when learned features do not require more depth. The residual path represented has 2 consecutive convolution blocks. When the identity of the residual path is taken, batch normalisation and ReLU activation are applied before passing to the next network block. **c) Standard dense block** (typically included in DenseNets): employs a radical dense connection strategy, interconnecting all layers to maximize information and gradient propagation, as seen with the green, red and blue arrows. It is represented consisting of 3 consecutive convolution blocks with a slightly different sequence of operations (i.e., batch normalisation, then ReLU, then the convolution operation).


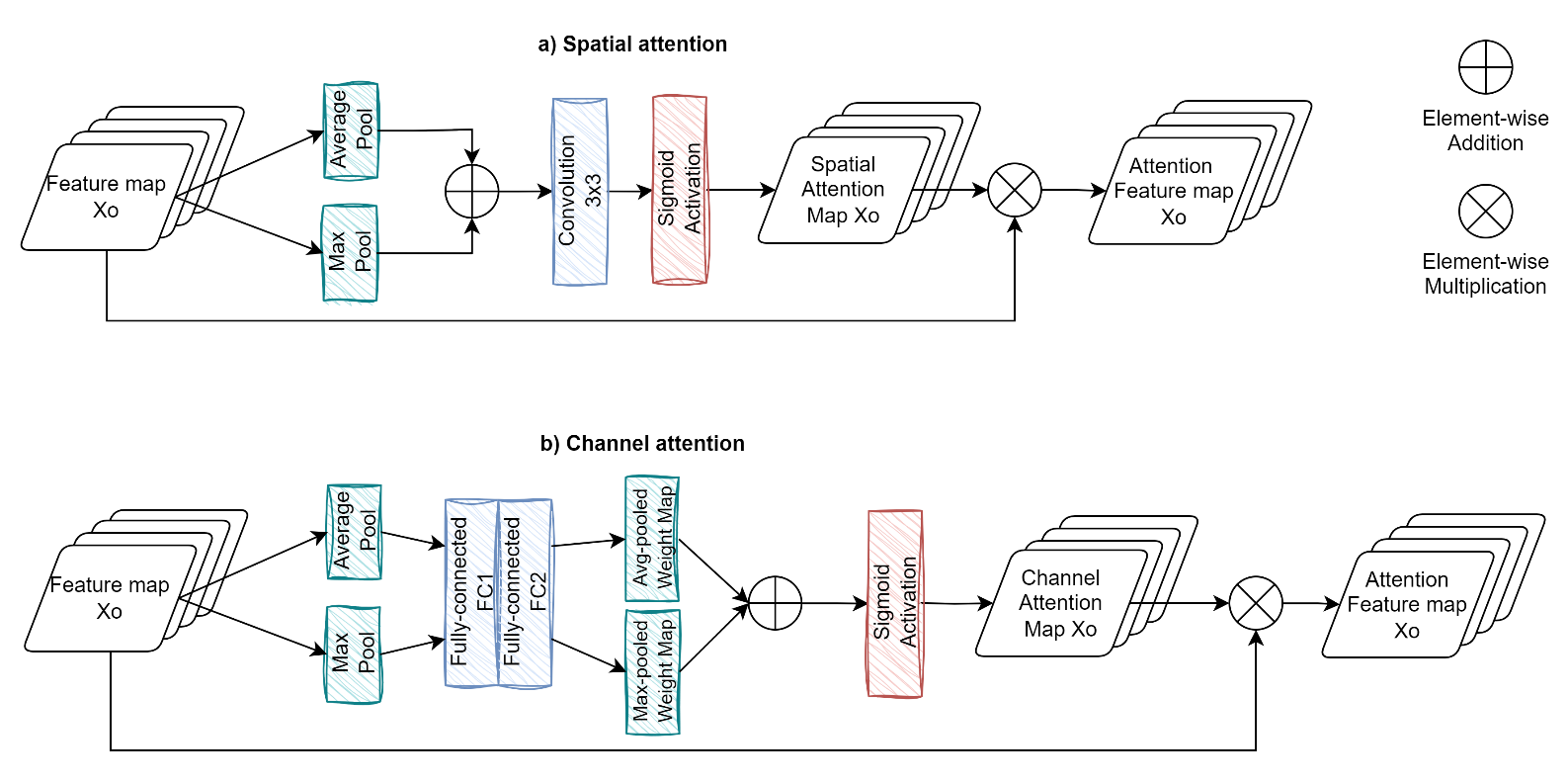


**Supplementary Figure 2.** Architecture of common attention mechanism blocks that may be incorporated in deep learning networks. **a) Architecture of a standard spatial attention block**: the feature map first goes through a max-pooling and an average pooling operation, in parallel. The outputs of the two pooling blocks are then concatenated using element-wise addition. A 3x3 convolution kernel followed by a sigmoid activation are then applied in order to produce a binary spatial attention map, where only supposedly important pixels are in the foreground, and supposedly unimportant ones are in the background. The original feature map and the spatial attention map are then combined using element-wise multiplication, in order to produce the final attention-enhanced feature map that is passed to the next layer of the network. **b) Architecture of a standard channel attention block**: the feature map first goes through a max-pooling and an average pooling operation, in parallel. Each output goes through two consecutive fully-connected layers, in order to produce an average-pooled weight map and a max-pooled weight map. Both maps are, then, concatenated using element-wise addition, before going through the sigmoid activation to produce the channel attention weight map, which either classifies a feature map as unimportant for learning (Wo=0) or important for learning (Wo=1). The original feature map and the channel attention weight map are concatenated using element-wise multiplication in order to produce the final attention-enhanced feature map that is passed to the next layer of the network.


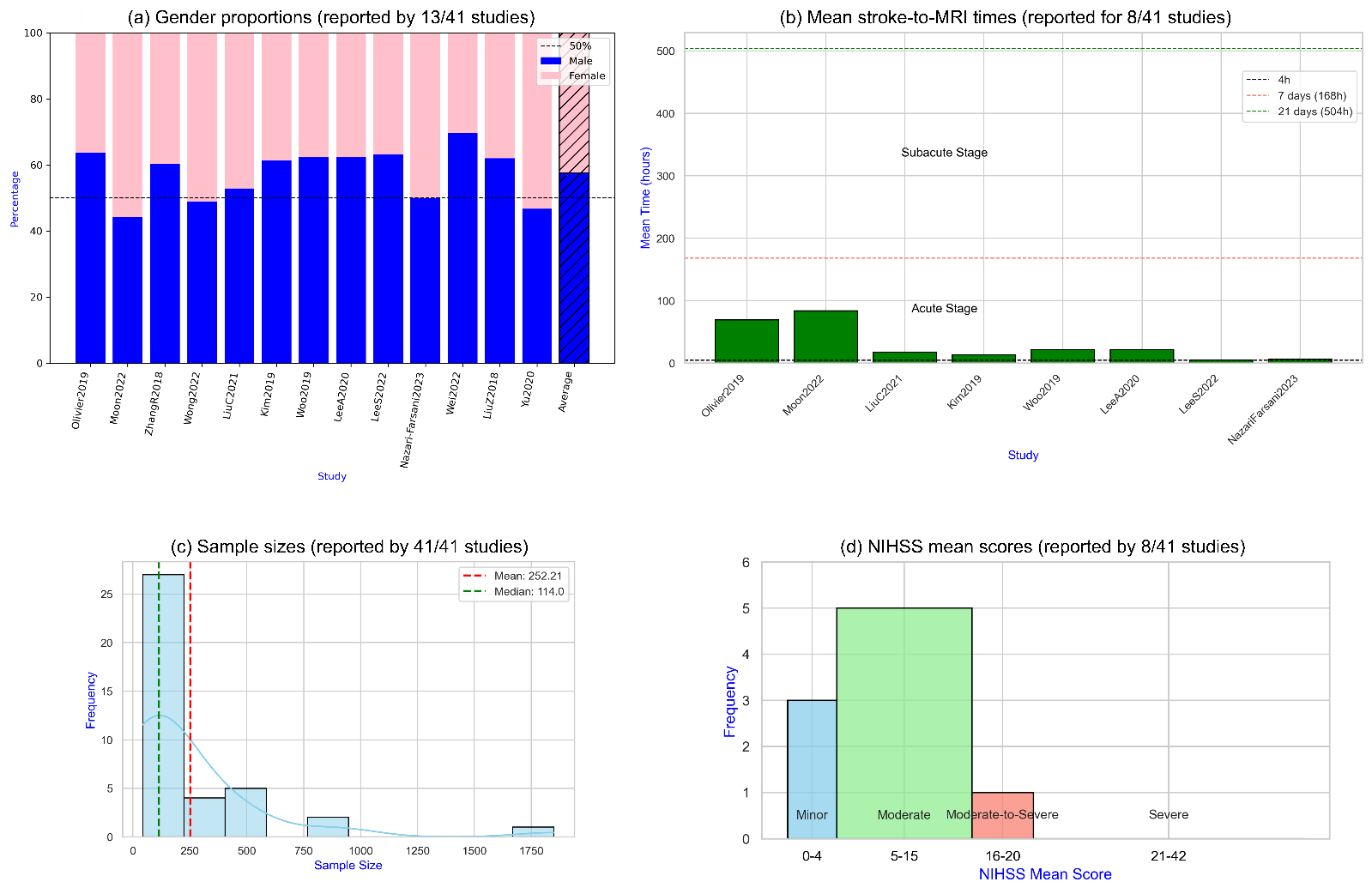


**Supplementary Figure 3. a) Gender proportions** (Male/Female) per study; **b) Mean stroke-to-MRI times** (in hours), along with associated stage of stroke; **c) Sample sizes** along with mean and median; **d) NIHSS mean scores**, along with associated stroke severity category, as defined by NIHSS.

**
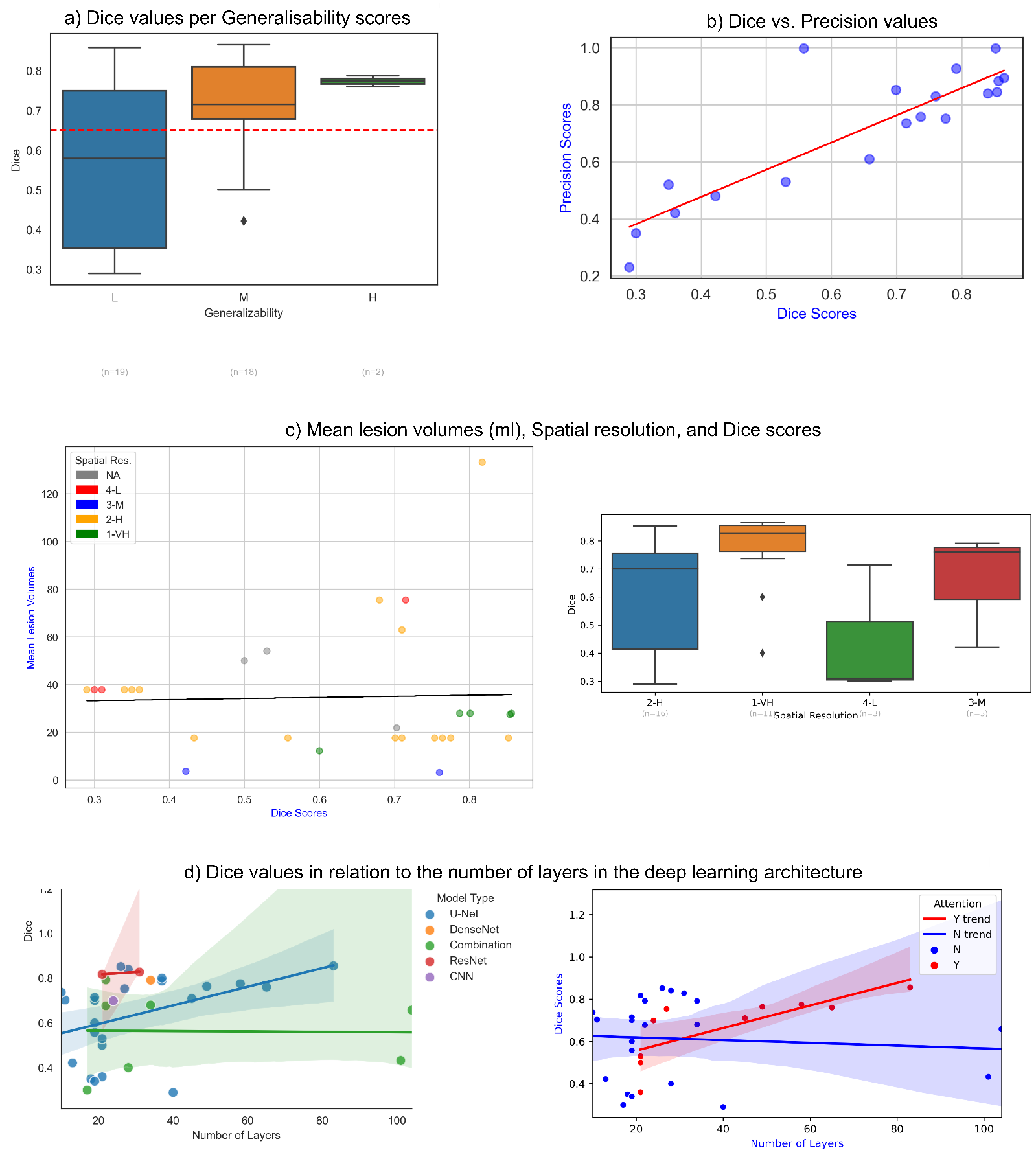
**

**Supplementary Figure 4.** Comparative analyses of the performance of the models presented in the papers reviewed. **a)** Correlation between the Dice scores (numerical value between 0-1) and the Generalisability scores (High/Medium/Low) obtained per paper; **b)** Correlation between the Dice scores and the Precision scores (numerical value between 0-1) obtained per paper; **c)** Correlation between the Mean lesion volumes reported (numerical value in ml) and the Dice scores obtained per paper (left panel) & Correlation between the Dice scores and the Spatial resolution of the images used as input to the proposed models (i.e., 1-Very High, 2-High, 3-Medium, 4-Low) (right panel); **d)** Correlation between the Dice scores obtained and the Number of layers used in the proposed model architectures using a filter on Model types (i.e., U-Net, ResNet, DenseNet, CNN, Combination) (left panel) & Correlation between the Dice scores obtained and the Number of layers used in the proposed model architectures using a filter on whether Attention mechanisms were used (Yes: Red) or not (No: Blue) (right panel).


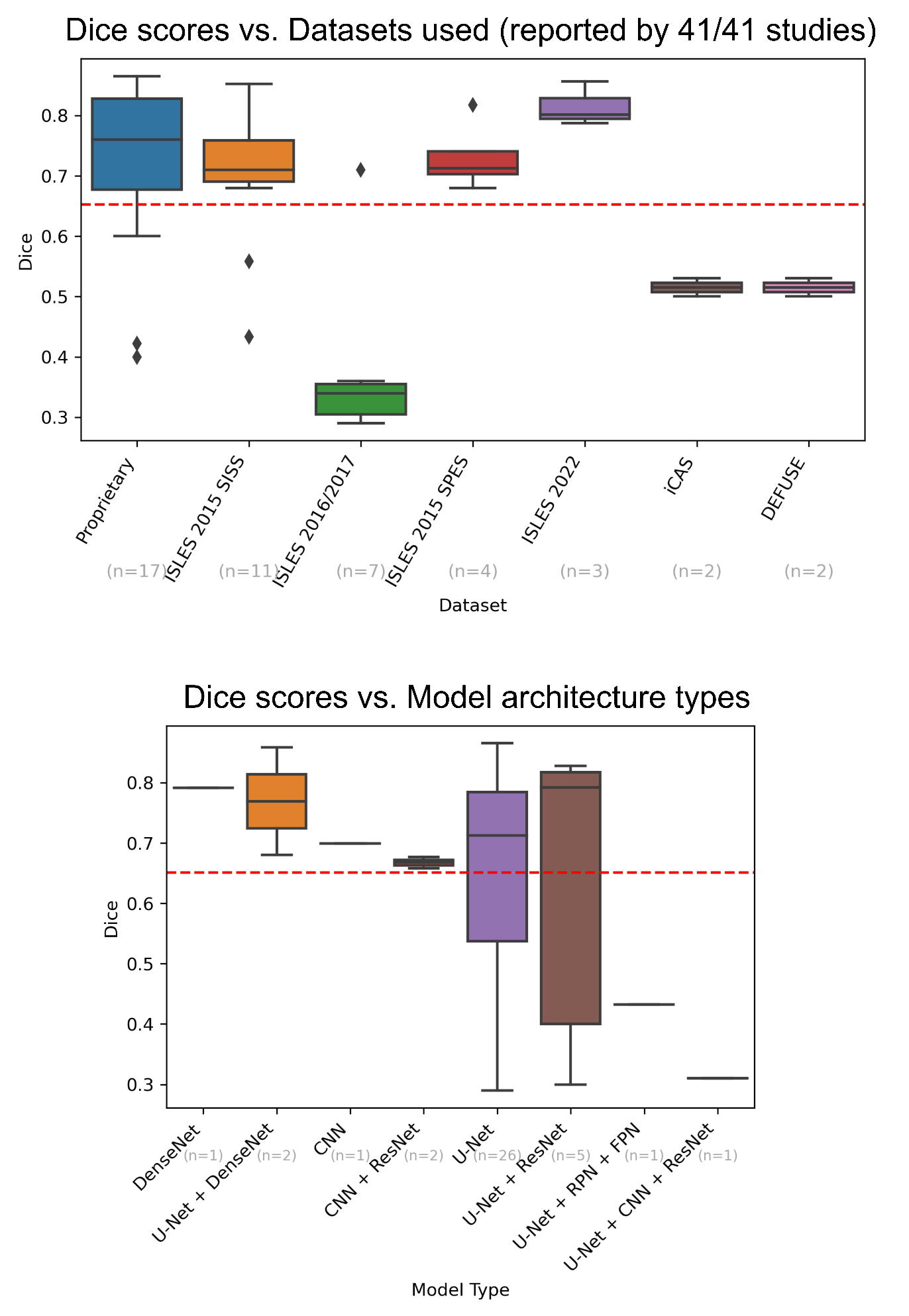


**Supplementary Figure 5.** Comparative analyses of the performance (in terms of Dice Similarity Coefficient values) of the models reviewed in terms of **a)** Datasets used (panel above) and **b)** network configuration (panel below).


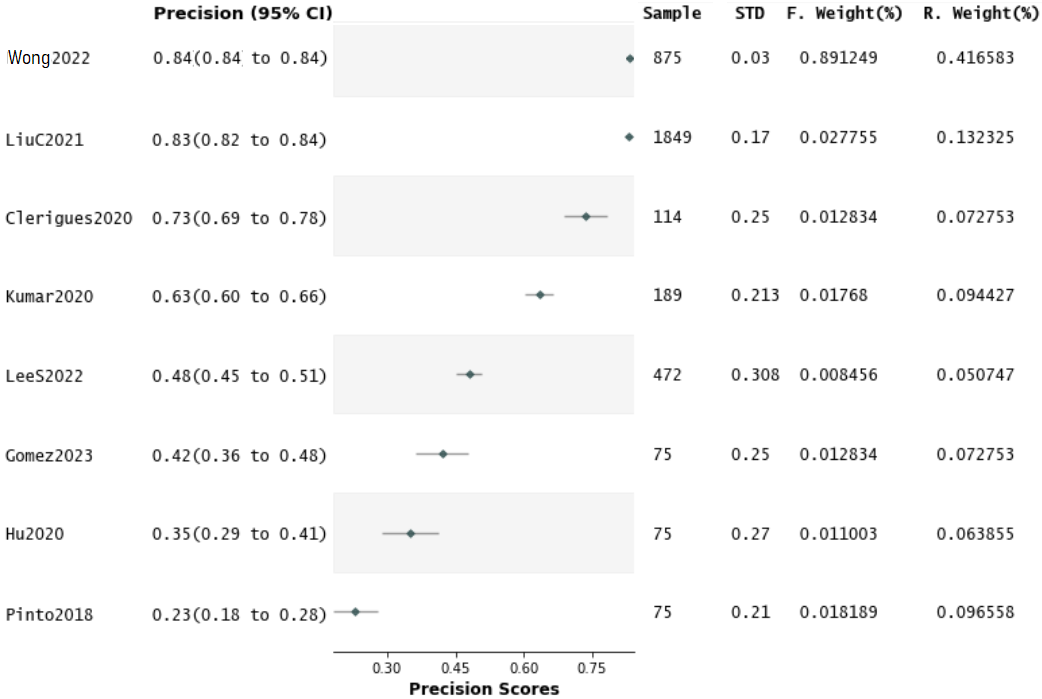


**Supplementary Figure 6.** Forest plot as part of the sensitivity analysis showing per study, the Precision score, standard deviation, sample size, and weight assigned from both the fixed-effects (F. Weight(%)) and random-effects (R. Weight(%)). Eight studies are represented. Diamonds represent point estimates. Horizontal lines going through them represent 95% confidence intervals. The line of null effect was not plotted, as in this case, there is no “expected” Precision score under the null hypothesis. Studies are ordered from highest to lowest mean Precision scores.


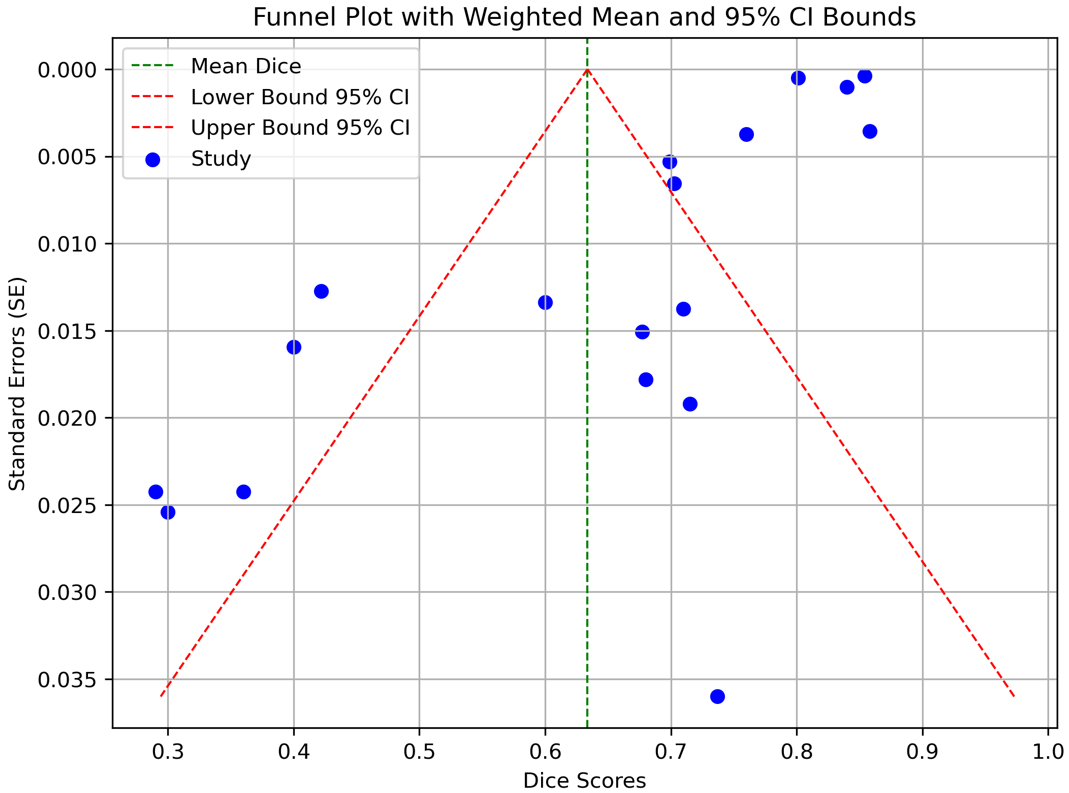


**Supplementary Figure 7.** The funnel plot shows high concentration of data points on the right side of the green dashed line indicating the weighted mean of reported mean Dice scores (12/18 studies). This suggests selective reporting of studies with high Dice. Specifically, the seven points with very low standard error (SE) (upper-right corner) represent the studies that were the most likely to weight the analyses thanks to their favourable results. The five data points with medium SE on the left side of the plot, just outside the lower 95% CI bound, indicate studies with effect estimates considerably lower than the overall weighted mean Dice. The six data points inside the 95% CI bounds (within the red diagonals) refer to studies whose mean Dice scores were consistent with overall findings.


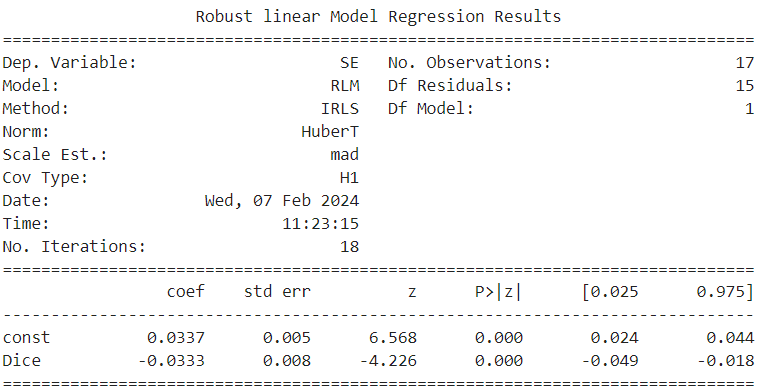


**Supplementary Figure 8.** Screenshot of the results from the Egger’s test, conducted to verify the findings form the funnel plot in terms of publication bias. The slope indicates the change in SE associated with a one-unit increase in Dice. Since it is negative (-0.033), it suggests that higher Dice is associated with lower SE, with statistical significance (p=0<0.05), thus confirming the previously observed publication bias.


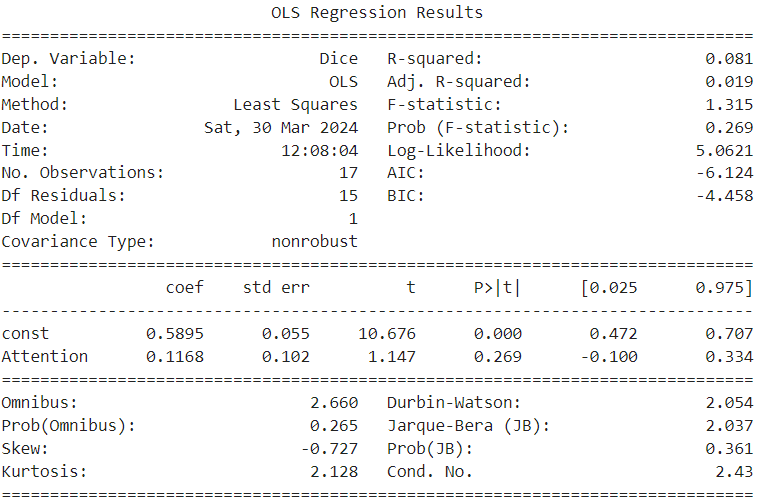


**Supplementary Figure 9.** Screen capture showing the summary statistics related to the meta-regression assessing the relationship between “attention mechanisms” and “Dice scores”: 8.1% of the variance in Dice scores is explained by the presence of attention (R-squared: 0.081). The slope indicating the change in Dice associated with the presence of attention is not statistically significant (0.117, p=0.27, 95% CI of the slope [-0.100,0.334]), therefore we cannot conclude that the presence of attention has a significant impact on the likelihood of high Dice.
